# Dynamic microfluidic single-cell screening identifies pheno-tuning compounds to potentiate tuberculosis therapy

**DOI:** 10.1038/s41467-024-48269-2

**Published:** 2024-05-16

**Authors:** Maxime Mistretta, Mena Cimino, Pascal Campagne, Stevenn Volant, Etienne Kornobis, Olivier Hebert, Christophe Rochais, Patrick Dallemagne, Cédric Lecoutey, Camille Tisnerat, Alban Lepailleur, Yann Ayotte, Steven R. LaPlante, Nicolas Gangneux, Monika Záhorszká, Jana Korduláková, Sophie Vichier-Guerre, Frédéric Bonhomme, Laura Pokorny, Marvin Albert, Jean-Yves Tinevez, Giulia Manina

**Affiliations:** 1 Institut Pasteur, Université Paris Cité, Microbial Individuality and Infection Laboratory, 75015 Paris, France; 2 Institut Pasteur, Université Paris Cité, Bioinformatics and Biostatistics Hub, 75015 Paris, France; 3 Institut Pasteur, Université Paris Cité, Biomics Platform, 75015 Paris, France; 4 Normandie Univ., UNICAEN, CERMN, 14000 Caen, France; 5 Institut National de la Recherche Scientifique—Armand-Frappier Santé Biotechnologie Research Centre, Laval, Quebec H7V 1B7 Canada; 6 Department of Biochemistry, Faculty of Natural Sciences, Comenius University in Bratislava, 842 15 Bratislava, Slovakia; 7 Institut Pasteur, Université Paris Cité, CNRS UMR3523, Epigenetic Chemical Biology Unit, 75015 Paris, France; 8 Institut Pasteur, Université Paris Cité, Image Analysis Hub, 75015 Paris, France

**Keywords:** Antimicrobial resistance, Microfluidics, Bacteriology, Phenotypic screening, Tuberculosis

## Abstract

Drug-recalcitrant infections are a leading global-health concern. Bacterial cells benefit from phenotypic variation, which can suggest effective antimicrobial strategies. However, probing phenotypic variation entails spatiotemporal analysis of individual cells that is technically challenging, and hard to integrate into drug discovery. In this work, we develop a multi-condition microfluidic platform suitable for imaging two-dimensional growth of bacterial cells during transitions between separate environmental conditions. With this platform, we implement a dynamic single-cell screening for pheno-tuning compounds, which induce a phenotypic change and decrease cell-to-cell variation, aiming to undermine the entire bacterial population and make it more vulnerable to other drugs. We apply this strategy to mycobacteria, as tuberculosis poses a major public-health threat. Our lead compound impairs *Mycobacterium tuberculosis* via a peculiar mode of action and enhances other anti-tubercular drugs. This work proves that harnessing phenotypic variation represents a successful approach to tackle pathogens that are increasingly difficult to treat.

## Abstract (web summary)

Tuberculosis is a major global health threat. Here, the authors develop a single-cell drug discovery approach and identify a compound that tunes bacterial phenotypic variation. This enhances the activity of anti-tubercular drugs against the pathogen.

## Introduction

The advent of antimicrobials revolutionized medical practice, saving countless lives, but bacterial pathogens were not slow to develop drug-evasion mechanisms^1^. Genetic changes occur rarely and confer a stable advantage to the whole population. Phenotypic changes occur regularly and confer a transient advantage to a subset of individuals but can have a positive effect on whole-population fitness^2,3^. The unique ability of microbes to rapidly diversify into phenotypic variants, such as drug-tolerant, persistent, viable but non-culturable, and non-growing but metabolically active cells, increases their chances of survival during antibiotic treatment^4–6^. Phenotypic changes associated with antibiotic-escape mechanisms are multifactorial and were also shown to foster the emergence of resistance^7,8^. Overall, the marked diversification potential of microbial pathogens, in addition to physicochemical barriers to drug penetration and inefficient host clearance, largely contributes to persistent and recurrent infections and is conducive to treatment failure^9,10^. As a result, nearly a century after the golden era, the world is running into an alarming post-antibiotic era, which is jeopardizing global-health stability^11^. This emphasizes the pressing need to tackle bacterial pathogens with original solutions, which reckon with the inherent and environment-driven phenotypic variation of clonal microbial populations^12,13^.

A leading example of public-health threat is *Mycobacterium tuberculosis*, which can persist for months up to years in the host despite protracted therapy and is responsible for about a quarter of the antimicrobial resistance emergency^14^. Although some new and repurposed drugs have been introduced in recent years^15,16^, tuberculosis treatment remains a slow and grueling process that requires improvement. Shortening treatment duration and decreasing relapse rates entail targeting drug-tolerant and persistent subsets, by either preventing their formation or hindering their survival mechanisms^10,12,17–19^. Nevertheless, the underlying limitation of conventional drug-discovery approaches, whether target- or cell-based, lies in the fact that they rely on reductionist assessments, with little-to-no resolution on minor cellular subsets, responsible for drug evasion^11,14,20^. Hence, the selection of more effective drug candidates should rely on broader decision criteria, including those based on the quantification of single-cell dynamics, aimed at leveraging phenotypic variation to enhance therapeutics.

Diverse instances of phenotypic variation related to growth, division, and gene expression, as well as their association with phenotypic drug tolerance, were reported in mycobacteria^21^. A relevant case of cell-to-cell variation concerns genotoxic stress, which can occur due to intrinsic replication errors or exogenous aggression, such as immune effectors and drugs^10^. Using a fluorescent reporter of RecA as a proxy for DNA damage response, we found that more than half of clonal mycobacterial cells, grown in stress-free conditions, experiences transient DNA damage events, measured as single-cell pulses of fluorescence, which largely resolve spontaneously. In addition, pulsing cells are more likely to die upon treatment with DNA-targeting drugs, as opposed to non-pulsing cells that survive significantly more^22^. Based on these findings, we hypothesized that manipulating RecA phenotypic variation might prevent drug-evasion mechanisms and weaken the mycobacterial population. To this aim, we sought to carry out a microfluidic dynamic single-cell screening for pheno-tuning compounds (PTC), which we refer to as µDeSCRiPTor. Our goal was to increase RecA expression levels and to decrease RecA cell-to-cell variation, to make clonal mycobacterial cells more homogeneously susceptible to standard treatment. However, the technology required to implement this approach did not exist.

Biocompatible microfluidic devices in conjunction with time-resolved microscopy are instrumental in capturing the behavior of live cells over space and time under tight environmental control, but they are complex to scale up for high-throughput applications^23^. Although digital microfluidics allows higher-throughput applications than continuous-flow microfluidics, encapsulation of small bacterial cells within hydrolipid droplets poses major limitations to both spatiotemporal resolution and environmental flexibility^24^. To overcome these drawbacks, we developed a scalable microfluidic cell-culture chamber^25^, which operates on gentle hydro-pneumatic trapping of cells and allows two-dimensional growth. Based on this microfluidic module, we had previously developed a five-condition device^25^, which was intended to expose bacteria to a gradient of concentrations of the same compound but was not suitable for screening applications.

Here, we present the development of a customized multi-condition microfluidic platform that enables us to test several compounds simultaneously, thus significantly increasing the experimental throughput. In short, we combine thirty-two pairs of microchambers that not only share a common microfluidic network but are also supplied via independent reservoirs, to be able to inject a different solution into each microchamber. With this platform, we carry out the first proof of concept of µDeSCRiPTor. By testing fewer than a hundred compounds, we identify four main hits that meet our criteria for a PTC compound, i.e., capable of pushing clonal cells into a RecA-induced state while reducing inter-cellular variation in RecA expression. Following an in-depth characterization of our best PTC hit M06, we show that it acts through a dual mechanism, which impairs both cellular integrity and DNA, with a marked increase in oxidative stress. With this original drug-discovery model we identify a compound that not only undermines the mycobacterial cell per se but also enhances existing drugs, holding promise for therapeutic development against tuberculosis and other infectious diseases.

## Results

### Microfluidic dynamic single-cell screening for pheno-tuning compounds (µDeSCRiPTor)

**Fig. 1:**
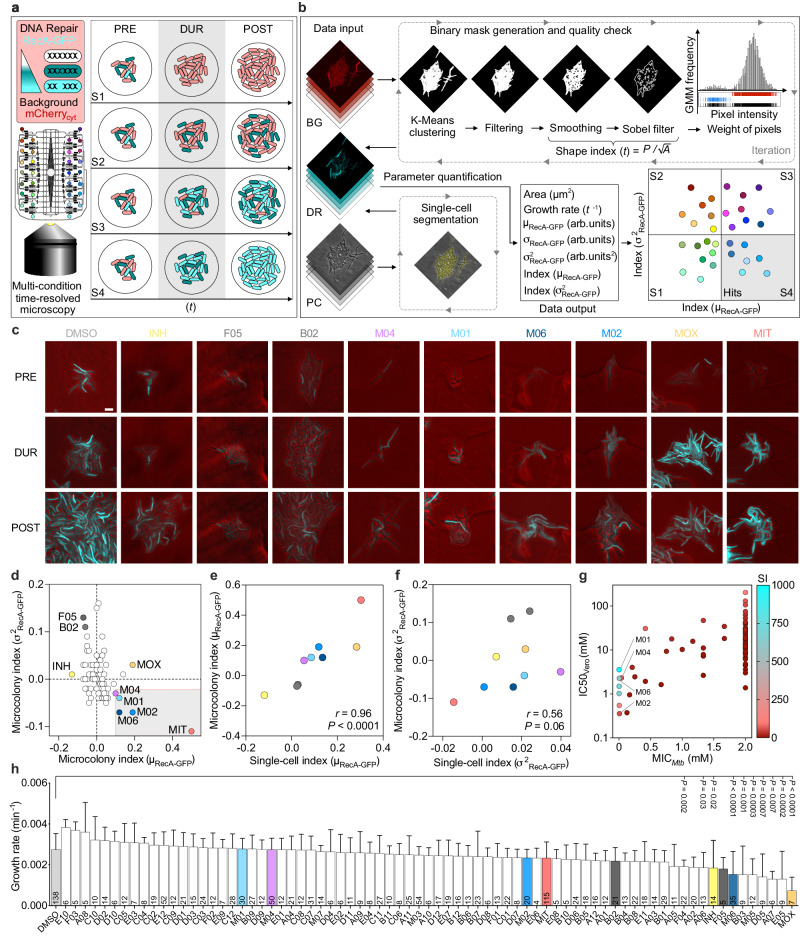
Rationale and outcome of µDeSCRiPTor. **a**, **b** Schematics of screening setup (**a**) and analysis workflow (**b**). Green-fluorescent reporter of DNA damage response (DR), expressing a cytosolic red-fluorescent marker as a background (BG). Green gradient denotes the magnitude of response to DNA damage. Bacteria are loaded into the 32-condition platform and are imaged before (PRE), during (DUR), and after (POST) exposure to PTC (gray shading). Four possible responses are depicted (S1 to S4), relative to changes in RecA fluorescence intensity and cell-to-cell variation (**a**). Three channels for BG and DR fluorescence and phase contrast (PC) are extracted from time-resolved image series. Black arrows indicate the direction of the analysis workflow and dashed rectangles and gray arrowheads indicate temporal reiteration (**b**). BG fluorescence was used to generate binary masks for whole-colony segmentation. Microcolony growth rate was calculated based on their area (*A*), DR due to drug exposure was assessed based on fluorescence values, considering both average (µ_RecA-GFP_) and variance (σ^2^_RecA-GFP_). PTC hits fall into S4 (gray shading). **c** Time-lapse images of *M. smegmatis* DR-BG reporter at the end of PRE, DUR, and POST stages. DR (cyan) and PC (red) channels are merged. Fluorescence is scaled to the brightest frame. Scale bar = 5 µm. **d** Size of the PTC effect (effect size) on both average fluorescence (µ_RecA-GFP_) and variance (σ^2^_RecA-GFP_) when comparing the DUR versus PRE stage. For each stage, at least *n* = 6 time points were measured per microcolony. Each molecule was replicated up to 8 times per experiment, and 15 independent experiments were performed (Supplementary Data 3). Negative control DMSO (dashed black lines), positive control MIT (red circle), and 20% of MIT effect (dashed red lines) are shown. PTC hits (gray shading). **e**, **f** One-tailed Spearman correlation between single-cell and microcolony indices of RecA-GFP mean intensity (**e**) and variance (**f**), color-coded as in **c** and **d**. **g** Average of MIC in *M. tuberculosis* and of IC50 in Vero cells of different PTC, and selectivity index (SI: IC50/MIC), *n* = 2 independent experiments. **h** *M. smegmatis* microcolony growth rate during treatment. The total number of microcolonies indicated in the bars, from at least *n* = 2 biologically independent experiments, expressed as mean ± SD. Significance by two-way ANOVA followed by Dunnett’s multiple-comparison test, *F*(75,1092) = 5.877, *P* < 0.0001. Source data are provided as a Source Data file.

We reported that RecA, a key enzyme for DNA damage repair in the SOS response^26^, is unevenly expressed in clonal mycobacterial cells in the absence of stress, as transient pulses occurring in more than half of the population, and is induced by different host-mimicking stresses^22^. RecA-pulsing cells are also more likely to die upon treatment with DNA-damaging agents, such as fluoroquinolones and mitomycin C (MIT). Based on these findings, we hypothesized that decreasing cell-to-cell variation, by exogenous induction of RecA, could make the population more susceptible to treatment. To this aim, we conceived the µDeSCRiPTor strategy, a time-resolved single-cell screening for compounds that fine-tune pre-existing variation in live mycobacterial cells, inducing functional phenotypic-changes in the population. As the first proof of concept, we used our *M. smegmatis* transcriptional reporter of *recA* (RecA-GFP)^22^, also carrying a cytosolic red-fluorescent marker (mCherry_cyt_), to screen for compounds that induce RecA-GFP fluorescence, aiming to sensitize mycobacterial cells to treatment and decrease RecA-GFP fluorescence variation, aiming to homogenize single-cell responses (Fig. 1a).

To implement our µDeSCRiPTor, we first constructed and characterized an original multi-condition microfluidic platform, suitable for long-term imaging of single mycobacterial cells and live-cell screening applications (Supplementary Fig. 1a; Supplementary Fig. 2; Supplementary Data 1; Methods). In this microsystem, bacteria grow as micrometer-sized colonies distributed on a two-dimensional (2D) plane, under weak mechanical compression and steady flow of medium inside 32 pairs of microchambers (Supplementary Figs. 1b−d; Supplementary Movie 1; Supplementary Figs. 2a−d). Each pair of microchambers can also be separately fed from 32 independent reservoirs in the absence of cross-contamination, allowing real-time toggling between environmental conditions. (Supplementary Fig. 1e; Supplementary Figs. 2e−l). We measured that solutions stored in the reservoirs undergo a dilution of about 2.5 folds during injection, due to the presence of a continuous flow from the main inlet port. We found no significant difference in microcolony growth rate among different microchambers and compared to our single-condition reference device^22^ (Supplementary Fig. 1c), regardless of the position within the microchamber (Supplementary Fig. 1d). Although the coefficient of variation of growth was slightly higher in this multi-condition prototype, likely due to the manual microfabrication process (Methods), the average growth rate was comparable to that of *M. smegmatis* in flask. In addition, neither single-cell doubling time nor RecA pulsing-expression was affected, confirming that growth inside the microchambers did not alter mycobacterial physiology (Supplementary Fig. 1f, g).

To assess the effect of compound treatment on our dual-fluorescent reporter (Supplementary Fig. 3a, b), we computed indices of RecA-GFP mean fluorescence (µ_RecA-GFP_) and fluorescence variance (σ^2^_RecA-GFP_) and estimated the effect sizes for those indices, by comparing the pre-exposure stage with the compound-exposure stage, upon normalization to the negative control group treated with DMSO (Fig. 1b; Methods). Large effect sizes were associated with strong changes in the fluorescence-derived indices upon treatment. Although a PTC could cause up to four distinct response scenarios (Fig. 1a, b), aiming to weaken the population, an ideal PTC was expected to cause a significant increase in the index (µ_RecA-GFP_) and a significant decrease in the index (σ^2^_RecA-GFP_) compared to DMSO.

We tested two categories of compounds (Supplementary Data 2). The first one included 65 fragments, low-molecular-weight organic compounds, which typically possess weaker affinities to disease targets but exhibit more efficient interactions with these targets as compared to larger molecules traditionally used in high-throughput screens. These fragments exhibited millimolar activity against mycobacteria^27^. The second one included 7 phenanthroline compounds (Supplementary Methods), 3 of which were described earlier^28^. These compounds were larger heterocyclic organic molecules derived from a phenanthroline scaffold with two carbonyl groups, sharing some structural similarities with fluoroquinolones and exhibiting micromolar activity against mycobacteria. We did not expect major absorption problems from polydimethylsiloxane (PDMS) forming the device, since the partition coefficients of the compounds were below the risk threshold^29^. The experimental setup was divided into three stages: 6 h of pre-growth in standard medium (PRE); 6 h of exposure to subinhibitory concentrations of candidate PTC or control molecules (DUR); and 6 h of washing (POST) (Fig. 1a, c; Supplementary Movie 2). The responses of single microcolonies during the screening were plotted in a two-dimensional graph displaying the relationship between the two indices of fluorescence, corresponding to four possible scenarios (Fig. 1a, b, d). As a positive control, we used MIT, which causes double-strand DNA breaks, resulting in significant induction of index (µ_RecA-GFP_) and significant reduction of index (σ^2^_RecA-GFP_). Thus, we regarded as relevant PTC hits all compounds falling into the lower-right quadrant, causing a phenotypic change not less than 20% of the effect caused by MIT (Fig. 1d; Supplementary Fig. 3c, d; Supplementary Data 3). All four hits were found to be phenanthroline derivatives. M02 and M06 caused the strongest and most significant effects in both indices, while M01 and M04 caused a significant increase in the index (µ_RecA-GFP_), and a moderate but not significant decrease in the index (σ^2^_RecA-GFP_). Most of the tested compounds did not induce major phenotypic changes toward the other three possible scenarios, except for the fragment compounds B02 and F05. These fragments caused a significant effect opposite to that of PTC hits, i.e., a significant decrease in index (µ_RecA-GFP_) and a significant increase of index (σ^2^_RecA-GFP_). We expected that PTC responsible for this second scenario would make bacteria less sensitive to treatment, and indeed found that subinhibitory concentrations of both B02 and F05 moderately increase the IC50 of some anti-tubercular drugs (Supplementary Fig. 4a–h). Treatment with the anti-tubercular drugs moxifloxacin (MOX) or isoniazid (INH) caused a sharp increase or decrease, respectively, in the index (µ_RecA-GFP_), with high levels of phenotypic variation. Although we had to infer single-cell behaviors from 2D microcolonies, due to the lack of automated tools for time-resolved segmentation of mycobacterial cells, we carried out separate manual analyses of individual cells on a targeted group of samples. We were able to show positive correlations between the results obtained from microcolonies and those obtained from single cells (Fig. 1e, f; Supplementary Fig. 3e−g). However, the correlation between indices (σ^2^_RecA-GFP_) was weaker due to the low number of single cells segmented, especially in the PRE stage (Fig. 1f; Supplementary Fig. 3g).

About 94% of the PTC tested showed a good selectivity index between the half maximal inhibitory concentration (IC50) in Vero cells and the minimum inhibitory concentration (MIC) in the tubercular pathogen, with particularly high values for the selected PTC hits, implying low cellular toxicity and high potency (Fig. 1g). However, the principle used to select PTC in *M. smegmatis* was not based on growth inhibition (Fig. 1h), but exclusively on RecA pheno-tuning (Fig. 1d). In conclusion, the µDeSCRiPTor strategy enabled us to select four compounds (Supplementary Fig. 3h) that met our expectations for PTC and were further analyzed.

### PTC hits induce DNA repair mechanisms in *M. tuberculosis* and interfere with DNA gyrase

**Fig. 2:**
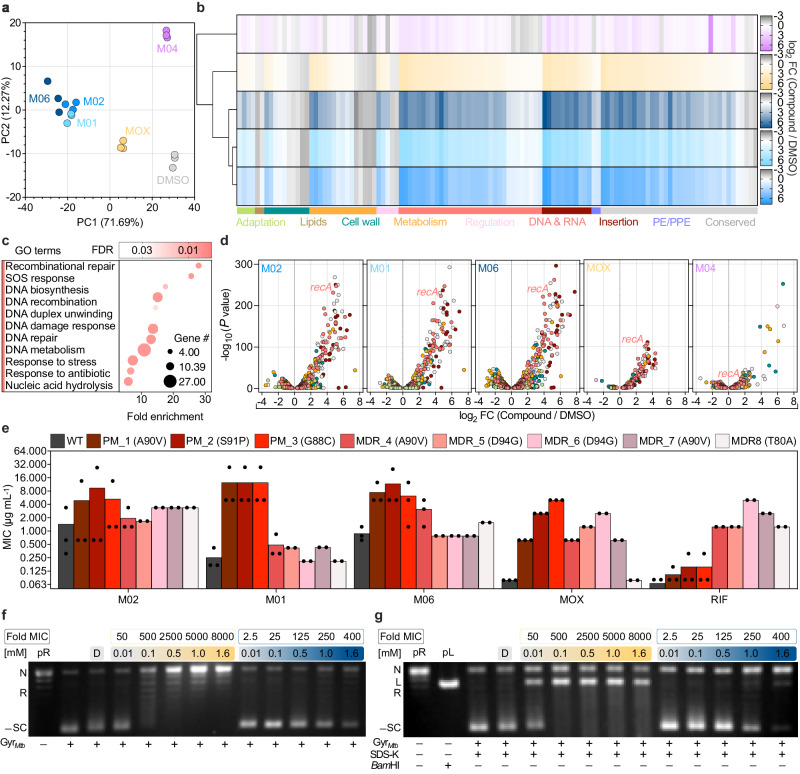
Transcriptome analysis of PTC-treated *M. tuberculosis* and effects on DNA gyrase. **a** Experimental variability (*n* = 3) from the first two components of a PCA, with percentages of variance associated with each axis. PC1 (biological variation) describes more than 70% of the total variance in the dataset. **b** Heat maps showing the log_2_ fold-change (FC) of genes that are differentially regulated in compound-treated versus DMSO-treated bacilli (color-coded as in **a**), based on a generalized linear model on the counts. Samples clustering of normalized data is shown. Genes are also grouped into color-coded functional categories (lower bars). **c** Gene Ontology (GO) enrichment analysis of 116 shared genes that were differentially regulated upon treatment with PTC hits or MOX compared to DMSO. Complete GO biological processes, including electronic and manually curated annotations, were identified by overrepresentation test against all *M. tuberculosis* genes in PANTHER database. Raw *P*-values were determined by Fisher’s exact test, with false discovery rate (FDR < 0.05) correction by the Benjamini–Hochberg method. Bubble chart reports the most specific functional categories sorted by fold enrichment. The full hierarchies of significantly enriched functional categories are also available (Supplementary Data 5). **d** DEGs in compound-treated compared to DMSO-treated bacilli ranked in volcano plots according to -log_10_ adjusted *P*-value (Wald test *P*-values with Benjamini–Hochberg correction) as a function of log_2_ FC ratio. Functional categories are color-coded as in **b**. **e** MIC of clustered PTC and anti-tubercular drugs in *M. tuberculosis* WT, *gyrA* point mutants (PM 1 to 3), and multi-drug resistant strains (MDR 4 to 8). GyrA amino-acid changes are indicated in brackets. Bars represent the mean of at least *n* = 2 biologically independent experiments. **f**, **g** Representative supercoiling (**f**) and cleavage (**g**) assays of relaxed pBR322 (pR) by *M. tuberculosis* DNA gyrase (Gyr_*Mtb*_) in the absence or presence of DMSO (D) and with increasing concentrations of MOX (orange gradient) and M06 (blue gradient). Experiments were repeated twice independently with similar results. Correspondence between concentration and MIC is also boxed. Nicked (N), relaxed (R), and negatively supercoiled (-SC) DNA in the pR control plasmid, and linearized (L) DNA in the pL control plasmid are indicated. SDS and proteinase K (SDS-K) were used to denature DNA-bound Gyr_*Mtb*_ (**g**). Source data are provided as (Supplementary Data 4) and Source Data file.

To probe the effect of the identified PTC hits on the mycobacterial cell, we carried out a whole-transcriptome analysis in *M. tuberculosis*. We investigated the rapid transcriptional remodeling of axenic cultures upon treatment with PTC or MOX (10-fold MIC) for approximately a quarter of the generation time. Principal Component Analysis (PCA) revealed that biological variability was the main source of variance among our datasets and that three out of four PTCs, namely, M01, M02, and M06, clustered together, implying a similar function (Fig. 2a). This was confirmed by differential analysis of gene expression in PTC- relative to DMSO-treated bacteria, as clustered PTC shared 898 differentially expressed genes (DEGs), whereas all four PTC shared 317 DEGs (Fig. 2b; Supplementary Data 4). Consistent with our screening results (Fig. 1), PTC and MOX were found to share 116 DEGs, which were associated with DNA damage response and repair (Fig. 2c; Supplementary Data 5). However, the extent of *recA* transcriptional induction was more pronounced in response to the three clustered PTCs as compared to both MOX and M04 (Fig. 2d). M04 also turned out to be quite insoluble, leading us to not prioritize this hit. Thus, we focused on the clustered PTC and especially on M06, which had better physicochemical properties and a more promising activity profile in combination with other anti-tubercular drugs (Supplementary Fig. 5a).

It is worth noting that all PTC hits share a portion of their structure, which evokes the bicyclic core of quinolones responsible for binding to DNA gyrase^30^. Thus, we probed whether DNA gyrase could be the target. We first tested a panel of *M. tuberculosis* strains carrying the most frequent *gyrA* mutations conferring moxifloxacin resistance. While laboratory strains harboring *gyrA* point-mutations were moderately resistant to PTC^28^, all multi-drug resistant clinical isolates were found to be fully susceptible to PTC (Fig. 2e; Supplementary Data 6). Next, we directly tested the effect of M06 against purified *M. tuberculosis* DNA gyrase in vitro. As opposed to MOX, which caused strong inhibition of the negative supercoiling activity of DNA gyrase as expected, M06 caused weak inhibition of DNA gyrase at a ten-fold higher concentration (Fig. 2f). Likewise, MOX promoted plasmid DNA breaks via stabilization of gyrase-DNA cleavage complex starting from low µM concentration, whereas M06 caused limited DNA rupture only at mM concentration (Fig. 2g). Additionally, docking analysis predicted that M06 could occupy the active site of DNA gyrase, interacting via hydrogen bond of the hydroxyl group (O4) and hydrophobic contact (C14) with the residue D94, which is in contact with the magnesium ion^30^ (Supplementary Fig. 5b−e).

PTC hits also caused a broader transcriptional response different from that of MOX (Supplementary Fig. 6a−e; Supplementary Data 5). Specifically, the three clustered PTCs caused marked repression of the electron transport chain, cellular respiration, glycolysis, and tricarboxylic acid cycle, as well as of fatty acid and lipid metabolism (Supplementary Figs. 6c−e). Overall, these results are suggestive of a broader and more severe mode of action of the clustered PTC against the mycobacterial cell compared with MOX.

### The arylamine N-acetyltransferase NAT is associated with the mode of action of PTC

**Fig. 3:**
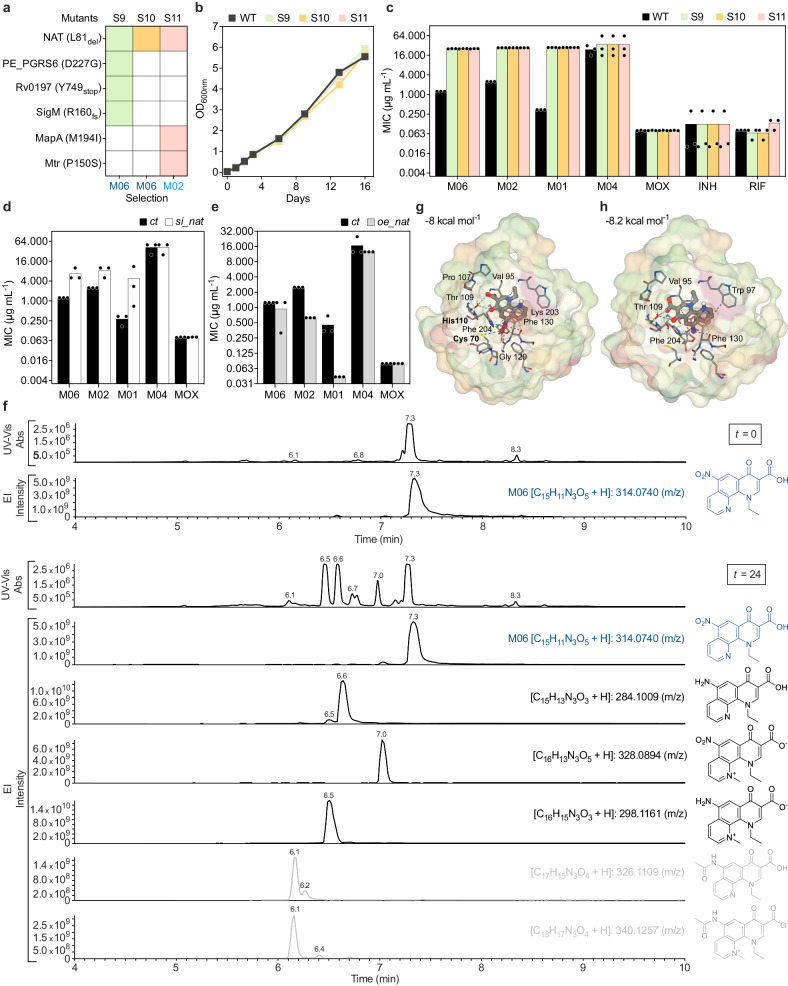
Characterization of the mechanism of action of clustered PTC. **a** Summary of variant-calling analysis of three spontaneous mutants sharing the same mutation in *rv3566c* gene (*nat*). The compound on which the mutants were isolated is indicated. **b** Growth kinetics of *M. tuberculosis* wild type (WT), S9, S10, and S11 mutants. Symbols represent the mean of *n* = 2 independent replicates. **c**–**e** MIC of PTC and anti-tubercular drugs in *M. tuberculosis* WT and *nat* mutants (**c**); following silencing of *nat* (*si_nat*) (**d**); and overexpression of *nat* (*oe_nat*) (**e**) and in related controls (*ct*). Bars represent the mean of *n* = 3 biologically independent experiments. **f** Representative spectra at 270 nm (UV) and extracted ion (EI) chromatograms of supernatant from *M. tuberculosis* treated with M06 (15-fold MIC) at the onset of treatment and after 24 h, *n* = 2 biologically independent experiments. *x*-axes indicate the time after injection (min). *y*-axes indicate intensities (arb. units). Chemical structures, formulas, and the mass-to-charge ratio of M06 and its metabolites are shown next to the relevant peaks. **g**, **h** In silico docking poses of CPK-colored M06 (**g**) and its amino derivative (**h**) in the catalytic pocket of NAT. Binding affinity is indicated (kcal mol^−1^). Predicted amino-acid residues in hydrophobic contact (dark gray) and linked by either weak (light gray) or strong (blue) hydrogen bonds with ligand atoms are shown. Bolded residues are part of the catalytic triad (**g**). Source data are provided as a Source Data file.

To further clarify the mechanism of action, we searched for mutants resistant to the PTC hits, and isolated two on M06 and two on M02 (Supplementary Data 7). Interestingly, three out of four shared the same mutation on the *rv3566c* gene (Fig. 3a), which encodes the arylamine N-acetyltransferase (NAT). NAT is a highly conserved xenobiotic metabolizing enzyme, which uses acyl donors, such as acetyl-CoA or propionyl-CoA, to acetylate diverse heterocyclic amines^31^. For instance, NAT was shown to antagonize the INH-activating role of the catalase-peroxidase KatG (Supplementary Fig. 7a), by acetylating this prodrug^32^. *M. tuberculosis rv3566c* is located downstream of an operon implicated in lipid catabolism (Supplementary Fig. 7a) and was associated with mycolic acids biosynthesis, cell-wall integrity, and intracellular survival^31,33^. Additionally, deletion of *nat* in *Mycobacterium bovis* BCG was shown to retard the entry into exponential growth-phase^33^. Therefore, we sought to clarify whether NAT was responsible for the inactivation or the activation of PTC or if it was the target. In all three isolated mutants, NAT was devoid of leucine 81 (Fig. 3a), which is implicated in enzyme structure stability^34^, and is located within the active site between two critical catalytic residues (Supplementary Fig. 7b). NAT mutants did not experience any growth defect (Fig. 3b) but were cross-resistant to the clustered PTC (Fig. 3c). To further probe the role of NAT into the mechanism of action of PTC, we generated an inducible knock-down (*si_nat*) and an overexpressing (*oe_nat*) *M. tuberculosis* strain. Transcriptional repression of *nat* caused moderate but significant growth defect and resistance to the three clustered PTCs, but not to other anti-tubercular drugs tested, supporting the hypothesis that NAT is the cellular target, and not the inactivating enzyme (Fig. 3d; Supplementary Fig. 7c−e). This was consistent with a transcriptional decrease of *nat* in PTC-stressed bacilli (Supplementary Data 5). In contrast, transcriptional induction of *nat* did not cause PTC resistance (Fig. 3e), implying a more complex mechanism^35^.

To determine whether NAT is the target or the enzyme that activates the top PTC hit M06, contingent on the reduction of the nitro group at position N3 (Supplementary Fig. 5b), we carried out liquid chromatography and mass spectrometry analysis of both cell extract and supernatant of *M. tuberculosis* treated with M06 (Supplementary Data 8). We obtained similar metabolite patterns although there were lower absolute amounts inside the cell and less of the original molecule (Fig. 3f; Supplementary Fig. 7f). After 24 h of M06 treatment, we found about 5% of the original molecule intracellularly and more than a quarter extracellularly, with three major metabolites: 3 to 10% methylated at position N1; 9 to 28% nitro-reduced at position N3; and 10 to 30% of methylated and nitro-reduced variants. Importantly, we found only a negligible fraction of acetylated form at position N3 both inside and outside the cell, supporting the assumption that M06 is not a substrate of NAT. We also carried out a docking analysis of M06 and its nitro-reduced variant with NAT^36^. Similar to the INH substrate (Supplementary Fig. 7g), both M06 and its reduced metabolite fell into the active site of NAT (Fig. 3g, h). However, while M06 was predicted to bind two of the three catalytic residues (Fig. 3g), its amino derivative was predicted to lose these interactions (Fig. 3h), suggesting that this metabolite is unlikely to be a substrate of NAT. Additionally, the synthesized amino-derivative of M06 is more than ten times less effective than the original molecule, suggesting that nitro-reduction is leveraged by the pathogen to inactivate M06. Collectively, these results led us to conclude that NAT is more likely to be the target of M06, rather than the enzyme implicated in the metabolization of this PTC.

### M06 affects the composition of the mycobacterial cell envelope and causes oxidative stress

**Fig. 4:**
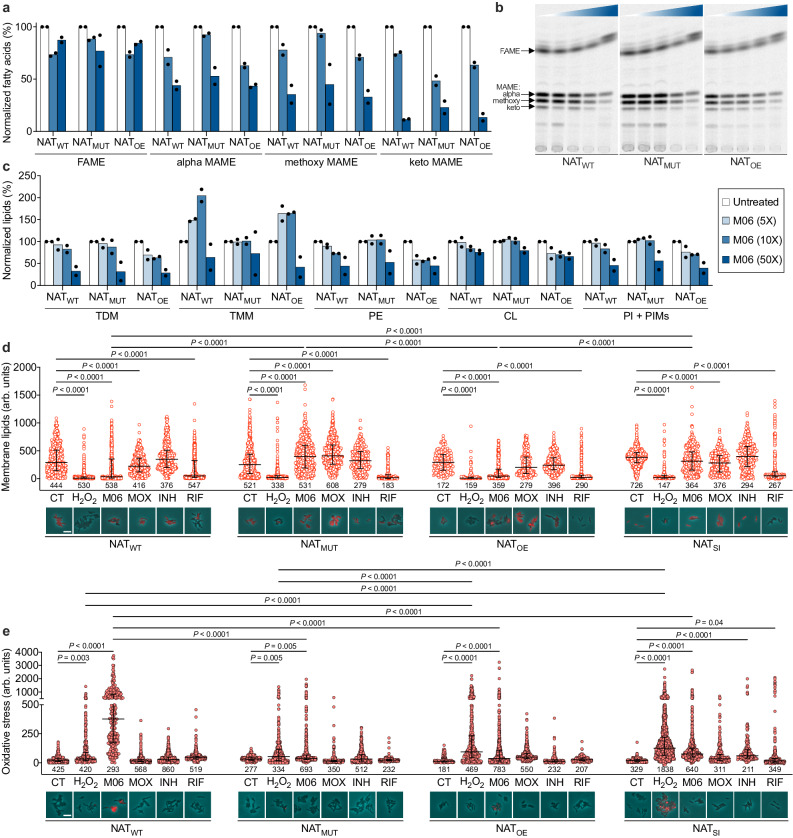
Lipid profiling and analysis of oxidative stress upon M06 treatment. **a**, **c** Quantification of fatty acid methyl esters (FAME) and different types of mycolic acid methyl esters (MAME) (**a**), and of trehalose esters of mycolates (TDM; TMM), phospholipids (PE; CL), and population of PI and PIMs (**c**) in exponentially growing *M. tuberculosis* WT (NAT_WT_); S10 mutant (NAT_MUT_); and NAT overexpressing strain (NAT_OE_), treated with different concentrations of M06 relative to the MIC for 24 h (inset). Data are normalized to untreated samples and bars represent mean, *n* = 2 biologically independent experiments. **b** Representative TLC of extracted FAME and MAME (arrows), developed in *n*-hexane:ethyl acetate (95:5, v/v, 3 runs). Experiments were repeated twice independently with similar results. The color gradient indicates from left to right the absence or increasing concentrations of M06 equal to 5x, 10x, 25x, and 50x MIC (1.25 µg/mL). **d**, **e** Single-cell analysis and representative snapshot images of *M. tuberculosis* expressing different variants or different levels of NAT after 24-h exposure to DMSO (CT), H_2_O_2_ (30 mM), or different drugs (10-fold MIC). Bacteria were stained with either FM464 for cell-envelope lipids (**d**) or with CellROX for oxidative stress (**e**) and imaged by phase contrast (cyan) and fluorescence (red). Fluorescence images are scaled to the brightest frame. Scale bars = 5 μm. The total number of bacilli is shown at the bottom of the graphs, *n* = 2 biologically independent experiments. Error bars represent the median with an interquartile range. Significance by two-way ANOVA followed by Tukey’s multiple comparisons test, 95% confidence interval: *F*(23, 8374) = 129.3, *P* < 0.0001 (**d**); *F*(23, 9697) = 128.6, *P* < 0.0001 (**e**). The complete analysis is available on Source Data. Source data are provided as a Source Data file.

Given the substantial transcriptional remodeling related to energy and lipid metabolism triggered by M06 (Supplementary Fig. 6e) and the implication of NAT in lipid metabolism^33^, we probed whether M06 could alter the composition of the cell envelope in *M. tuberculosis*. We fed bacilli with radiolabeled acetate and analyzed fatty acids and lipids by thin-layer chromatography (TLC). We found no differences in the TLC profiles of strains expressing wild-type or mutated NAT variants, or overexpressing NAT, suggesting that deletion of leucine 81 does not affect the physiological role of NAT (Supplementary Figs. 8a−d). High concentrations of M06 caused a significant decrease in mycolic acids, especially of the keto form in all three strains (Fig. 4a, b). These changes did not occur under MOX treatment, while, as expected, INH caused marked inhibition of all forms of mycolic acids and accumulation of fatty acids (Supplementary Fig. 9a). TLC analysis of extractable lipids revealed a significant decrease in phosphatidylethanolamine (PE), phosphatidylinositol (PI), phoshpatidylinositol mannosides (PIM), as well as trehalose dimycolate (TDM) in all strains, and accumulation of trehalose monomycolate (TMM) except in NAT-mutant strain (Fig. 4c; Supplementary Fig. 9b, c). Accumulation of TMM indicates impaired function of the MmpL3 transporter, which translocates this lipid through the plasma membrane. Indeed, the same phenotype was shown both after direct inhibition of MmpL3^37^, and in the presence of compounds that indirectly affect the membrane^38–40^. Moderate accumulation of TMM was also observed after treatment with MOX (Supplementary Fig. 9b, c). These results were confirmed at the single-cell level, as bacilli treated with M06 and, to a much lesser extent, with MOX showed decreased staining of the lipid content of the cell envelope, except when NAT was mutated or silenced (Fig. 4d; Supplementary Fig. 10a). Incidentally, treatment with rifampicin (RIF), which primarily inhibits the RNA polymerase, also caused significant perturbation of lipid staining (Fig. 4d), consistent with previous proteomic findings^41^.

Cell-wall alterations can result from both inhibition of synthesis and direct degradation. Interestingly, transcriptomic analysis revealed significant deregulation of genes involved in detoxification reactions upon M06 treatment (Supplementary Data 5). We further confirmed this by targeted gene expression analysis and showed that M06 causes deregulation of genes responsible for antioxidant defense, including increased *katG*, which protects against reactive oxygen and nitrogen intermediates; decreased *trxB1*, important for redox balance; and decreased *ahpE*, *ephA*, and *ephD*, involved in detoxification from organic peroxides and rescue of oxidized lipids (Supplementary Fig. 9d). Conversely, NAT mutant strains treated with M06 maintained unchanged levels of genes related to detoxification from lipid peroxidation, implying their lower susceptibility to this PTC. To check whether M06 induced oxidative stress, we carried out a single-cell snapshot analysis. We confirmed that *M. tuberculosis* exposed to M06 shows significantly higher levels of oxidative stress, which was not the case for moxifloxacin or other anti-tubercular drugs tested, and was significantly lower in *nat* mutant, *nat* overexpressing, and *nat* silenced strains (Fig. 4e; Supplementary Fig. 10b). This supports the role of NAT as the target of M06. Additionally, the combination of ROS scavengers with M06 did not inhibit M06 potency in vitro, suggesting that ROS production is presumably a secondary effect of M06 treatment (Supplementary Fig. 8e, f). Interestingly, deregulation of NAT levels was also found to be associated with increased oxidative stress following exposure to hydrogen peroxide. This opens the possibility that NAT may be involved in oxidative stress tolerance, because of the two cysteine residues present in its active site, which may serve a redox balancing role acting as nucleophiles^42^. Finally, when *nat* was repressed, bacilli experienced significantly higher levels of oxidative stress following treatment with both INH and RIF, indicating a nexus between *nat* inhibition and potentiation of these drugs, possibly associated with increased permeabilization. Overall, these results indicate that NAT may be a target of M06, resulting in impaired integrity of the mycobacterial cell envelope. However, we cannot yet rule out the possibility that NAT may be involved in a mechanism of resistance to oxidative stress following treatment with this PTC.

### The top PTC M06 shows promise in potentiating existing anti-tubercular drugs

**Fig. 5:**
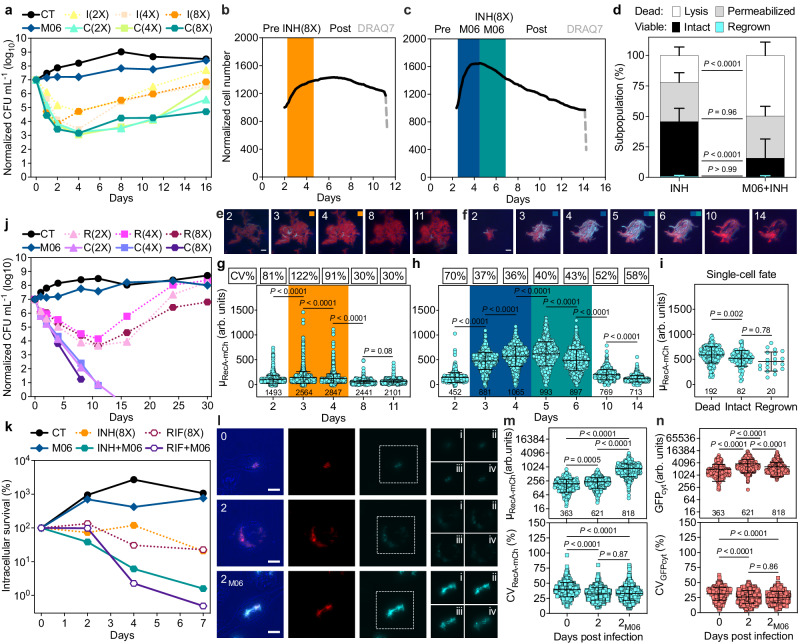
Extracellular and intracellular activity of M06 in combination with anti-tubercular drugs. **a**, **j** *M. tuberculosis* growth (CT, circles) and efficacy of M06 alone (1-fold MIC, diamonds), INH (I, **a**) and RIF (R, **j**) alone (dotted lines) or in combination with M06 (C, solid lines). Concentrations relative to the MIC in brackets (different symbols). Symbols represent the mean of at least *n* = 2 biologically independent experiments. **b**–**d** RecA-mCherry_GFP_cyt_ reporter during time-lapse microscopy: INH (0.2 µg/mL, orange), *n* = 981 bacilli (**b**); M06 (1.25 µg/mL, blue) and M06-INH combination (green), *n* = 1906 bacilli (**c**), from 3 independent cultures. Stages above the graphs. Fractions of subpopulation fates (**d**), *n* = 8 microcolonies (INH) and *n* = 11 microcolonies (M06 + INH), over 3 independent cultures. Data are mean ± SD. Significance by two-way ANOVA followed by Šidák’s multiple-comparison test, 95% confidence interval, *F*(3, 68) = 56.97, *P* < 0.0001. **e**, **f** Repres**e**ntative time-lapse microscopy images, color-coding as in **b** and **c**, respectively. Phase contrast (blue), RecA-mCherry (cyan), and GFP_cyt_ (red) are scaled to the brightest frame and merged. Numbers represent days. Scale bars = 5 μm. **g**–**i** Single**-**cell fluorescence under the conditions as in **e** and **f** (**g**, **h**), and after M06 exposure (**i**). The total number of bacilli is shown at the bottom of the graphs, *n* = 3 independent cultures. Error bars represent mean ± SD. The coefficient of variation (CV) is boxed. Significance by two-way ANOVA followed by Tukey’s multiple comparisons test, 95% confidence interval: F(4, 8586) = 579.9, *P* < 0.0001 (**g**); *F*(6, 4689) = 939.7, *P* < 0.0001 (**h**); *F*(2, 100) = 7.327, *P* = 0.001 (**i**). **k** Intracellular *M. tuberculos****i****s* growth (CT), and efficacy of M06 alone (1-fold MIC), INH, and RIF alone (8-fold MIC, dotted lines), or in combinations with M06 (solid lines). Symbols represent mean, *n* = 4 independent experiments. **l** Representative images of THP−1 macrophages infected with RecA-mCherry_GFP_cyt_ reporter. Numbers represent days post-infection; M06 (1-fold MIC). Bright field (blue), GFP_cyt_ (red), and RecA-mCherry (cyan) are merged or separated. Z-sections i to iv are shown (dashed boxes). Scale bars = 10 µm. **m**, **n** Intracellular RecA-mCherry (**m**) and GFP_cyt_ (**n**) intensity and CV. The total number of intracellular foci is shown at the bottom of the graphs, *n* = 4 independent experiments. Error bars represent mean ± SD. Significance by Kruskal-Wallis’ test followed by Dunn’s multiple-comparison test, 0.05 alpha-threshold. Source data are provided as a Source Data file.

To examine whether pheno-tuning RecA expression was ultimately able to increase the sensitivity of *M. tuberculosis* to treatment, we tested the highest concentration of our top PTC M06 that had no bactericidal action per se (Supplementary Fig. 11a) in combination with a few standard antitubercular drugs^16^. Pharmacodynamics were estimated via colony forming units (CFU) quantification for a minimum of two weeks up to a maximum of six weeks, or until growth rebound was observed, without replenishing the drug during the experiment. As expected for a drug that is presumed to inhibit NAT, the association of M06 with INH in vitro decreased bacterial survival by up to 2 logs compared with INH alone (Fig. 5a). Although growth rebound was not prevented even at higher concentrations of INH, doubling the amount of M06 in combination with a low dose of INH (2-fold MIC) improved its efficacy, resulting in a 4-log decrease in CFU (Supplementary Fig. 11a). Next, we carried out time-resolved microfluidic microscopy with a RecA fluorescent-reporter strain (Supplementary Movies 3 and 4; Supplementary Fig. 12a). We found that pretreating *M. tuberculosis* with M06 (1-fold MIC) followed by the combination with high-dose INH (8-fold MIC) caused significant decrease in intact bacilli and significant increase in mortality compared to treatment with INH alone (Fig. 5b−d). This mainly resulted from increased cell lysis and cell-envelope permeabilization, and from a moderate decrease in the fraction of bacilli able to resume growth once drugs were removed. We also confirmed that pretreating *M. tuberculosis* with M06 caused significant single-cell induction of RecA and reduction of cell-to-cell variation, which was not the case for INH alone (Fig. 5e−h). Additionally, higher RecA levels upon M06 exposure were associated with increased susceptibility of *M. tuberculosis* to INH (Fig. 5i). In contrast, M06 had no enhancing effect on the other cell-wall targeting drug ethambutol (Supplementary Fig. 11b). Interestingly, M06 exerted the strongest potentiation on RIF, causing a decrease in CFU up to 3 logs after a week of treatment compared with RIF alone, down to below the detection limit by two weeks of treatment irrespective of the RIF dose (Fig. 5j). Furthermore, lack of growth rebound confirmed complete sterilization of the axenic culture. M06 also showed an additive effect with MOX (Supplementary Fig. 11c), consistent with their joint activity against DNA gyrase (Fig. 2), and with downregulation of *mfpA*, which protects DNA gyrase from quinolones^43^ (Supplementary Fig. 11d; Supplementary Data 5).

Next, we tested the effect of M06 alone and in combination with INH and RIF intracellularly, by both standard CFU and single-cell quantification. After one week of infection, in the presence of low-dose M06 and high doses of either INH or RIF, we measured a decrease in intracellular CFU of about one and two logs, respectively, with a downward trend for both combinations versus companion drugs alone (Fig. 5k). As for single-cell analysis of intracellular efficacy, we infected macrophages with a fluorescent-reporter strain of ribosomal expression^6^ (Methods; Supplementary Fig. 11e, f). After six days of infection, we found that low-dose M06 caused a significant decrease in both infected macrophages and bacterial burden (Supplementary Fig. 11g−j), consistent with the essentiality of NAT in macrophages^33^. While the combination of M06 with low-dose INH further decreased both the fraction of infected macrophages and the relative bacterial burden compared with INH alone (Supplementary Fig. 11g, i), the combination of M06 with low-dose RIF significantly decreased only the fraction of infected macrophages but had a similar impact on the bacterial burden per macrophage compared with RIF alone (Supplementary Fig. 11h, j). This might hinge on the fact that the strongest effect of M06-RIF combination becomes apparent only after about two weeks of treatment in vitro (Fig. 5j). Intracellular bacilli also showed decreased metabolic activity in the presence of M06 alone, which was more pronounced in combination with both INH and RIF, as compared to companion drugs alone (Supplementary Fig. 11k, l). Finally, macrophage mortality was higher under M06-INH combination and lower under M06-RIF combination (Supplementary Fig. 11m, n).

We probed whether M06 intracellular efficacy is also associated with RecA pheno-tuning, and whether the host-cell environment could contribute to this phenomenon since RecA expression is triggered by diverse host-mimetic conditions in vitro^22^. To check this, we infected macrophages with a translational reporter of RecA, also expressing a constitutive control marker (Fig. 5l). We found that not only *M. tuberculosis* significantly induces RecA after two days of infection, but that this induction is about 4-fold higher in cells treated with low-dose M06 (Fig. 5m, n; Supplementary Fig. 12b, c). In contrast, RecA fluorescence variation within intracellular infectious foci dropped significantly after two days of infection both in the absence and in the presence of M06, similar to the control marker. This suggests that the intracellular environment may promote the anti-tubercular effect of M06.

**Fig. 6:**
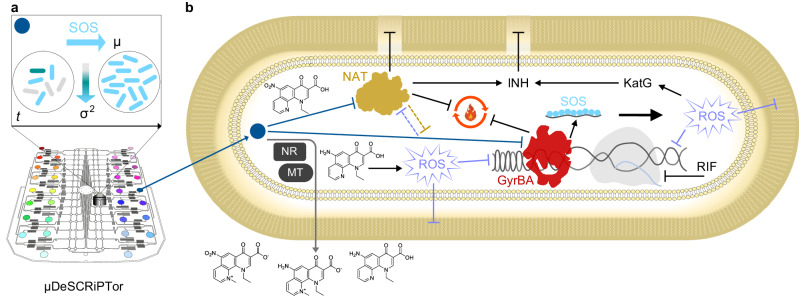
Schematic of µDeSCRiPTor and M06 mode of action. **a** Schematic of the microfluidic dynamic single-cell screening for PTC (colored circles), based on induction of the SOS DNA damage response (light-blue arrow) and decrease in cell-to-cell variation (gradient arrow). The growth and phenotypic transition of rod-shaped bacteria over time (*t*) is also illustrated. The main PTC hit M06 is shown as a blue circle. **b** Schematic of the expected mode of action and drug potentiation effect of M06. Blunt arrows indicate inhibition and sharp arrows indicate induction. Blue and gray sharp arrows show the entry of M06 into the cell and the exit of its main metabolites, respectively. Bioprocessing of M06 is carried out by unknown nitroreductases (NR) and methyltransferases (MT). Nitroreduction decreases M06 potency and is a likely source of oxidative stress^64^. Reactive oxygen species (ROS) damage lipids, DNA, and may affect NAT (lilac flat arrows). M06 is presumed to inhibit NAT, thus affecting the composition and stability of the mycobacterial cell envelope and impairing the energy metabolism (black flat arrows). NAT might otherwise be implicated in oxidative stress tolerance (orange flat arrow). M06 inhibits DNA gyrase causing DNA breaks. The presence of single-stranded DNA triggers the SOS response. Overall, impairment of cellular energy, lipid, and DNA metabolism might induce additional oxidative stress, which further affects different macromolecules and processes in the cell. Metabolic remodeling and induction of oxidative stress response help the cell countering oxidative damage^61^. This includes the upregulation of the catalase peroxidase KatG, which activates the anti-tubercular drug INH^32^. NAT inhibition by M06 further contributes to INH activation, destabilizing the cell integrity. On the other hand, M06-mediated inhibition of negative DNA supercoiling in the presence of the anti-tubercular drug RIF, which inhibits the RNA polymerase, further impairs DNA topology, preventing both DNA replication and transcription^67^, with lethal consequences for the mycobacterial cell.

Collectively, these findings prove that the µDeSCRiPTor strategy enabled us to identify, via RecA pheno-tuning, a promising compound that undermines *M. tuberculosis* through a multifactorial mode of action, and that potentiates other anti-tubercular drugs (Fig. 6a, b).

## Discussion

The discovery of new effective antimicrobials is a complex and inefficient process in need of renewal^11,14,20^. Despite the critical role of microbial phenotypic variation in treatment failure, this phenomenon is hardly included in conventional models of drug discovery due to technical hindrances, significantly limiting their potential^2,10,17,19^. To address this issue, we conceived the µDeSCRiPTor strategy, which is based on multi-condition microfluidics, to dynamically capture phenotypic changes via spatiotemporal imaging of individual cells within microcolonies. We proved the full potential of our strategy on mycobacterial cells, based on our earlier finding that pre-existing phenotypic variation in DNA damage response is associated with differential drug susceptibility at the subpopulation level^22^. However, this approach can be virtually applied to any instance of phenotypic variation that is critical to a given microbial pathogen to endure^9,10^, aiming to induce a homogeneous state of hypersensitivity. Although our initial goal was a proof of concept to probe changes in a fluorescent reporter upon exposure to different compounds, we were already able to identify a promising PTC that deserves further consideration for optimization and drug development.

The advantage offered by microfluidic technologies for studying the dynamics of single-cell behaviors comes either at the expense of throughput or at the expense of resolution and versatility^23^. To fill these gaps, we customized a multi-condition microfluidic platform, whose peculiarity lies in the combination of continuous flow, typical of passive devices, with pneumatic valves, typical of active devices^24^. Given the atypical aspect ratio of the microchambers featured in this platform, they can be actuated with pressures significantly lower than those used for on-off micromechanical valves^44^, generating a gentle pneumatic cell-trapping mechanism^25^, which is compatible with the flow of nutrients and cell viability. Overall, this platform allowed us to maintain high spatiotemporal resolution of single-cell behaviors and maximum control over environmental dynamics, as well as to increase the experimental throughput with respect to our former gradient platform^25^. However, the microfabrication process of this PDMS-based prototype includes several manual steps and currently has a success rate of about 60-70%, mainly due to adhesion defects between different layers that can cause fluid leaks. Additionally, the percentage of bacterial cells growing averaged 76%, about 10% less than for simpler devices^22^. This was most likely due to patchy adhesion of the flexible PDMS membrane to glass, retarding access to nutrients. Thus, we had to reject all movies containing bacteria that were not growing from the beginning of the experiment, that were growing in 3D, or moving due to membrane instability, and those with too many out-of-focus frames. Taking that into account, the overall success rate of the whole screening procedure averaged 54%. This rate could be improved by modifying the existing prototype with new materials and upgrading the fabrication procedure, such as implementing hot embossing or additive manufacturing of hard thermoplastics and elastomers^45^. Replacing PDMS with other materials would also prevent the risk of non-specific adsorption of hydrophobic molecules by this polymer^29^. Further upgrade of this platform would involve increasing the number of conditions into a more compact configuration and controlling each reservoir independently, also with the option of injecting predefined dosing profiles. Despite the manufacturing hindrances of the existing prototype, we accomplished a first screening and identified four PTC hits that induced more homogeneous activation of RecA, as a proxy of the SOS response^26^.

To assess the effects of our candidate PTC on the SOS response within clonal mycobacterial cells, we relied on standardized effect-size calculation and their accuracy was estimated with confidence intervals^46^. Mixed-effects models were used to estimate effect sizes on two fluorescence indices. Such model-based approaches are suited to accommodate various technical effects as well as missing values due to out-of-focus images^47^. In order to control technical variation across experiments we advise adding either a passive reference dye such as FITC used in our study, or fluorescent microbeads in all experimental replicates. We considered as biologically relevant all effects equal to at least one-fifth of that caused by the control agent MIT. However, this value is to be determined on a case-by-case basis. The present case resulted in four initial PTC hits, which would have been overlooked by standard selection criteria^11,14,20^. Unlike selections dictated solely by macroscopic population-averaged effects, the key advantage of our µDeSCRiPTor approach is that it allows us to visualize small dynamic changes on individual cells that would be unattainable by static or even dynamic bulk-cell methods^19^. Indeed, μDeSCRiPTor records bacterial microcolonies forming in 2D from a few individuals up to a maximum of a few hundred or at most thousands of cells, thus providing direct spatiotemporal information about their growth parameters and fluorescence during real-time transition between different environmental conditions. This is crucial to access live single-cell transitions, which lie behind cell-to-cell phenotypic variation that cannot be inferred from 3D colonies, as in the case of ScanLag^48^. Furthermore, μDeSCRiPTor allows us to identify subtle differences between compounds clustering in the same functional class that otherwise would not be appreciated. Nevertheless, the reported strategy has a throughput of at least one or two orders of magnitude lower than standard drug-discovery approaches^14,20^, mainly due to manual manufacturing of the platform, long experimental duration, and limited automated analysis, which we are striving to improve by implementing deep neural network segmentation tools^49,50^. Therefore, one possibility is to preselect compounds based on other parameters, without necessarily increasing the likelihood of identifying a PTC. For instance, our candidate PTC included primarily small-molecule compounds with no apparent activity^27^, and a few quinolone analogs^28^, which were the source of our hits. However, for a deeper characterization, we focused on the PTC that had the strongest effect on the SOS indices and was at the same time the most soluble.

Indeed, M06 proved particularly attractive from both a mechanistic and an activity point of view, confirming the soundness of the µDeSCRiPTor approach in identifying compounds that homogenize clonal cells ultimately making them more susceptible to drug-mediated killing. Similarly, it was proposed that noise modulators in HIV^51^ and genetically collapsing phenotypic variation in a tuberculosis model^52^ may improve treatment outcomes. Here we propose that a possible target of our top PTC is NAT, whose expression levels fall upon M06 treatment, and whose genetic modification of the active site and transcriptional inhibition cause M06 resistance. NAT is a ubiquitous enzyme involved in N-acetylation of xenobiotics, causing either substrate inactivation or activation^31,32,34^. However, the main metabolization processes that M06 undergoes in the mycobacterial cell are nitroreduction and methylation, occurring either alone or in combination, inactivating the original compound^28^. This is not surprising since *M. tuberculosis* genome encodes several nitroreductases and methyltransferases, responsible for either prodrug activation or drug inactivation^53,54^. In the case of M06, NAT-mediated N-acetylation could take place only following the reduction of the nitro group with the formation of an arylamine, which is one of the possible substrates of NAT^34^. However, we found that both the intracellular and extracellular levels of acetylated M06 are negligible, ruling out that M06 is a NAT-activated prodrug. Moreover, other N-acetyltransferases in the mycobacterial cell could be responsible for this modification^55^.

NAT was already reported to be a promising anti-tubercular target, as strains devoid of this enzyme show defects in cell-wall mycolates and associated complex lipids, delayed growth, and reduced survival in macrophages^33^. Given the divergence between the human and mycobacterial NAT enzymes, piperidinol analogs, heterocyclic compounds different from our PTC, were formerly identified as potent selective inhibitors of mycobacterial NAT^56^. In support of the assumption that M06 may target NAT, we show that M06 treatment causes a decrease in several cell-envelope components, particularly ketomycolates, which are implicated in cell-wall stability, biofilm formation, and drug tolerance^57^, and accumulation of TMM except in NAT mutant strains. The alterations we observed recall, albeit on a much smaller scale, the inhibition of fatty acid synthase II by INH, which directly targets mycolic acids biosynthesis. Importantly, the reduction in ketomycolates upon M06 exposure was not associated with an accumulation of their precursor hydroxymycolate, which typically hinges on the depletion of the epoxide hydrolase EphD^58^. However, we did find that the expression levels of *ephD* decreased in M06-treated *M. tuberculosis* but not in NAT mutant strains. Overall, we speculate that the broad but moderate inhibitory effect of M06 against mycolic acids and other cell-envelope constituents, which is less pronounced when NAT is mutated or downregulated, is not due to direct inhibition of biosynthetic pathways, but most likely to indirect causes, including perturbation of acetyl-CoA homeostasis following NAT inhibition^33,36^; metabolic remodeling; and oxidative stress. Interestingly, inhibition of cell-wall biosynthesis and cell division was found to trigger the SOS response in *Escherichia coli*^59^. Consistent with this, M06 exposure induced antioxidant defenses, and hindered cell-wall biogenesis, energy metabolism, and respiration, presumably to attenuate the burden derived from oxidative damage^60,61^.

The presence of oxidative stress in *M. tuberculosis* treated with M06 may originate from moderate inhibition of DNA gyrase. Indeed, M06 appears to act as an inefficient quinolone^62^, although it both inhibits the catalytic activity of the enzyme and injures DNA. Interestingly, high concentrations of quinolones were shown to cause a paradoxical effect, whereby higher bacterial survival is associated with lower levels of reactive oxygen species (ROS) and vice versa^63^. We speculate that the weak inhibition of DNA gyrase by M06 may be advantageous for this molecule and lead to lethal levels of ROS, which intoxicate the cell^60,61^. On the other hand, bioreduction of nitroaromatic compounds has been implicated in bacterial cell poisoning^64^. Thus, the nitro group of M06 may also act as a functional electron trap, contributing to the redox toxicity of this compound in the mycobacterial cell, which can carry out several bioreactions on this PTC^53–55^. Deregulation of redox balance was also shown to alter the activity of human NAT, via reversible oxidation of the catalytic cysteine^65^. Since *M. tuberculosis* NAT mutants are less susceptible to M06 and exhibit significantly lower levels of oxidative stress, at present, we cannot exclude that oxidative stress is associated with the anti-mycobacterial activity of M06 and that the mutation found in NAT active site may help the cell to counter this redox imbalance. The presence of two cysteine residues in the active site of NAT could act as redox switches, also helping the cell to preserve the envelope homeostasis^42^. However, if NAT was primarily involved in ROS detoxification or tolerance, its silencing in the presence of M06 should result in increased oxidative-stress burden and greater lipid damage, which we did not observe. Moreover, if the main inhibitory effect of M06 was mediated by ROS, ROS scavengers should help bacteria to better tolerate M06, which was not the case. Taken together, our findings support the hypothesis that NAT inhibition is independent of M06-mediated oxidative stress and that separate mechanisms coexist contributing to M06 potency. Specifically, M06 may jeopardize the mycobacterial cell via NAT inhibition, weakening the cell structure and increasing permeabilization; via DNA gyrase inhibition, increasing DNA lesions; and via ROS production, harming several macromolecules at the same time, and exacerbating cell damage. Based on current evidence, we infer that the hypersensitivity of *M. tuberculosis* to treatment in the presence of M06 arises precisely from its multifactorial mode of action (Fig. 6), which we intend to further investigate in the future, via biochemical and structural analyses, aiming to clarify the role of NAT into M06 pharmacodynamics.

M06 proved to be an interesting compound per se, potent in vitro and with some intracellular activity even at low doses. We were able to show that M06 mediates pheno-tuning both in vitro and in cellulo, in that this PTC induces RecA in clonal populations of *M. tuberculosis* and reduces RecA cell-to-cell variation, and that this phenomenon also takes place within the macrophage. Indeed, we expected RecA induction to be an indicator of cellular impairment in individuals expressing pronounced levels of this broad-spectrum stress marker^22^, which is typically triggered by single-stranded DNA^66^. Therefore, the ultimate goal of our µDeSCRiPTor strategy was to harness this phenomenon to make bacteria more homogeneously susceptible to existing therapy. After testing a sample of anti-tubercular drugs, we could show moderate potentiation of INH by M06. This was expected since NAT, an enzyme that inactivates INH^32,34^, is a putative target of M06. Interestingly, *nat* deletion was also shown to increase the susceptibility of *M. bovis* BCG to some antibiotics, allegedly due to the weakening of the cell wall^33^. Consistent with this, we showed that the combination of M06 with INH increases single-cell lysis compared with INH alone. Weakening of the cell envelope can be justified both by direct inhibition of NAT and by oxidative stress, possibly increasing permeability to various antibiotics. For instance, we showed additivity with MOX that shares its target with M06. In addition, we found that M06 downregulates MfpA, a protein that protects DNA gyrase from quinolones by mimicking DNA^43^. This might explain why clinical isolates resistant to fluoroquinolones remain sensitive to M06. Finally, we found that M06 strongly synergizes with the RNA-polymerase inhibitor RIF, leading to the complete elimination of the persistent fraction in vitro and to intracellular potentiation. This might have different explanations. Steric inhibition of the replication fork by M06 in complex with DNA and DNA gyrase could affect not only DNA replication but also the recruitment of RNA polymerase and initiation of transcription^43,63^. Additionally, since transcription is a major source of negative DNA supercoiling^67^, impairment of this phenomenon by M06 via DNA gyrase inhibition can exacerbate the effect of RIF, with severe impairment of bulk transcription and lethal consequences for the cell. Excessive accumulation of ROS upon M06 exposure could also make bacilli persistent to RIF increasingly vulnerable^10^. Furthermore, concerted impairment of cell-wall lipids, which we observe following both M06 and RIF treatment, could fatally weaken the stability of the mycobacterial cell structure. Lastly, acetylation is a conserved post-translational modification critical in all life domains, although its endogenous roles in bacteria remain largely unknown^68,69^. Interestingly, lysine acetylation of a housekeeping sigma factor was found to promote the activity of the RNA polymerase in actinobacteria^70^. Thus, we cannot exclude that a defect in NAT might have unexpected consequences on transcription as well as on other essential cellular functions.

In conclusion, we developed the µDeSCRiPTor strategy and successfully implemented it with one of the most challenging microbial pathogens, identifying a PTC that potentiates existing anti-tubercular drugs in different in vitro models. PTC-based enhancement of antitubercular drugs may eventually turn out to be effective in shortening treatment duration and preventing disease relapse. Through this work, we also confirm that phenotypic variation is a promising bacterial target, which can suggest original eradication strategies. We expect that this approach will prove useful to identify other compounds that can enhance antimicrobial therapy, helping us to fight infections that are becoming virtually incurable.

## Methods

### Bacterial strains

Cloning and sequencing were carried out in chemically competent *Escherichia coli* TOP10, grown in LB medium in the presence of appropriate selection: 50 µg/mL kanamycin or 100 µg/mL hygromycin. Mycobacteria were cultured in Middlebrook 7H9 broth supplemented with 0.5% BSA, 0.2% glucose, 0.085% NaCl, 0.5% glycerol, and 0.01% Tyloxapol, which was removed for MIC assessment, checkerboard titration, and time-lapse microscopy. Middlebrook 7H10 agar was enriched with 10% OADC and 0.5% glycerol. Mycobacterial transformants and primary cultures were selected on 20 µg/mL kanamycin or 50 µg/mL hygromycin, as appropriate. Bacterial stocks were prepared from single colonies grown up to OD_600nm_ 1.0 in the presence of appropriate selection if needed, supplemented with 15% glycerol, and directly frozen at −80 °C. Each aliquot was used only once to start primary cultures. Primary cultures of *M. smegmatis* mc^2^155 (ATCC 700084), *M. tuberculosis Erdman* (ATCC 35801), and *M. tuberculosis H37Rv* (ATCC 27294) were cultured at 37 °C under shacking conditions, at 150 RPM for the fast-growing strain and 50 RPM for slow-growing pathogenic strains, until reaching mid-log phase. For final assays, secondary cultures were obtained by diluting primary cultures 100 times in an appropriate medium until the mid-log phase was reached.

### Cell lines

RAW 264.7 macrophages (ATCC TIB-71) and Vero cells (ATCC CCL-81) were cultured in Dulbecco’s Modified Eagle Medium (DMEM, Gibco) supplemented with 10% fetal bovine serum (FBS, Gibco) and 1x penicillin-streptomycin mix (PS, Gibco). A stock of about 10^6^ cells per mL in DMEM and 10% DMSO was defrosted at 37 °C, washed once in 10 mL, and inoculated in 30 mL of pre-warmed complete DMEM using a T-175 flask. Cells were propagated at 37 °C in a humidified 5% CO_2_ atmosphere until confluence. For final experiments, confluent cells were diluted to a concentration between 10^5^ and 2.5*10^5^ cells per mL in complete DMEM without antibiotics. THP-1 monocytes (ATCC TIB-202) were cultured in RPMI 1640 (Gibco) supplemented with 10% FBS and 1x PS. A stock of about 10^6^ cells per mL in RPMI and 10% DMSO was defrosted at 37 °C, washed once in 10 mL, and inoculated in 30 mL of pre-warmed complete RPMI using a T-175 flask. Cells were propagated at 37 °C in a humidified 5% CO_2_ atmosphere until confluence, up to four days. For final experiments, confluent cells were diluted to a concentration of 10^5^ cells/mL in complete RPMI without phenol red and without antibiotics and seeded into a 35-mm µ-Dish (ibidi) in the presence of PMA (30 ng/mL). After 36-h incubation, cells were washed with fresh medium and re-incubated overnight before infection.

### Strains construction

The NAT-overexpressing plasmid pGM321 was constructed by PCR amplifying the *rv3566c* open reading frame, preceded by a Shine Dalgarno sequence optimized for mycobacteria and flanked by *Pac*I and *Hpa*I restriction sites, and cloning it inside the L5 integrative plasmid pMV361, under the control of a strong promoter. The anhydrotetracycline (ATC)-inducible dCas9 *nat* knock-down plasmid pGM315 was constructed by inserting a small guide RNA complementary to the *rv3566c* gene in the L5 integrative plasmid pGM309. The RecA translational *M. tuberculosis* reporter strain (GMT37) was constructed by co-subcloning both the *gfp* open reading frame under a strong promoter and the *recA* open reading frame fused in-frame to *mCherry* under its native regulatory region^22^ into a pMV361-based chromosomal integrative vector. Final constructs were confirmed by restriction enzyme profiling prior to electroporation into the recipient strains, and transformants were obtained under appropriate antibiotic selection. Bacterial strains and plasmids (Supplementary Table 1), and oligonucleotides (Supplementary Table 2) are provided in the Supplementary Information.

### Bulk growth assay

*M. tuberculosis* primary cultures were grown up to OD_600nm_ 0.5–0.6 and diluted to OD_600nm_ 0.025 or 0.05 using pre-warmed Middlebrook 7H9 broth. Growth kinetics were assessed by measuring the OD_600nm_ over 24 h for *M. smegmatis* and between 1 and 2 weeks for *M. tuberculosis*.

### Chemistry

The PTC candidates used in this study are listed in Supplementary Data 2. Fragment compounds were obtained following the deconvolution of the 6 best hits from a curated small-molecule fragment library of 1604 fragments, combined into 169 pools, which were formerly tested on *M. smegmatis* and *M. tuberculosis*^27^. The 65 singletons derived from this primary screening were purchased from Key Organics UK and showed low inhibition against both mycobacterial species, based on MIC evaluation. The 7 phenanthroline derivatives were selected from a library of 28 compounds synthesized at CERMN^28^ and based on their antimycobacterial activity. See also Supplementary Methods.

### Structure and operation of the 32-condition microfluidic platform

The multi-condition platform derives from a scalable microfluidic module we developed earlier^25^. This module, also referred to as microchamber, is composed of two layers of PDMS patterned with a 300µm-wide channel crossing a 1000µm-wide circular chamber. The two layers are bonded perpendicularly to each other and in turn to a glass coverslip, creating two functional compartments: an upper control layer, where water is perfused; and a lower flow layer, where bacterial suspension and culture medium are perfused (Supplementary Fig. 2a, b). The upper section of the flow layer constitutes the third functional element of the module, namely, a 20µm-high flexible membrane on top of the circular chamber. By increasing pressure in the control layer, the membrane pneumatically lowers, forming a contact zone with the underlying coverslip, where bacteria are confined and forced to replicate in two dimensions (Supplementary Fig. 2c).

The flow layer is made of an array of 16 × 2 pairs of microchambers overlaid by a control layer, whose microchannels are arranged perpendicular to the microchannels of the flow layer (Supplementary Fig. 1a; Supplementary Fig. 2d). The microchambers are fed from a single inlet port through a unique microfluidic network, which separates into 16 individual branches per side. Each branch alternates straight microchannels and microserpentines of diverse lengths, to ensure that the stream flows into the different microchambers at the same time. Each branch is also independently connected to a second separate inlet path, which in turn is fed from an individual reservoir. The reservoirs are made of two perforated metal blocks (16 holes per block) placed on top of each side of the device (Supplementary Fig. 2e) and are controlled by air injection via two sealed lids (Supplementary Fig. 2f). The perforated metal blocks are interconnected to 32 inlet ports, flowing into each branch of the flow layer toward a single pair of microchambers. All microchambers are connected to an output channel that flows into a common outlet port, toward a sealed waste container. The whole microfluidic platform is regulated from a software-driven flow controller, via four channels (Supplementary Fig. 1a). The first channel actuates the flow layer by applying a pressure of 100 mbar to a bottle of medium. The second channel actuates the control layer, which is clamped at one end, by applying a pressure of 30 mbar to a water supply, resulting in lowering the membrane on the underlying coverslip. The third channel is connected to the two arrays of reservoirs and is set at 30 mbar. The fourth channel is connected to the waste container and is set at 20 mbar.

### Microfabrication of the 32-condition microfluidic platform

The designs of the flow and control layer were generated on AutoCAD 2017 (Supplementary Data 1), and corresponding low-resolution plastic photomasks were printed at Selba S.A. The micropatterns were transferred from the photomasks to 100-mm silicon wafers by photolithography, producing one wafer for the flow layer (FL-W) and a second wafer for the control layer (CL-W).

Two silicon wafers were dehydrated at 200 °C on a hot plate for 30 min. The FL-W was produced by spin-coating SU8-2025 photoresist at 3000 RPM for 30 s, to generate a layer of 30 µm, and soft-baking at 65 °C for 1 min, followed by incubation at 95 °C for 5.5 min. The photoresist was crosslinked under 155 mJ/cm^2^ UV light for 11.5 s, and the wafer was baked at 65 °C for 1 min, followed by 95 °C for 5 min. Finally, the FL-W was developed by immersion into propylene glycol methyl ether acetate (PGMEA) for 4 min and washed extensively with isopropanol. The CL-W was generated by spin-coating the SU8-2100 photoresist at 1500 RPM for 30 s, to generate a layer of 200 µm, and soft-baking at 65 °C for 5 min, followed by 95 °C for 20 min. The photoresist was crosslinked under 240 mJ/cm^2^ UV light for 21.8 s, and the wafer baked at 65 °C for 5 min, followed by 95 °C for 10 min. Finally, the CL-W was developed by immersion into PGMEA for 15 min and washed extensively with isopropanol. The FL-W and CL-W were further hard-baked at 180 °C for 2 h, and silanized with Trichloro (1H,1H,2H,2H-perfluorooctyl) silane in a vacuum chamber overnight. The microchannel height was profiled with a DektakXT (Bruker).

For soft-lithography, the FL-W was covered with a degassed mixture of 20 g of silicone elastomer SYLGARD 184 (Dow Corning) and 1 g of crosslinking agent, spin-coated at 1500 RPM for 60 s, aiming to obtain a PDMS layer of 50 µm. The PDMS-coated FL-W was incubated at room temperature for 20 min and baked at 80 °C for 18 min. The CL-W was put in a square Petri dish and covered with a degassed mixture of 40 g of silicone elastomer and 8 g of crosslinking agent, degassed for 30 min in a vacuum chamber, and baked at 60 °C for 30 min. Patterned PDMS was cut with a blade and detached from the mold. Inlet ports were generated via a biopsy punch (1.0 mm OD, Harris Uni-Core). The CL was manually aligned with the FL, by visually overlapping the microchambers of the two layers. Bonding between the two layers was ensured by the presence of different amounts of crosslinking agent, which migrated from the CL to the FL. Next, metallic connectors (0.8/1.2 mm ID/OD, Phymep) were inserted into the inlet and outlet ports of the CL. To consolidate the assembly, the bonded FL and CL were further covered with a degassed mixture of 30 g of silicone elastomer and 3 g of crosslinking agent, further degassed in a vacuum chamber for 30 min, and baked at 80 °C overnight. PDMS was detached from the FL-W, metallic connectors were removed, and the inlet and outlet ports of the FL were punched (1.0 mm OD, Harris Uni-Core).

Next, a large coverslip (112 × 100 × 0.2 mm) was cleaned with isopropanol, dried, and bonded to the PDMS assembly by oxygen plasma treatment. Specifically, the coverslip was put inside a plasma chamber, the vacuum was created at 0.1 mbar, and oxygen was inflated at 0.25 mbar for 60 s. Afterward, the PDMS assembly was put inside the plasma chamber with the microchannels upward. The same cycle was run for a duration of 30 s. Just after plasma activation, the PDMS assembly was placed on the coverslip, covered with a metal weight (130 × 100 × 30 mm) of 500 g, and incubated at 80 °C overnight to complete bonding.

To fix the metallic reservoirs (Supplementary Fig. 2e) on the microfluidic device, 5 g of silicone elastomer and 0.5 g of crosslinking agent were mixed and degassed and poured on the PDMS assembly using a 2-mL syringe, after closing the reservoir ports with metallic connectors. Next, the metallic reservoirs were placed on the surface of the device, and PDMS was crosslinked at room temperature for 24 h. Following this first incubation step, the metallic connectors were removed, and the half-kilo metal weight was placed on top of the assembly, which was further incubated at 80 °C overnight. Finally, the platform was decontaminated in a UVO-Cleaner (Jelight) for 20 min and stored in a sterile petri dish.

A pair of acrylic lids were manufactured, to seal each reservoir and inflate air, for injecting the contents of the reservoirs into the device microchambers. Each injector was made by gluing two 2-mm acrylic rectangles patterned with a groove and rimming thermoplastic polyurethane tape around the perimeter of the lid (Supplementary Fig. 2f). Finally, a metal connector, plugged into a 7-cm-long Tygon tubing with a female Luer lock at the opposite end, was also glued within the groove of the lid. The tubing was then connected to the third channel of the flow controller to drive injection. The metal and acrylic holders (Supplementary Fig. 2g, h) were also manufactured.

### Characterization of mycobacterial growth in the 32-condition microfluidic platform

Single-cell suspensions of *Mycobacterium smegmatis* wild type or of the dual-fluorescent-reporter RecA-GFP_mCherry_cyt_ (GMS2_pGM218) were manually loaded from the outlet port, which was then connected to the waste container. Following 10-min incubation, both the control layer and the flow layer were actuated to block and feed bacterial cells, respectively. The flow rate was monitored by two flowmeters installed upstream and downstream of the flow layer, and adjusted by ±15 mbar, aiming to obtain a targeted flow rate in the flow layer of 160 µL/h at the inlet and of 150 µL/h at the outlet. Reservoirs were not used in these experiments. The microfluidic device was mounted on the microscope stage, using a metallic and acrylic support system, held together by screws, and having a series of openings to allow insertion of tubing and other components from the top and movement of the objective from the bottom (Supplementary Fig. 2g−i). Time-resolved microscopy was carried out on an inverted DeltaVision Elite Microscope (Leica), equipped with a UPLFLN100XO2/PH3/1.30 oil objective (Olympus). Exposure conditions: phase contrast 100% T, 150 ms; FITC (Ex 475/28, Em 525/48 nm) 50% T, 150 ms; mCherry (Ex 575/25, Em 625/45 nm) 50% T, 150 ms. Images were acquired at 30-min intervals. A maximum of 10 fields of view were recorded per microchamber. Each experiment was repeated twice. The device was discarded after use following decontamination with 2.6% sodium hypochlorite. Flowmeters were washed with 2.6% sodium hypochlorite, 70% ethanol, and sterile water for 1 h, and all the connection accessories were autoclaved.

### Characterization of injection from the reservoirs

Qualitative validation was carried out with food dyes (Supplementary Fig. 2i). The platform was first manually filled with 7H9 medium from the outlet until a drop of the medium was visible at the inlet ports of the reservoirs. Next, the reservoirs were alternately filled with red and blue food dyes and closed by screwing the injection lids. The inlet port of the FL was connected to the medium bottle that was closed with a pressure lid (Fluigent) and flangeless fittings (IDEX Health & Science), which were connected to a Tygon tubing (Saint-Gobain), in turn connected to a metallic fitting on the other end. The outlet port was connected to an empty waste bottle in the same way. The inlet port of the CL was connected to a 50-cm Tygon tubing via a metallic connector, and the other end of the tubing was connected to the flangeless fitting of the water reservoir. The outlet port of the CL was connected to a 5-cm Tygon via a metallic fitting and locked with a plier. The platform was controlled from the multi-channel Microfluidic Flow Control System (MFCS, Fluigent), via the MAESFLOW Control software (Fluigent): channel (C) 1 drove the FL inlet; C2 drove the CL inlet; C3 drove the reservoirs injecting lids; and C4 drove the FL outlet. First, the FL was perfused with 7H9 medium by setting C1 at 200 mbar and closing the outlet with a plier for 20 min to degas the FL fluidic network. Next, C1 was set at 100 mbar, C2 and C3 were set at 30 mbar, and C4 at 20 mbar for 10 min. To actuate the reservoirs, C1 was set at 60 mbar and C3 at 100 mbar for 30 min, after which the device was photographed.

Quantitative validation was carried out with fluorescein isothiocyanate (FITC) (Supplementary Fig. 1e; Supplementary Fig. 2j−l). The device was pre-filled with 7H9 medium and connected as described above, and the reservoirs were alternately filled with a FITC solution (100 µM) and with non-fluorescent 7H9 medium. The platform was mounted on the microscope stage via the custom-made holders. The FL was perfused with 7H9 medium by setting C1 at 200 mbar for 20 min and closing the outlet with a plier for degassing. Next, C1 was set at 100 mbar, C2 and C3 were set at 30 mbar, and C4 at 20 mbar. Pressures were further adjusted by ±15 mbar, aiming to achieve a flow rate of 160 µL/h at the FL inlet port and 150 µL/h at the FL outlet port. After 6 h, C1 was set at 60 mbar and C3 was set at 100 mbar, to start injection from the reservoirs for 6 h. To stop injection from the reservoirs and restore the main flow from the bottle of the medium, C1 was reverted to 100 mbar, C2 to 35 mbar, and C3 to 30 mbar. At the end of the injection sequence, the whole device was manually filled with 100-µM FITC solution, to image the fluorescence of undiluted dye. To determine the dilution that the solutions stored in the reservoirs undergo during injection, via mixing with the medium in the flow layer, fluorescence signals were measured in the channel section just preceding the microchambers during injection and compared to the signal obtained from undiluted FITC solution. We calculated a dilution of approximately 2.5 times. Fluorescence images were acquired on a DeltaVision Elite Microscope, equipped with a Plan-Apochromat 10x/0.45 M27 objective (Zeiss), using the FITC channel (Ex 475/28, Em 525/48 nm) at 50%T, for 100 ms.

### Dynamic microfluidic screening setup and imaging

All connecting fluidic material was autoclaved before use, and device preparation was carried out under a biosafety cabinet. The inlet port of the FL was connected to 50-cm Tygon tubing via metallic fitting. The other side of the tubing was attached to the 7H9 medium bottle as described previously, also adding a flowmeter M size (Fluigent) in between. The inlet port of the CL was connected to a 50-cm Tygon tubing via metallic fitting, and the other end of the tubing was attached to the water reservoir as described previously. The outlet port of the CL was connected to a 5-cm Tygon via metallic fitting and closed with a plier.

The outlet port of the FL was first used to manually seed the bacterial single-cell suspension, obtained from filtration through a 5-μm filter (Merck), and then to drain the spent medium into the waste bottle, filled up for one-fifth with a 2.6% sodium hypochlorite solution. About 400 µL were gently injected from a 1-mL syringe connected to a 5-cm Tygon tubing (1.2 mm ID, Saint-Gobain) via a female Luer-to-Barb fitting, and the opposite side of the tubing was connected to the FL outlet via metallic fitting. Following 10-min incubation at room temperature, the FL outlet was connected to a 50-cm Tygon tubing via metallic fitting. A flowmeter was connected to this outlet tubing, and the other end was connected to a 5-cm Tygon tubing with a flangeless fitting plugged into a waste bottle as described previously. Next, the reservoirs were filled with 300 µL of compounds to be screened, at a concentration 2.5 times higher than the final concentration to be tested, due to the dilution occurring with 7H9 medium, flowing in the main microchannel network, during injection. Compounds with a MIC > 500 μM were tested at a final concentration of 250 μM; compounds with a MIC ≤ 500 μM were used at a final concentration equal to 0.5-fold MIC. DMSO was used as a negative control; MIT was used as a positive control at a final concentration of 300 nM. To control injection, the two conditions at the extremities of the reservoirs were filled with FITC solution (100 µM). After filling, the reservoirs were sealed by screwing on the injection lids.

Finally, the loaded platform was mounted on the microscope stage via the custom-made holders and controlled via the MFCS (Fluigent) as described above: C1 drove the FL inlet; C2 drove the CL inlet; C3 drove the reservoirs injecting lids; and C4 drove the FL outlet. The FL was first perfused with 7H9 medium at 200 mbar for 20 min, and the FL outlet was closed with a plier for another 20 min for degassing the microfluidic network. Next, the plier was removed and C1 was set at 100 mbar, C2 and C3 at 30 mbar, and C4 at 20 mbar, with further adjustments of ±15 mbar, targeting a flow rate of 160 µL/h at the FL inlet port and 150 µL/h at the FL outlet port. After the pre-growth stage, compounds were injected by setting C1 at 60 mbar and C3 at 100 mbar for 6 h, and injection was stopped by reverting C1 to 100 mbar, C2 to 35 mbar, and C3 to 30 mbar.

Time-resolved microscopy was carried out on an inverted DeltaVision Elite Microscope (Leica), equipped with a UPLFLN100XO2/PH3/1.30 oil objective (Olympus). Exposure conditions: phase contrast 100% T, 150 ms; FITC (Ex 475/28, Em 525/48 nm) 50% T, 150 ms; mCherry (Ex 575/25, Em 625/45 nm) 50% T, 150 ms. Images were acquired at either 20- or 30-min intervals. In each microchamber between 5 and 10 fields of view were recorded, each one containing between 1 and 10 individual bacteria. Each experiment was performed at least twice independently. At the end of each experiment, the platform was decontaminated with 2.6% sodium hypochlorite and discarded; the two flowmeters were washed with 2.6% sodium hypochlorite, followed by 70% ethanol and sterile water for 1 h; tubing, fittings, and connectors were autoclaved.

### Time-resolved image analysis and effect size estimation

Movies derived from the dynamic microfluidic screening were analyzed at the microcolony level for all compounds and at the single-cell level for shortlisted compounds.

#### Data extraction for microcolonies

Raw image stacks were separated by channel and smoothed, applying a Gaussian convolution kernel. Whole-microcolony binary masks were created based on the background of red channel images, using K-means clustering to separate the regions of interest (ROI), containing cells, from the remaining background image. All objects in the image that were smaller than a single cell, detached from the main ROI, or located on the edge of the image were excluded from the final mask. Further, a shape complexity index (${Perimeter}/\surd {Area}$) was calculated for each ROI. To do so, a Sobel operator was applied to identify boundaries between microcolony and background. Only images with a shape complexity index higher than 14 (determined based on a subset of images) were kept. This process was repeated iteratively for each image in the stack (movie), and the final binary masks were visually inspected. Images were retained with an overall success rate of 54%. The growth rate of microcolonies was estimated based on size increase over time using a robust regression, assuming growth was exponential. Pixel intensities within the ROI were centered based on the average value of the image background and log_10_ transformed to reduce the effects of background noise. ROI was also used to estimate the mean (µ_RecA-GFP_) and variance (σ^2^_RecA-GFP_) of fluorescence intensity. The latter was used as a final measure of cell-to-cell variation. The average of each index was computed in every colony across the four last time points of each stage, the pre-exposure and the exposure stage. Each microcolony dataset included: three variables of interest (fluorescence mean; fluorescence variance; and growth rate); two experimental factors (compound and exposure stage, pre- or during exposure); and two technical factors (experiment replicate and microchamber coordinates).

#### Data extraction for single cells

*M. smegmatis* single-cell analysis was carried out on ImageJ (2.0.0-rc-59/1.51n)^71^, using ROI Manager macro at given time points. Individual cells were manually segmented using the selection brush tool, and background mean fluorescence intensity was subtracted from each ROI to obtain final single-cell µ_RecA-GFP_ intensities. As done for microcolonies, average and variance of fluorescence intensity were also calculated for single cells. Finally, each single-cell dataset included: two variables (fluorescence mean and fluorescence variance); two experimental factors (compound and exposure stage); and two technical factors (experimental replicate and microchamber coordinates).

#### Statistical analysis

Mixed-effects models were used to assess the effect of exposure to PTC. The experimental factors (compound and exposure stage) were set as fixed effects, whereas technical factors were set as random effects. Standardized effect sizes were calculated to assess the impact of exposure to PTC as compared to the negative control group (DMSO). The statistical significance of these effects was tested based on post hoc comparisons. Large positive or negative effect sizes reflect a strong increase or decrease, respectively of values upon PTC exposure as compared to DMSO. Data extraction and analysis were performed with the R software 4.0.2 (https://www.r-project.org/).

### In silico docking

Docking poses of M06 or its amino derivative, INH and MOX were generated on SeamDock docking web server (https://bioserv.rpbs.univ-paris-diderot.fr/services/SeamDock/)^72^. The structure PDB 4BGF of the arylamine N-acetyltransferase from *M. tuberculosis* cross-seeded with the crystals of *Mycobacterium marinum* protein^36^ and the structure PDB 5BS8 of the DNA gyrase from *M. tuberculosis*^30^ were used to predict ligand binding. Either Vina or Autodock was used to assess protein-ligand interactions with the following settings: grid spacing between 0.2 and 1 Å; energy range at 5 kcal/mol; and exhaustiveness at 32.

### MIC evaluation by resazurin assay

Secondary cultures were grown up to OD_600nm_ 0.6–0.8, diluted to OD_600nm_ 0.005 using pre-warmed Middlebrook 7H9 broth, and 100 μL were dispensed into a 96-well plate, including negative and positive controls. ATC (100 ng/mL) was added during the assay to induce silencing of *nat*. Wells containing the highest drug concentration to be tested were filled with 200 µL of cell suspension and used for sequential two-fold dilutions. Plates were incubated under static conditions for 7 days at 37 °C, after which 10 µL of resazurin (0.01%) were added to each well. Plates were further incubated for 24 h and then visually inspected, considering blue as cidality and pink as viability. MIC was regarded as the lowest concentration of drug causing cidality.

### Checkerboard assay

The efficacy of dual drug-combinations was tested by checkerboard titration methods using 96-well plates, as described for the MIC assay. The first top-left well was used for the negative control. The rest of the first row and the first column were used to dilute each compound alone, whereas the highest concentrations of the two compounds to be tested (A and B) were alternately placed in the second row and in the second column, to test crossed dilutions. The drug concentrations tested ranged from 30 to 0.03 mM, except for M04, which ranged from 300 to 0.3 µM. The fractional inhibitory concentration (FIC) index for combinations of two compounds A and B was calculated according to the following equation: FIC index = (A/MIC_A_) + (B/MIC_B_), where A and B are the MIC of compound A and compound B in combination, and MIC_A_ and MIC_B_ are the MIC of compound A and compound B alone. The FIC index was interpreted as follows: synergy (≤0.5), additivity (0.5 < FIC ≤ 1), and indifference (>1).

### ATP cell viability assay

The effect of different ROS scavengers on M06 potency was assessed by crossing increasing concentrations of M06 and of a given ROS scavenger and checking its effect on bacterial viability compared with M06 alone. The following ROS scavengers were tested: 2,2′-bipyridyl; N,N’-dimethylthiourea; glutathione; N-acetyl cysteine; catalase. The same method was used to assess the effect of B02 and F05 in combination with different anti-tubercular drugs. Mid-log phase *M. tuberculosis* cultures were diluted to OD_600nm_ 0.005 and incubated in the presence or absence of different concentrations of individual compounds or their combinations for one week. Cell viability was determined by luminescence to estimate ATP production using the BacTiter-Glo Microbial Cell Viability Assay on a GloMax Discovery System endowed with the GloMax Discover software (Promega), according to the manufacturer’s instructions. *M. tuberculosis* viability was expressed as relative luminescence units of ATP (RLU) in the presence of treatment versus untreated cells.

### Kinetic assay of drug-mediated mortality

Secondary cultures of *M. tuberculosis* H37Rv were grown up to OD_600nm_ 0.5 and diluted to OD_600nm_ 0.05 (10^6^ to 10^7^ bacilli) in fresh 7H9 medium containing 0.01% Tyloxapol. Drugs were added as follows: INH (MIC: 50 ng/mL, 2x MIC); RIF (156 ng/mL, 2x MIC); MOX (100 ng/mL, 2x MIC); EMB (5 µg/mL, 2x MIC); M06 (1.25 µg/mL, 1x MIC); or their combinations. Drugs were added at the beginning of the assay and never replenished. Untreated and drug-treated cultures were re-incubated at 37 °C at 50 RPM and aliquots were withdrawn sequentially for plating different dilutions on 7H11 agar medium enriched with 10% OADC and 0.5% glycerol. CFU were quantified after 4 weeks of incubation at 37 °C and normalized to 10^7^ at time zero.

### Toxicity assay

Confluent Vero cells were dissociated by trypsinization, washed, and resuspended in complete DMEM medium at a concentration of 2.5*10^5^ cells/mL, and 100 µL were dispensed into a 96-well plate and incubated for 24 h at 37 °C in humidified 5% CO_2_ atmosphere. Each compound (except for A1, A2, A3, A5, C2, and D10) was tested at a maximum concentration of 8 mM or 0.5 mM, depending on reagent availability, and serially diluted. Cells were exposed to the compounds for 24 and 48 h. Survival was assessed by ATP luminescent cell viability assay (CellTiter-Glo®, Promega), using a Biohit Microplate Reader and integrated software. Half maximal inhibitory concentration (IC50) was estimated by nonlinear regression analysis of compound concentration versus normalized response using GraphPad Prism software.

### Isolation of PTC-resistant mutants

Mutants were isolated in two steps. Secondary cultures of *M. tuberculosis* H37Rv were grown up to OD_600nm_ 1. About 10^9^ cells were harvested and spread on 7H10 agar plates containing drug concentrations ranging from 4- to 20-fold MIC, and plates were incubated at 37 °C for 4 weeks. The only colonies obtained at 4-fold MIC were first inoculated into a liquid medium containing the same drug concentration, then re-inoculated at twice the concentration, and finally seeded on plates containing a drug concentration equal to 8-fold MIC. The resistance phenotype was validated by assessing the MIC at least twice for isolated colonies. The mutant frequency was about 5*10E-8.

### Genomic DNA isolation and whole-genome sequencing

PTC-resistant mutants were reinoculated in the presence of the compound (8x MIC) and 10 mL of exponentially growing cultures were used for genomic DNA isolation, as follows. Each cell pellet was heat-inactivated at 80 °C for 1 h. Inactivated cells were washed and resuspended in 450 µL of lysis solution (25 mM Tris-HCl pH 7.9; 10 mM EDTA 50 mM glucose), also adding 50 µL of lysozyme (10 mg/mL) and incubated at 37 °C overnight. Next, 100 µL of sodium dodecyl sulfate (10 %) and 50 µL of proteinase K (10 mg/mL) were added, mixed gently by inversion, and incubated at 55 °C for 30 min. Next, 200 µL of sodium chloride (5 M) was added and mixed gently by inversion, followed by 160 µL of preheated cetrimide saline solution (4 %), mixed and incubated at 65 °C for 10 min. Genomic DNA was extracted twice, adding equal volume (1 mL) of chloroform: isoamyl alcohol (24:1), mixing by inversion, and centrifuging for 5 min at 13500 *g*. The aqueous layer was transferred to a clean tube (800 µL) and 560 µL (0.7 volumes) of isopropanol was added at room temperature, and mixed by inversion until DNA precipitation. DNA was collected by centrifugation for 10 min and the pellet was washed with 70 % ethanol, dried, and resuspended in 100 µL of TE buffer. Libraries for whole-genome sequencing (WGS) were built using a TruSeq DNA PCR-Free Low Throughput Library Prep Kit (Illumina, USA) following the manufacturer’s protocol. Quality control was performed on Agilent Bioanalyzer. DNA sequencing was performed on the Illumina MiSeq 250 platform using paired-end 150-bp reads.

### Variant calling

Variant calling analysis was performed with Sequana 0.9.8^73^, using the variant-calling pipeline 0.10.0 built on top of Snakemake 6.1.1^74^. Briefly, whole-genome sequencing reads were aligned to the reference genome of *M. tuberculosis* H37Rv v2 using bwa 0.7.17^75^. Sambamba 0.8.0^76^ was used to mark and filter duplicated reads. Variants were identified using Freebayes 1.3.2^77^ and annotated using SNPeff 5.0^78^. High-quality single-nucleotide polymorphisms (SNP) were selected using the following criterions: freebayesscore: 20; frequency: 0.7; mindepth: 10; forwarddepth: 3, reversedepth: 3, strandratio: 0.2. Quality control statistics were summarized using multiQC 1.10.1^79^.

### Total RNA extraction and sequencing

Secondary *M. tuberculosis* Erdman cultures were grown in Middlebrook 7H9 broth until the exponential growth-phase (OD_600nm_ 0.6) and were subjected to different compounds at a concentration of 10-fold the MIC for 6 h, in triplicate. DMSO was used as a negative control. For total RNA isolation, 10 mL of each culture were collected at 4200 x *g* for 15 min at 4 °C and frozen at −80 °C for 24 h. RNA was extracted by hot-phenol method and precipitated with 0.5 M LiCl and three volumes of cold 100% ethanol for 2 h at -20 °C. Samples were centrifuged at 13500 x *g* for 35 min at 4 °C and RNA was washed with 80% ethanol. RNA samples were air-dried for 30 min and resuspended in 20 µL of nuclease-free water. DNA was depleted by double DNase treatment, using the TURBO DNA-free kit (Ambion). The quality and integrity of RNA samples were checked on Agilent 2100 Bioanalyzer, and the amount was quantified on the Qubit fluorimeter (Thermo Fisher). Ribosomal RNA was depleted using the QIAseq FastSelect 5 S/16 S/23 S kit for bacteria (Qiagen), according to the manufacturer’s recommendations. Libraries were prepared using the TruSeq Stranded mRNA Library Preparation kit (Illumina) according to manufacturer’s protocol: (https://support.illumina.com/content/dam/illumina-support/documents/documentation/chemistry_documentation/samplepreps_truseq/truseq-stranded-mrna-workflow/truseq-stranded-mrna-workflow-checklist-1000000040600-00.pdf). The quality, integrity and amount of the libraries were checked with the Agilent 2100 Bioanalyzer and Qubit fluorimeter. Lastly, single-end sequencing was carried out on a NextSeq500 mid-output system (Illumina), 130 M reads.

### RNA-seq analysis

RNA-seq datasets were produced from three independent experimental replicates. Sequana 0.9.8 rnaseq pipeline 0.10.0 built on top of Snakemake 5.8.1 was used to perform data analysis. In short, reads were trimmed from adapters using Cutadapt 2.10^80^, then mapped to *M. tuberculosis* Erdman genome (ASM35020v1) using bowtie 2.2.2^81^. FeatureCounts 2.0.0^82^ was used to produce the count matrix, assigning reads to features with strand-specific information. Quality control statistics were summarized using MultiQC 1.8. Statistical analysis on the count matrix was performed to identify DEGs, comparing *M. tuberculosis* treated with a given drug relative to the DMSO control condition. The clustering of transcriptomic profiles was assessed using a Principal Component Analysis. Differential expression testing was conducted with the Wald test using DESeq2 library 1.24.0^83^ with SARTools 1.7.0^84^, indicating the significance (Benjamini–Hochberg adjusted *P*-values, false positive rate <0.05) and the effect size (fold-change) for each comparison. GO enrichment analysis was carried out using the Gene Ontology Resource powered by PANTHER (http://geneontology.org/)^85,86^.

### Real-time quantitative PCR

To validate transcriptional silencing of *nat* by CRISPRi, secondary cultures of GMT36 and related control were grown up to OD_600nm_ 0.6–0.8, diluted to OD600 0.05, and induced with 100 ng/mL of ATC for 4 days in triplicate. To quantify the expression of genes implicated in stress response, *M. tuberculosis* H37Rv and NAT mutants (S9, S10, and S11) were cultured in Middlebrook 7H9 broth until OD_600_ 0.6, in triplicate. Next, cultures were exposed to M06 (10x MIC) for 6 h. Total RNA was extracted by hot-phenol method and DNA was depleted, as described above. cDNA was generated from 150–200 ng of total RNA using SuperScrip IV (Invitrogen) and random hexamers (Thermo Fisher), according to the manufacturer’s instructions. qRT-PCR analysis was carried out using the SYBR Green PCR Master Mix (Applied Biosystems), with 0.3 μM primers, and 1 μL cDNA diluted 1:4. Absolute quantification was run on the LightCycler®480 Instrument (Roche Life Science): activation at 50 °C for 2 min (ramp rate °C/s 4.8) followed by 95 °C for 10 min; 40 amplification steps at 95 °C for 15 s (ramp rate °C/s 4.8), followed by 60 °C for 30 s (ramp rate °C/s 2.5) and 72 °C for 30 s (ramp rate °C/s 4.8); melting curve at 60 °C for 15 s (ramp rate °C/s 2.5), followed by 95 °C for 15 s (ramp rate °C/s 0.29). The genomic DNA of *M. tuberculosis* was serially diluted to generate standard curves and calculate transcript copy numbers.

### Snapshot microscopy and single-cell image analysis

Secondary cultures of *M. tuberculosis* H37Rv, NAT mutant, and NAT-overexpressing strains were cultured in Middlebrook 7H9 broth until OD_600_ 0.5, in duplicate. Secondary cultures of *M. tuberculosis* GMT36 and control strain were diluted to OD_600_ 0.05 and induced with ATC (100 ng/mL) for 3 days prior to stress exposure. Mid-log phase secondary cultures were treated for 24 h with DMSO (CT), H_2_O_2_ (30 mM), or with different drugs at 10x-MIC concentration: M06 (12.5 µg/mL); MOX (500 ng/mL); INH (250 ng/mL); RIF (800 ng/mL). Next, bacilli were stained with either CellROX (5 µM) or FM464 (5 µg/mL) dyes for 30 min and imaged by phase contrast and fluorescence using an inverted DeltaVision Elite Microscope (Leica) equipped with an UPLFLN100XO2/PH3/1.30 objective (Olympus). Samples for imaging were prepared by dispensing 0.6 μL of bacterial suspensions between two #1.5 coverslips, sealed with glue. Exposure conditions: phase contrast 50% T, 100 ms; Cy5 (Ex 632/22, Em 679/34) 50% T, 100 ms for CellROX detection; TRITC (Ex 542/27, Em 597/45) 50% T, 100 ms for FM464 detection. Individual bacilli were automatically segmented in phase-contrast, using Omnipose^49^ for cell segmentation in combination with a custom-trained model. The latter was obtained from training on a dataset consisting of the ground truth dataset provided as part of Omnipose and additional manually labeled images of *M. tuberculosis*. A Python notebook was written to extract single-cell fluorescence and cell area from snapshot images using the Omnipose-derived segmentation masks. Inverted masks were used to extract background fluorescence, which was subtracted from the fluorescence of each cell.

### Time-lapse microscopy and single-cell image analysis

Log-phase *M. tuberculosis* RecA-mCherry_GFP_cyt_ reporter (OD_600nm_ 0.5) was diluted ten times in fresh 7H9 medium in the absence of detergents and seeded into our microfluidic hexa-device^22^. Three independent cultures were loaded on different areas of the device. The microfluidic assembly was mounted on a DeltaVision Microscope at 37 °C. Growth medium in the absence or presence of M06 (1.25 µg/mL, 1x MIC) and/or INH (0.2 µg/mL, 8x MIC) was injected at 10-µL/min rate from a syringe pump, according to the different experimental phases. Images were acquired every 3 h using a UPLFLN100XO2/PH3/1.30 objective (Olympus) and a high-speed sCMOS camera, 2560 ×2160 pixels, pixel size 6.5 × 6.5 μm, 15-bit, the spectral range of 370–1100 nm. Exposure conditions were phase contrast 100% T, 200 ms, FITC (Ex 492/28, Em 523/23) 50% T, 50 ms; TRITC (Ex 556/25, Em 611/47) 100% T, 250 ms. An endpoint permeabilization assay was carried out by staining bacilli with DRAQ7 (1 µM) for 6 h, and imaging on CY-5 (Ex 632/22, Em 679/34) 100% T, 300 ms. Individual cells were manually segmented at specific time points using ROI Manager Macro of ImageJ 2.0.0.-rc-59/1.51n or counted over time using the Cell Counter plugin. Total fluorescence was normalized to cell area and background fluorescence was also subtracted.

### Intracellular RecA induction

Differentiated THP-1 macrophages were infected with *M. tuberculosis* RecA-mCherry_GFP_cyt_ reporter (GMT37) diluted in RPMI without phenol red to OD_600_ 0.005 (MOI 1:5). After 3 h of incubation at 37 °C in humidified 5% CO_2_ atmosphere, cells were washed five times with complete RPMI without phenol red. Infected cells were either left untreated or were treated with M06 (1.25 µg/mL, 1x MIC). Imaging was carried out at time zero and after 2 days of infection. Eight images were acquired for each field of view through a z-stack of 16 µm, starting the scan from the middle of the sample, using a 100x oil immersion objective, 1.4NA, WD 0.12 mm (Olympus). Exposure conditions on the Delta Vision microscope: bright field 10% T, 50 ms; FITC (Ex 475/28, Em 525/48) 50% T, 25 ms; TRITC (Ex 542/27, Em 597/45) 100% T, 100 ms. Image stacks were projected by maximum-intensity in SoftWorx 7.0 Cytiva, and processed using a customized ImageJ macro, which automatically segments intracellular bacterial foci based on their green fluorescence. Derived masks were used to measure the size and both fluorescence channels to assess RecA induction and control GFP_cyt_ fluorescence of intracellular bacilli. Inverted masks were used to subtract background fluorescence from each ROI.

### DNA supercoiling and cleavage assays

To determine the inhibition of negative supercoiling on 0.5 µg of relaxed pBR322 (Inspiralis), we used 1.2 U of DNA gyrase from *M. tuberculosis* (Inspiralis). The reactions were carried out in a final volume of 30 µL, using the following reaction buffer: 50 mM HEPES KOH pH 7.9; 6 mM magnesium acetate; 4 mM DTT; 1 mM ATP; 100 mM potassium glutamate; 2 mM spermidine; 0.05 mg/mL albumin, in the presence of DMSO or of different concentrations of MOX and M06. After addition of DNA gyrase, the reaction was incubated for 75 min at 37 °C and stopped with equal volumes of STEB (100 mM Tris-HCl pH 8.0; 10 mM EDTA; 0.5 mg/mL Bromophenol Blue; 40% sucrose) and of chloroform:isoamyl alcohol (24:1). After vortexing and centrifuging at maximum speed for 2 min, 22 µL of the upper aqueous phase were loaded on a 1% agarose gel in 1x TAE. Samples were allowed to settle for 20 min before starting migration at 50 V for about 3 h. The gel was stained for 30 min with 1 mg/mL EtBr in 1x TAE and destained for 15 min before UV image capture via ChemiDoc XRS Quantity One Analysis Software. To determine the stabilization of the gyrase-DNA cleavage complex by MOX and M06, we used the same reaction buffer, but the reaction was incubated for 1 h at 25 °C. To denature DNA gyrase, remove DNA-bound enzyme, and reveal latent DNA breaks, 0.2% SDS and 0.1 mg/mL of proteinase K were added to the reaction, and incubated for 30 min at 37 °C. Reactions were stopped and analyzed as indicated above.

### Liquid chromatography and mass spectrometry (LC-MS) analysis of M06 metabolites

Secondary cultures of *M. tuberculosis* H37Rv were grown up to OD_600nm_ 0.8 in duplicate and treated either with 18.75 µg/mL of M06 (15-fold MIC) or with a corresponding volume of DMSO as negative control. At each time point (0, 24, and 48 h), 20 mL of culture were collected at 4200 *g* for 10 min at 4 °C. Cell pellets were resuspended in 1 mL of cold extraction solvent (40% acetonitrile; 40% methanol in ultrapure ddH_2_O) and frozen at –80 °C. Bacteria were lysed by bead beating 6 times for 45 s at 6000 RPM using a Precellys system, and incubating on ice between each cycle. Samples were centrifuged at 16600 x *g* for 10 min at 4 °C, and the resulting supernatants were decontaminated by filtration using 0.22-µm tube filters (PALL Life Sciences) and stored at –80 °C until LC-MS analysis. Cell supernatants were directly filtered through 0.22-µm filtration units and stored at 4 °C until LC-MS analysis. M06 and its metabolites were extracted from 20 mL of culture supernatant three times, by adding the same volume of water and the same volume of ethyl acetate, which was then evaporated under vacuum and the residue was dissolved in 100 µL of acetonitrile. LC-MS analysis was performed using a Q Exactive mass spectrometer (Thermo Scientific). 24 samples were analyzed, *n* = 2 biological replicates. The instrument was equipped with an electrospray ionization source (H-ESI II Probe) coupled to an Ultimate 3000 RS HPLC, endowed with the Chromeleon Chromatography Data System software (Thermo Scientific). A Thermo Scientific Hypersil GOLD aQ chromatography column (100 mm × 2.1 mm; 1.9 µm particle size) was pre-warmed at 30 °C and injected with the sample. The flow rate was set to 0.3 mL/min and the column was run for 3 min in isocratic eluent consisting of 5% acetonitrile in water containing 0.1% formic acid; 5 to 100% from 3 to 8 min. Full MS in positive mode was used with resolution set at 70,000, max IT = 240 ms, and AGC Target = 1.10^6^.

### Thin-layer chromatography of lipids, fatty, and mycolic acids

Secondary cultures of *M. tuberculosis* H37Rv, S10 NAT-mutant, and GMT35 NAT-overexpressing strain were cultured in Middlebrook 7H9 broth supplemented with 10% albumin-dextrose-catalase and 0.05% Tween 80, shaking at 120 RPM, until OD_600_ 0.15–0.2. Cultures were left untreated or treated with M06 (MIC: 1.25 µg/mL), MOX (MIC: 50 ng/mL), or INH (MIC: 100 ng/mL), at different concentrations relative to the MIC: 1x; 5x; 10x; 25x; or 50x the MIC. At the same time, cultures were labeled with either 0.5 or 1 µCi/mL of ^14^C-acetate (ARC; specific activity 106 mCi/mmol) and incubated at 37 °C under static conditions for 24 h. Lipids and mycolic acids were also analyzed in wild-type, NAT-mutant, and NAT-overexpressing strains, metabolically labeled with 1 µCi/mL of ^14^C-acetate as above, during early (OD_600_ 0.15) and mid (OD_600_ 0.5) exponential phase.

Lipids were extracted from 100 µL culture aliquots with 1.5 mL chloroform: methanol (2: 1) at 65 °C for 3 h. After extraction, 150 µL of ddWater was added, the samples were mixed, and centrifuged at 3000 x *g* for 3 min, and the lower organic phase was removed and dried under nitrogen. Extracted lipids were washed with chloroform: methanol: ddWater (4: 2: 1), dried with nitrogen, resuspended in 50 µL chloroform: methanol (2: 1), and 5 µL were loaded on silica gel 60 F254 plates (Merck). To separate trehalose esters of mycolates (TDM; TMM) and phospholipids (PE; CL), a mixture of chloroform: methanol: ddWater (20: 4: 0.5) was used as the eluent. To separate TDM, TMM, and phosphatidylinositol mannosides (PIMs), a mixture of chloroform: methanol: NH_4_OH: ddWater (65: 25: 0.5: 4) was used as the eluent. ^14^C-labeled lipid profiles were visualized using an AmershamTM TyphoonTM Biomolecular Imager and control software.

For mycolic acids extraction, 100 µL cultures were treated with 1 mL of tetrabuthylammonium hydroxide (15%) and incubated at 100 °C overnight. Samples were methylated by adding 1.5 mL of dichloromethane, 1 mL of ddWater, and 150 µL of iodomethane, and incubated at room temperature under rotation for 4 h. Methylated samples were washed twice with ddWater, and organic phases were dried with nitrogen. Fatty and mycolic acid methyl esters (FAME and MAME) were extracted with 2 mL of diethyl ether, dried, and resuspended in 50 µL chloroform: methanol (2: 1) and 5 µL were loaded on a TLC plate, which was developed in n-hexane: ethyl acetate (95: 5; 3 runs). Unsaturated forms of MAME were analyzed on Ag-impregnated silica plates. ^14^C labeled FAME and MAME were visualized as described above. Cell envelope components were quantified from TLC autoradiographs using ImageJ^71^ and normalized to untreated samples.

### Intracellular efficacy imaging assay

Confluent RAW macrophages were diluted to a concentration of 5*10^4^ cells per mL in complete DMEM without phenol red and without antibiotics, and 70 µL were seeded into each well of an 18-well µ-Slide (ibidi), and incubated at 37 °C in humidified 5% CO_2_ atmosphere for 24 h. Macrophages were infected with our *M. tuberculosis* ribosomal reporter, also constitutively expressing a red marker (rRNA-GFP_DsRed2_cyt_; GMT17)^6^. Secondary *M. tuberculosis* cultures were grown up to OD_600_ 0.4–0.6, diluted in complete DMEM without phenol red to OD_600_ 0.0025 (MOI 1:10), and medium was replaced with the bacterial cell suspension, except for uninfected control wells. After 4 h of incubation at 37 °C in a humidified 5% CO_2_ atmosphere, infected cells were washed five times with complete DMEM without phenol red, and drugs were added as follows: INH (50 ng/mL, 2x MIC); RIF (156 ng/mL, 2x MIC); M06 (1.25 µg/mL, 1x MIC); or their combinations. DMSO was used in untreated control wells. Cells were re-incubated for 6 days and stained with DRAQ7 mortality dye (3 µM) before imaging. Six images were acquired for each field of view through a z-stack of 6 µm, starting the scan from the middle of the sample, using a 100x oil immersion objective, 1.4NA, WD 0.12 mm (Olympus). Exposure conditions on the Delta Vision microscope: bright field 10% T, 100 ms; FITC (Ex 475/28, Em 525/48) 32% T, 50 ms; TRITC (Ex 542/27, Em 597/45) 32% T, 50 ms; Cy5 (Ex 632/22, Em 679/34) 32% T, 50 ms. Image stacks were projected by maximum-intensity method in SoftWorx 7.0 Cytiva, and processed using a customized ImageJ macro, which automatically segments intracellular bacterial foci based on their red fluorescence. Derived masks were used to measure size and rRNA-GFP fluorescence normalized to size (as a proxy for metabolic activity) of intracellular bacilli. Inverted masks were used to subtract the background green fluorescence from each ROI.

### Intracellular efficacy by CFU quantification

Confluent RAW macrophages were seeded into a 48-well plate, about 5*10^4^ cells/well (400 µL), and incubated for 24 h prior to infection. Log-phase *M. tuberculosis* cultures were used for infection after diluting them in DMEM either to OD_600nm_ 0.005 (MOI 1:1) or to OD_600nm_ 0.05 (MOI 1:10). Both conditions were run in duplicate. Macrophages and bacteria were incubated for 4 h at 37 °C in a humidified 5% CO_2_ atmosphere. Infected cells were washed three times with complete DMEM, and drugs were added as follows: INH (0.2 µg/mL, 8x MIC); RIF (0.624 µg/mL, 8x MIC); M06 (1.25 µg/mL, 1x MIC); or their combinations. DMSO was used in untreated control wells. Cells were re-incubated for 7 days, and washed after 2 and 4 days of incubation, also replenishing drugs. At each time point, the medium was removed, and cells were washed once with 1x PBS. For cell lysis, 400 µL of lysis buffer (0.1% Tween 80 in ddH_2_O) were added and cells were incubated at 37 °C for 10 min. Cellular lysis was checked under an inverted microscope. Cells were scraped with a P1000 tip and pipetting 5 times before serial dilution into fresh 7H9 medium, and plating onto 7H11 plates. Plates were incubated for 6 weeks prior to CFU counting. CFU were expressed as a percentage of survival relative to time zero.

### Statistics

Unless otherwise specified, plots and statistical analyses were done with GraphPad Prism 9.5.0. Plots merge datasets deriving from at least two biologically independent replicates, and statistics were derived from *n* ≥ 3. *P-*values, sample sizes, and statistical tests are reported in each figure or figure legend.

### Reporting summary

Further information on research design is available in the Nature Portfolio Reporting Summary linked to this article.

### Supplementary information

Supplementary Information

Peer Review File

Description of Additional Supplementary Files

Supplementary Data 1

Supplementary Data 2

Supplementary Data 3

Supplementary Data 4

Supplementary Data 5

Supplementary Data 6

Supplementary Data 7

Supplementary Data 8

Supplementary Movie 1

Supplementary Movie 2

Supplementary Movie 3

Supplementary Movie 4

Reporting Summary

### Source data

Source Data

## Data Availability

We have no restrictions on data availability. Source data are provided in this paper. Time-lapse image stacks and snapshot images of mycobacterial cells alone or during infection under drug treatment generated in this study have been deposited in the DRYAD database under the unique digital object identifier [10.5061/dryad.r4xgxd2j8]. Raw data from whole transcriptome and whole-genome sequencing have been deposited in the EMBL’s European Bioinformatics Institute, under the EBI accession codes E-MTAB-12306 (RNA-seq) and E-MTAB-12307 (WGS). The processed data derived from single cells, microcolonies, CFU, MIC, TLC, LC-MS, and RNA/DNA-Seq generated in this study are provided in the Supplementary Information/Source Data file. The *M. tuberculosis* PANTHER genome information used in this study is available in the GO Ontology database under the accession code MYCTU. The structure of the arylamine N-acetyltransferase^36^ used in this study is available at the RCSB Protein Data Bank (RCSB PDB) under the accession code 4BGF. The structure of the DNA gyrase from *M. tuberculosis*^30^ used in this study is available at the RCSB PDB under the accession code 5BS8. The interaction network of *M. tuberculosis* NAT used in this study is available at the STRING Core Data Resource [https://string-db.org/cgi/network?taskId=bvahymk8E36h&sessionId=bAEKEPVHSkc2]. Source data are provided in this paper.

## Code availability

Detailed functions and scripts used for the analysis of the PTC screening were packaged and are available at Gitlab [https://gitlab.pasteur.fr/svolant/ptc-screening/]. The custom Omnipose model and Python notebook used for single-cell snapshot analysis are available at Gitlab [https://gitlab.pasteur.fr/iah-public/automated_segmentation_mycobacterium_tuberculosis_snapshots]. The ImageJ macro (Macro_Analysis_THP1_MTUB.ijm) generated in this study to analyze infected macrophages is available at DRYAD.
