# Supplementary material for: Dynamic microfluidic single-cell screening identifies pheno-tuning compounds to potentiate tuberculosis therapy
Source: Nat Commun. 2024 May 16;15:4175. doi: 10.1038/s41467-024-48269-2 (PMC11099131; doi:10.1038/s41467-024-48269-2)
Supplement: Supplementary file 1 — Supplementary Information [file 41467_2024_48269_MOESM1_ESM.pdf]

Supplementary Information

Supplementary Figures

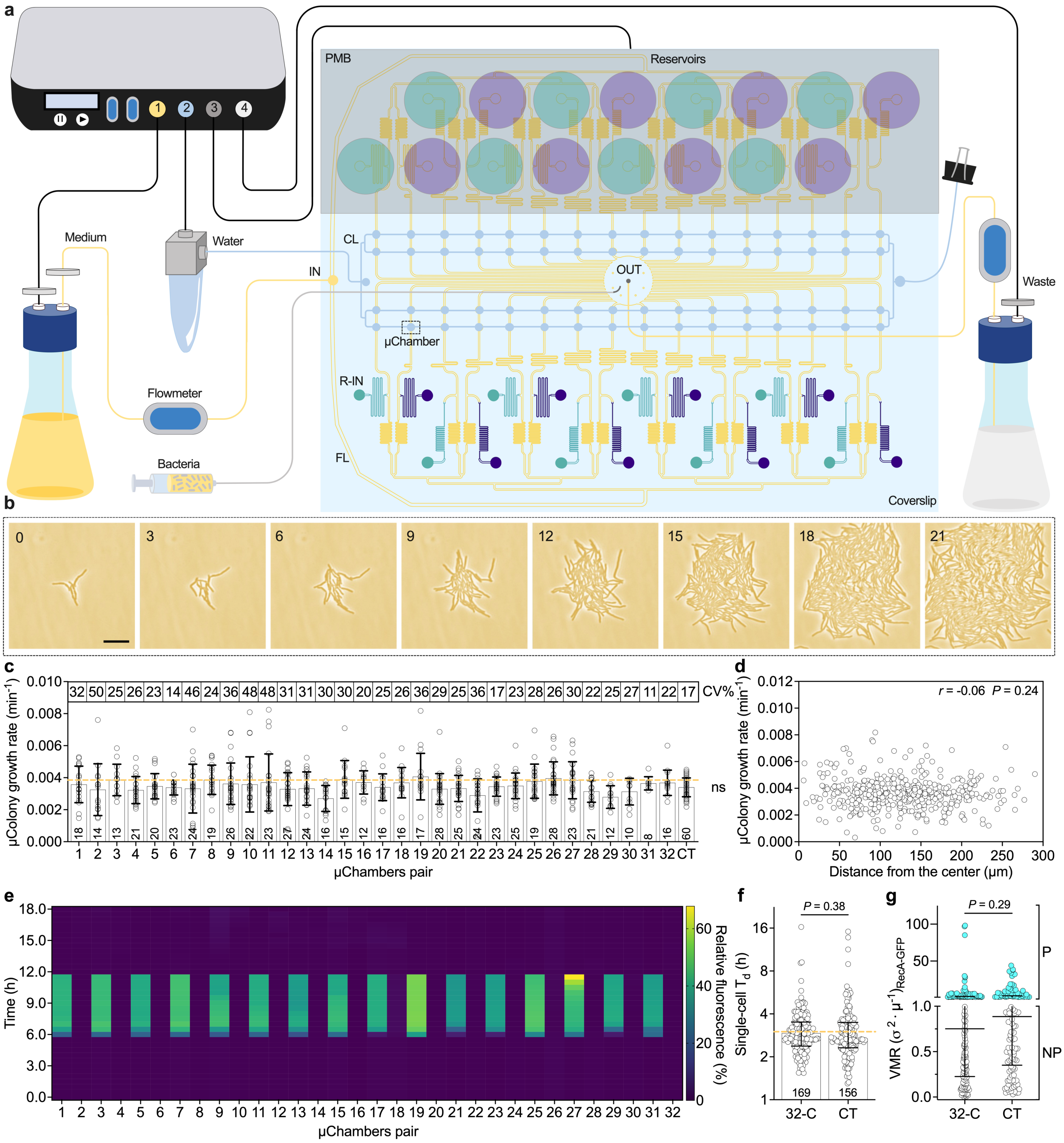

Supplementary Fig. 1. Operation and characterization of the 32-condition platform. a Schematic of the 32-condition microfluidic device connected to a 4-channel software-driven flow controller. The first channel actuates the injection of medium into the flow layer (FL, yellow) by the inlet port (IN). The microfluidic network branches to 32 pairs of circular microchambers (1-mm diameter), which share the same outlet port (OUT). The OUT is connected to a waste receptacle, controlled by the fourth channel. To prevent contaminations, 0.2-µm filters (gray cylinders) are placed at the inlet and outlet ports of the medium bottle and at the waste outlet port. The OUT is also used to load the bacterial suspension. The flow of medium is regulated upstream and downstream of the device by means of two software-driven flowmeters. The FL is bound to the underlying coverslip, enabling inverted microscopy. The second channel actuates water into the control layer (CL, light

blue), which is closed at one end with a binder clip. The third channel actuates the injection of different solutions from the reservoirs (larger green and purple circles) of a perforated metal block (PMB, gray rectangle). The PMB is superimposed on the reservoir inlets (R-IN, smaller green and purple circles). **b** Representative phase-contrast image series of *M. smegmatis* growing inside one microchamber of the device (dashed square). Images are pseudocolored yellow. Cells were imaged at 20-min intervals and numbers represent hours. Scale bar = 5 µm. **c** Microcolony growth rates of *M. smegmatis* growing inside the different microchambers (X-axis), compared to our control (CT) device[1]. Total number of microcolonies indicated in the bars, from at least n = 2 biologically independent experiments. Error bars represent mean ± SD. Numbers in the insets indicate the percent coefficient of variation per microchamber. No significant difference (ns) between group means by two-way ANOVA followed by Tukey's multiple comparisons test, $F(32,596) = 1.879$, P = 0.0027. Dashed line indicates the average growth-rate of *M. smegmatis* in flask. **d** Two-tailed Spearman correlation between grow rate and position of microcolonies within the microchamber, n = 454 pairs from at least n = 2 biologically independent replicates. **e** Quantification of fluorescence in the channel connecting each pair of microchambers during perfusion of non-fluorescent medium alone for 18 hours, and injection of a 100-µM concentrated FITC solution from alternate reservoirs for 6 hours. Intensity values are normalized to the fluorescence of the stock FITC-solution (100 µM) perfused inside the entire microfluidic network for 6 hours and are expressed as a percentage, n = 2 independent replicates. These results indicate that a given stock solution stored in the reservoir is diluted on average by 2.4±0.4 fold as it passes through the microfluidic tree until it reaches the microchambers. **f**,**g** Single-cell doubling time (T_d, **f**) and variance to the mean ratio (VMR, **g**) of RecA-GFP fluorescence over the lifetime of single *M. smegmatis* cells growing inside the 32-condition platform (32-C) compared to our control (CT) hexa device[1]. Total number of cells indicated in the bars, from at least n = 2 biologically independent experiments. Error bars represent median with interquartile range. Significance by non-parametric Kolmogorov–Smirnov test, 95% confidence level. Dashed line indicates the average bulk T_d of *M. smegmatis* in flask (**f**). Under-dispersed VMR (≤ 1) corresponds to RecA-GFP non-pulsing cells (NP, white circles), and dispersed VMR (> 1) to pulsing cells (P, turquoise circles) (**g**). Source data are provided as a Source Data file.

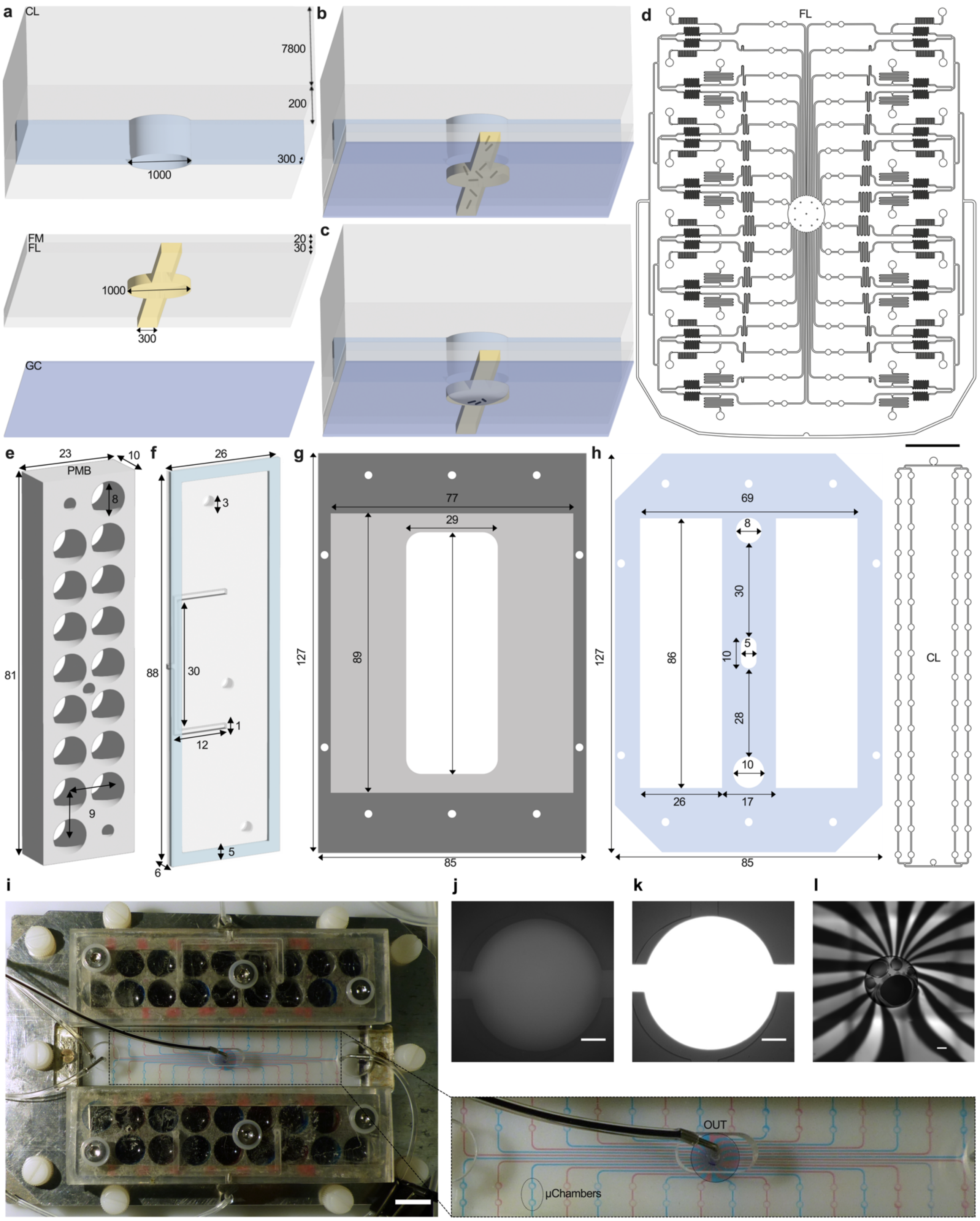

Supplementary Fig. 2. Design and structural components of the 32-condition microfluidic platform. a−c Not-to-scale axonometric projections of the components of the microchamber (viewed from the bottom). From top to bottom (**a**): control layer filled with water (CL, light blue); flexible membrane (FM); flow layer filled with growth medium (FL, yellow); glass coverslip (GC). Numbers represent dimensions in μm. Microchamber assembled layers, with bacteria (black rods) perfused in the FL, without (**b**), or with (**c**) pressure applied from the CL. Bacteria are trapped between FM and GC as the pressure increases in the CL. **d**, Mask design of the FL and CL (Supplementary Data 1). Scale bar = 10 mm. **e**,**f** Axonometric projections of the perforated metal block (PMB) forming 32 reservoirs (**e**) and of its sealing lid, used for air injection (**f**). Numbers represent dimensions in mm.

g,**h** Schematics of the metal (**g**) and acrylic (**h**) holders used to mount the 32-condition device on the stage of an inverted wide-field microscope. The maximum area corresponds to a standard multi-well plate. Numbers indicate dimensions in mm. **i**, Picture of the 32-condition platform assembly, upon alternate injection of red and blue dyes from separate reservoirs. Scale bar = 10 mm. A magnification of the core of the platform is shown (dashed rectangle), where the common outlet port (OUT, dotted circle) and a pair of microchambers (dotted oval) are also indicated. **j**,**k**,**l** Representative fluorescence pictures of microchambers perfused either with non-fluorescent medium (**j**) or with a FITC solution (**k**), and of the outlet port of the device (circle) receiving non-fluorescent and fluorescent medium from the outlet channels (alternate dim and bright segments) of each pair of microchambers, showing proper separation of the conditions (**l**). Scale bars = 200 μm.

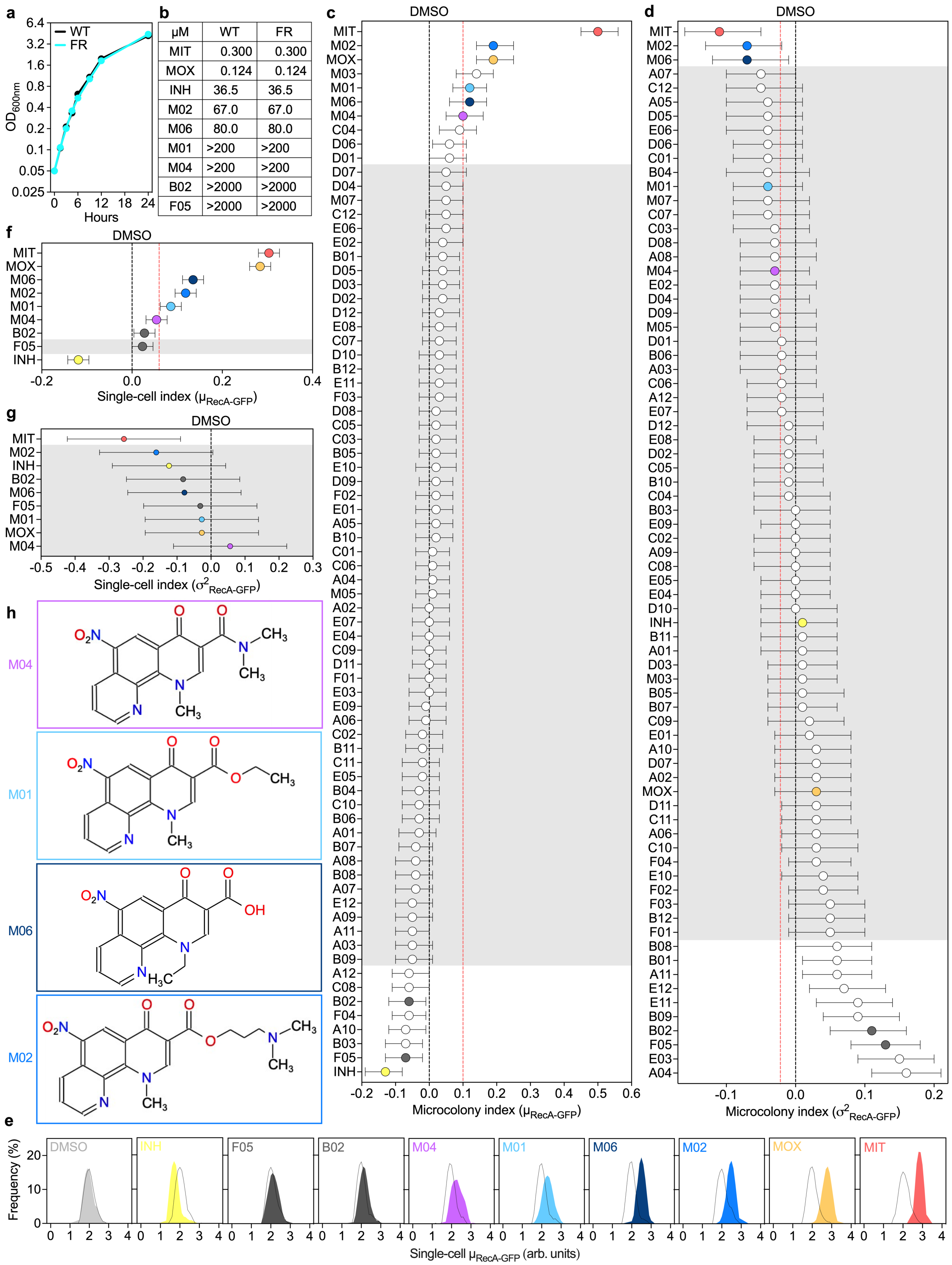

μM	WT	FR
MIT	0.300	0.300
MOX	0.124	0.124
INH	36.5	36.5
M02	67.0	67.0
M06	80.0	80.0
M01	>200	>200
M04	>200	>200
B02	>2000	>2000
F05	>2000	>2000

Supplementary Fig. 3. Effect-size estimates of the impact of PTC on *M. smegmatis* RecA-GFP. **a** Bulk growth kinetics of wild type (WT) *M. smegmatis* and dual-fluorescent reporter (FR) strain. Mean values are shown, n = 2 biologically independent experiments. **b** MIC of mitomycin C (MIT), moxifloxacin (MOX), isoniazid (INH) and six PTC against *M. smegmatis* WT and FR strains. Mean values, n = 2. **c**,**d**,**f**,**g** Comparison of the size of PTC effects on (**c**,**f**) average ($\mu_{RecA\text{-}GFP}$) and (**d**,**g**) variance ($\sigma^2_{RecA\text{-}GFP}$) of fluorescence levels as recorded in microcolonies (**c**,**d**) and in single-cell (**f**,**g**)

setups. Effect sizes reflect difference between DUR versus PRE stages. Black dashed lines represent negative control DMSO. Red dashed lines represent 20% of the effect of MIT (red circle). Error bars represent mean ± 95% confidence interval. For each stage, at least n = 6 time points were measured for each microcolony. Each molecule was replicated up to 8 times for each experiment, and 15 experiments were performed. White and gray areas represent significant and non-significant indices, respectively, which were estimated by mixed-effects models. **e** Distribution of log_{10} single-cell $\mu_{RecA\text{-}GFP}$ at the end of PRE (white) and DUR (colored) stages, at least n = 90 up to 349 single cells per condition, from at least two biologically independent experiments. **h** Chemical structure of PTC hits (Supplementary Data 2). Source data are provided (Supplementary Data 3).

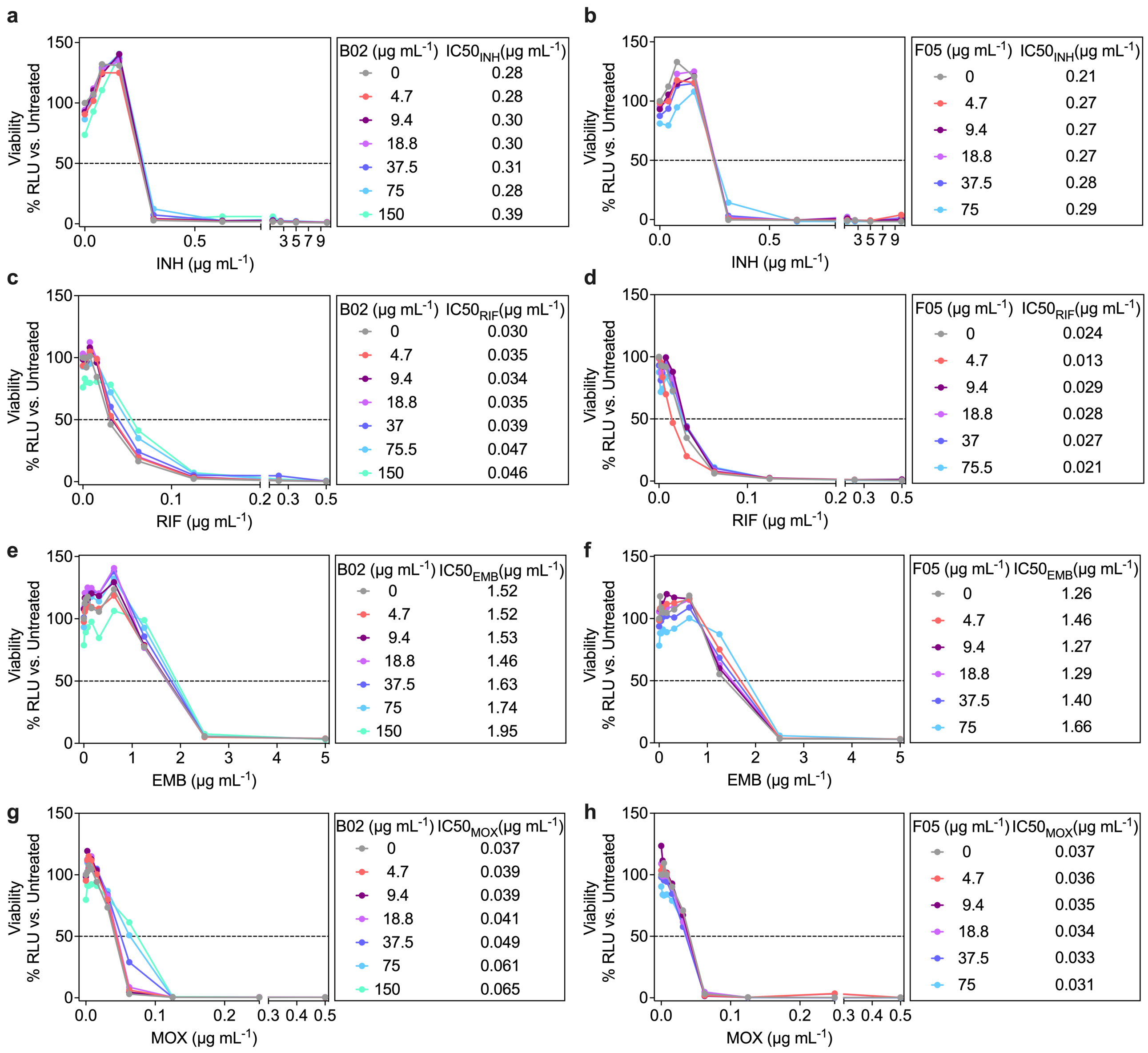

Supplementary Fig. 4. In-vitro activity of anti-tubercular drugs in combination with PTC B02 and F05. a−h Dose-response assays of INH (**a**,**b**), RIF (**c**,**d**), EMB (**e**,**f**), and MOX (**g**,**h**) in combination with different subinhibitory concentrations of B02 (**a**,**c**,**e**,**g**) and F05 (**b**,**d**,**f**,**h**). Symbols represent mean, *n* = 3 (**a**,**c**,**e**,**g**) and *n* = 2 (**b**,**d**,**f**,**h**) biologically independent experiments. *M. tuberculosis* viability was estimated from ATP production after one week of drug exposure compared to untreated cells. Half maximal inhibitory concentrations (IC50) are shown for all drugs at each PTC concentration. Source data are provided as a Source Data file.

a

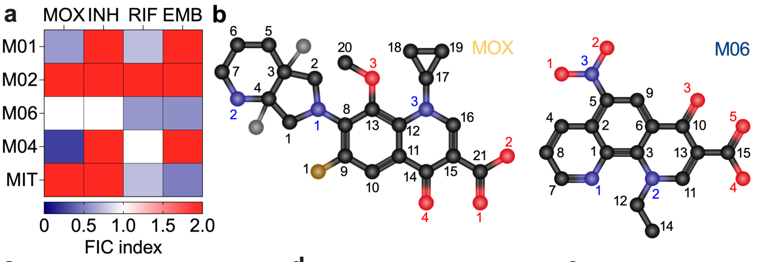

c

d

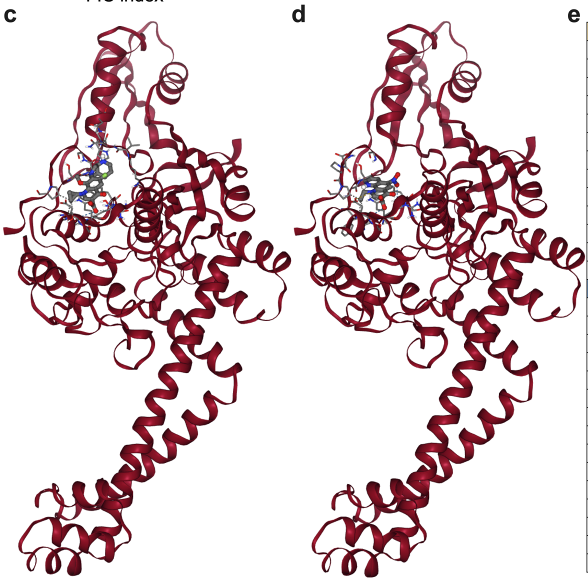

e

MOX affinity -7.1 kcal mol^{-1}	
Hydrophobic contact	
Atom	Amino acid
C19	D94
C19	V97
C19	P124
Hydrogen bond	
Atom	Amino acid
O2	D94
N2	P119
Weak hydrogen bond	
Atom	Amino acid
O2	S95
O2	R98
M06 affinity -6.6 kcal mol^{-1}	
Hydrophobic contact	
Atom	Amino acid
C14	D94
C14	V97
C14	P124
Hydrogen bond	
Atom	Amino acid
O4	D94
O2	S104
O2	W103
O2	S104
Weak hydrogen bond	
Atom	Amino acid
C8	S118
O3	R98

Supplementary Fig. 5. In-vitro and in-silico analyses of PTC activity against *M. tuberculosis*. **a** Heatmap displaying mean fractional inhibitory concentration (FIC) indices calculated for combinations of PTC and MIT with anti-tubercular drugs: indifference (FIC > 1); additivity (0.5 < FIC ≤ 1), synergy (FIC ≤ 0.5). Experiments were repeated at least twice independently. **b** CPK-colored chemical structures of MOX and M06. Molecular topology is also indicated. **c−e** In silico docking poses of MOX (**c**) and M06 (**d**) in *M. tuberculosis* DNA gyrase (PDB 5BS8)[2], and table of predicted molecular interactions (**e**).

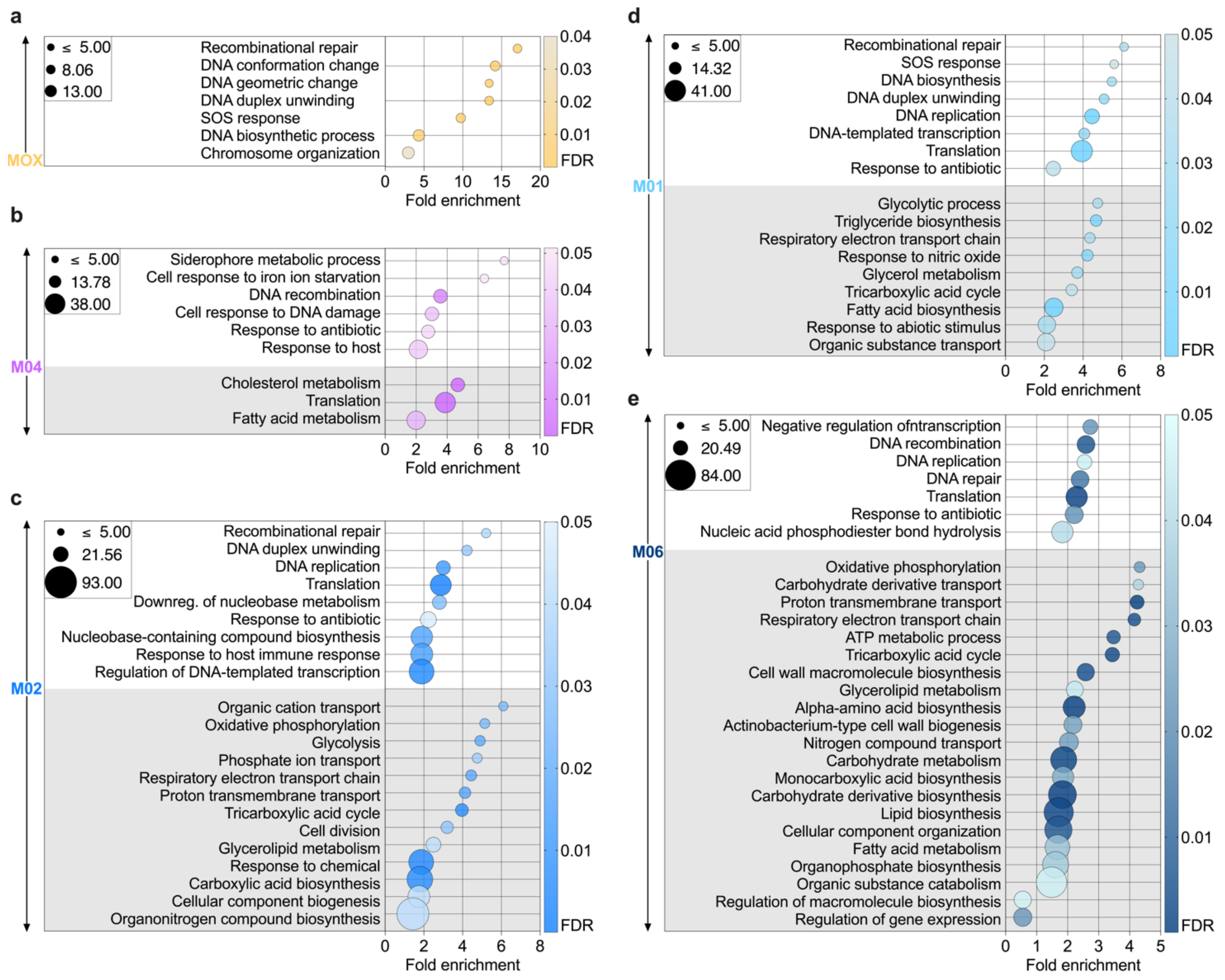

Supplementary Fig. 6. GO enrichment analysis of DEGs in *M. tuberculosis* treated with PTC hits and MOX. a−e Complete GO biological processes were identified by overrepresentation test of significantly up-regulated genes (white shading) and down-regulated genes (gray shading), upon treatment with MOX (**a**); M04 (**b**); M02 (**c**); M01 (**d**); and M06 (**e**), against all *M. tuberculosis* genes in PANTHER database[3,4]. Raw *P*-values were determined by Fisher's exact test, and FDR < 0.05 was calculated by Benjamini-Hochberg method. Bubble plots reports the most specific functional categories sorted by fold enrichment. The entire hierarchies of significantly enriched functional categories for up- and down-regulated genes are available (Supplementary Data 5).

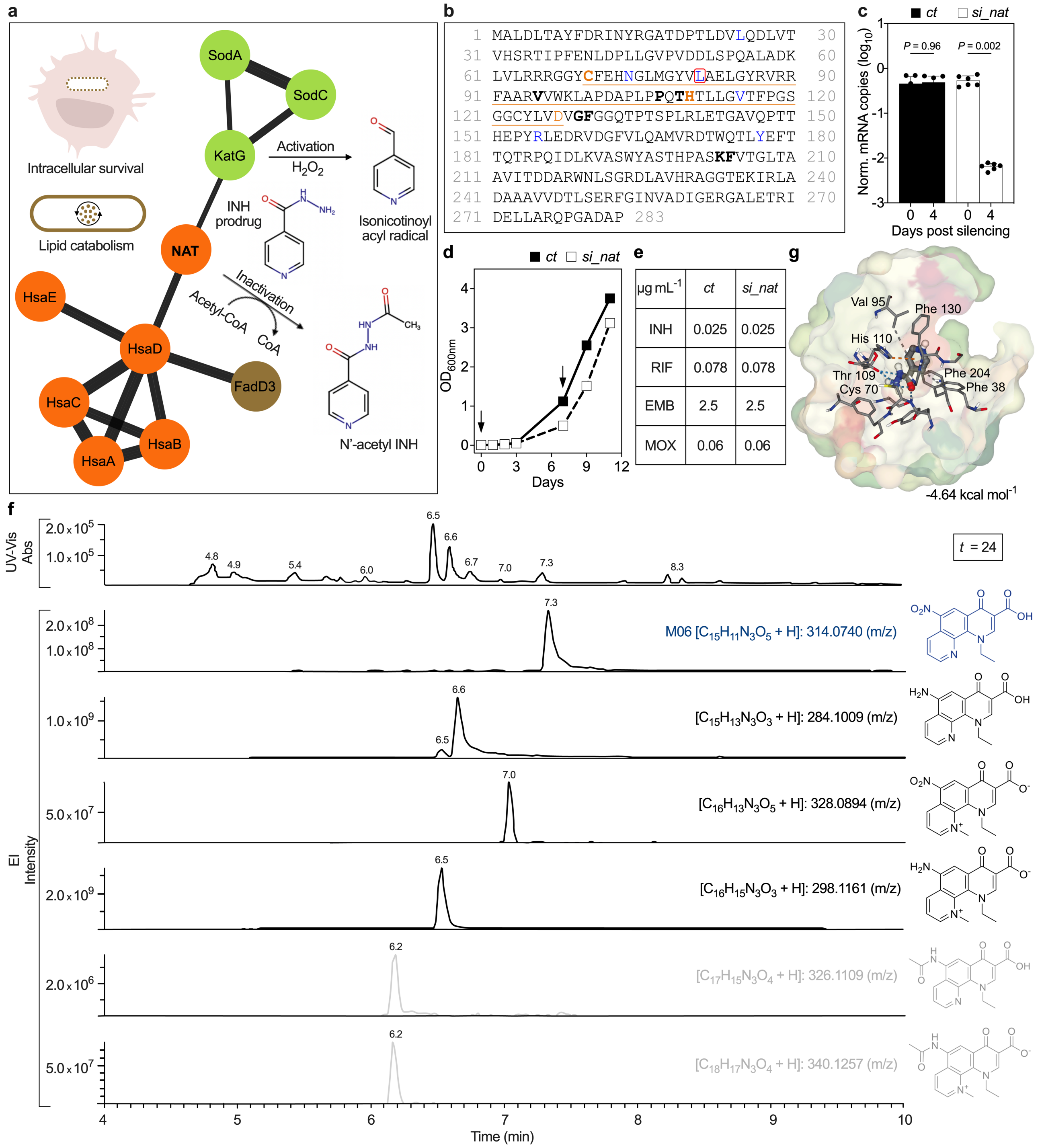

Supplementary Fig. 7. Role of NAT in mycobacteria. a Highest confidence interaction network of *M. tuberculosis* NAT, with no more than 5 interactors in the first shell and maximum seven interactors in the second shell, modified from STRING (https://string-db.org/)[5]. The thickness of the connections is associated with the likelihood of interaction. Activation and inactivation of INH prodrug by KatG and NAT, respectively, are sketched on the right of the network and other roles of NAT are sketched on the left. **b** *M. tuberculosis* NAT protein sequence[6], with numbered amino acids. The active site is underlined, with residues belonging to the catalytic triad colored orange, and residues implicated in protein structure stability colored blue. Leucine 81, which is deleted in PTC-resistant mutants, is circled in red. Residues that potentially interact with M06 are bolded. **c** qRT-PCR analysis of *M. tuberculosis nat* before and after induction of a dCas9 silencing system together with a scramble sgRNA (*ct*) or with an anti-*nat* sgRNA (*si_nat*). Transcripts are normalized to total RNA and *sigA* mRNA copies. Error bars represent mean ± SD, n = 3 biologically independent experiments. Significance by two-way ANOVA followed by Šidák correction for multiple comparisons, 95% confidence interval, $F(1,10) = 12.34$, $P = 0.0056$. **d** Growth kinetics of *M. tuberculosis si_nat* and *ct*

strains. Arrows indicate the addition of ATC to induce silencing. Symbols represent mean, *n* = 2 biologically independent experiments. **e** MIC of different anti-tubercular drugs against *M. tuberculosis si_nat* and *ct* strains, *n* = 2 independent experiments. **f** Representative UV spectrum at 270 nm and EI chromatograms of *M. tuberculosis* cell extract upon 24-hour treatment with M06 (15-fold MIC). The experiment was repeated twice independently with similar results. X-axis indicates time after injection (min). Y-axis indicates intensities (arb. units). Chemical structures, formulas, and mass-to-charge ratio of M06 and its metabolites are shown next to the relevant peaks. **g** In-silico docking pose of CPK-colored INH in the catalytic pocket of NAT[7]. Binding affinity is indicated (kcal/mol). Predicted amino acid residues in hydrophobic contact (dark gray); cation-pi interaction (orange); and linked by either weak (light gray) or strong (blue) hydrogen bonds with INH atoms are shown. Source data are provided as a Source Data file.

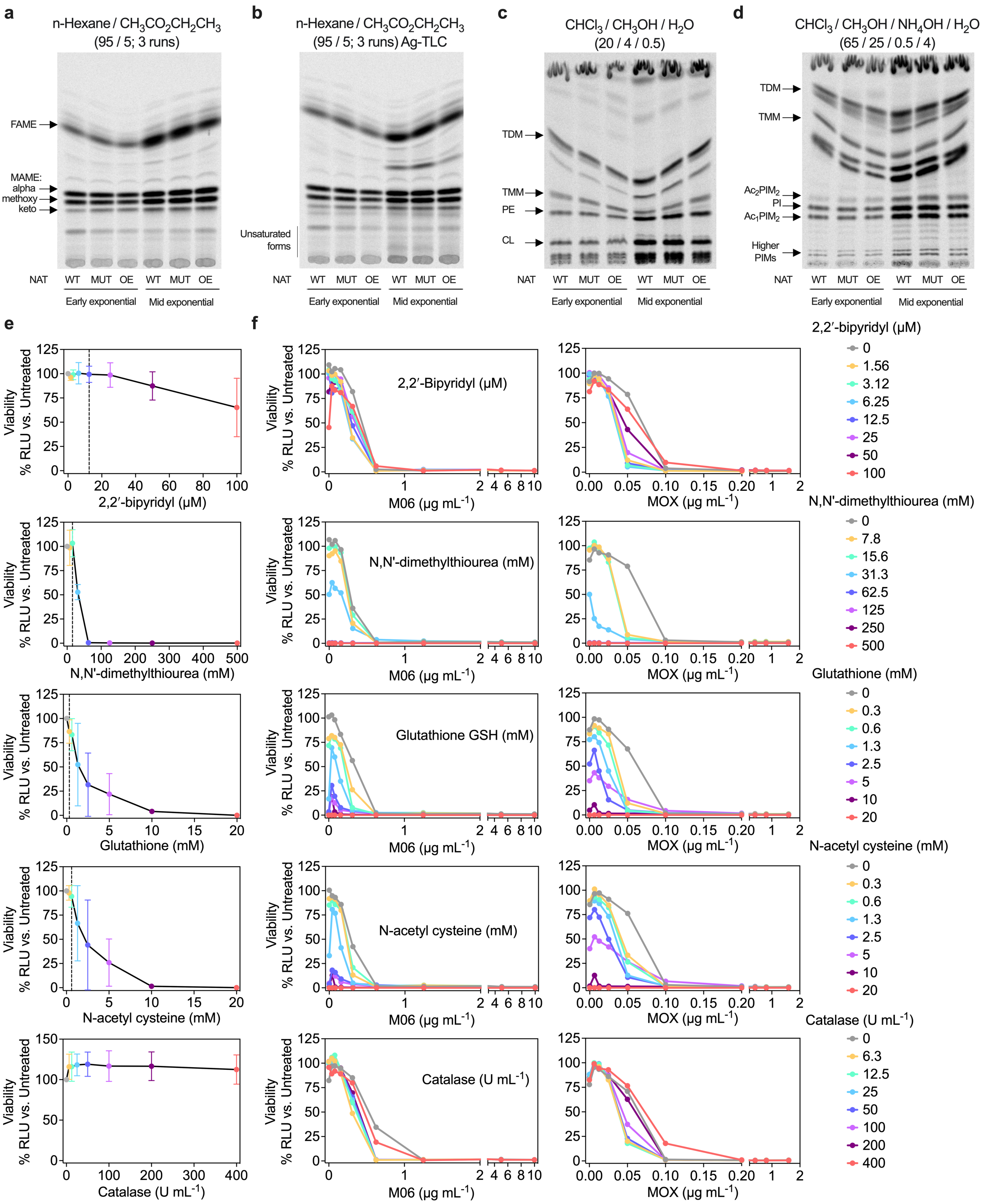

Supplementary Fig. 8. Analysis of lipids with different NAT variants and effect of ROS scavengers. a−d Representative TLC of fatty and mycolic acids and lipids extracted from *M. tuberculosis* WT; S10 mutant (MUT); and NAT overexpressing strain (OE) in early and mid-exponential phase, after 24-hour incorporation of ^{14}C-acetate (1 μCi mL^{-1}). Conditions for separation of different lipid populations are shown above the TLC autoradiographs. Fatty acid methyl esters (FAME) and mycolic acid methyl esters (MAME) (**a**). Unsaturated forms of MAME (**b**). Trehalose dimycolate (TDM); trehalose monomycolate (TMM); phosphatidylethanolamine (PE); cardiolipin (CL) (**c**). TDM; TMM; phosphatidylinositol (PI); phosphatidylinositol mannosides (PIM); acylated forms of PIM ($Ac_{2/1}PIM_2$) (**d**). Experiments were repeated twice independently with similar results. **e,f** ATP production in *M. tuberculosis* exposed to increasing concentrations of different ROS scavengers (**e**),

and to combinations of ROS scavengers with either M06 or MOX (**f**) compared with untreated cells. Error bars represent mean ± SD, *n* = 4 independent experiments, dashed lines separate subinhibitory from inhibitory concentrations of ROS scavengers (**e**). Symbols represent mean, *n* = 2 independent experiments (**f**). None of the ROS scavengers tested increases the IC50 of M06. Source data are provided as a Source Data file.

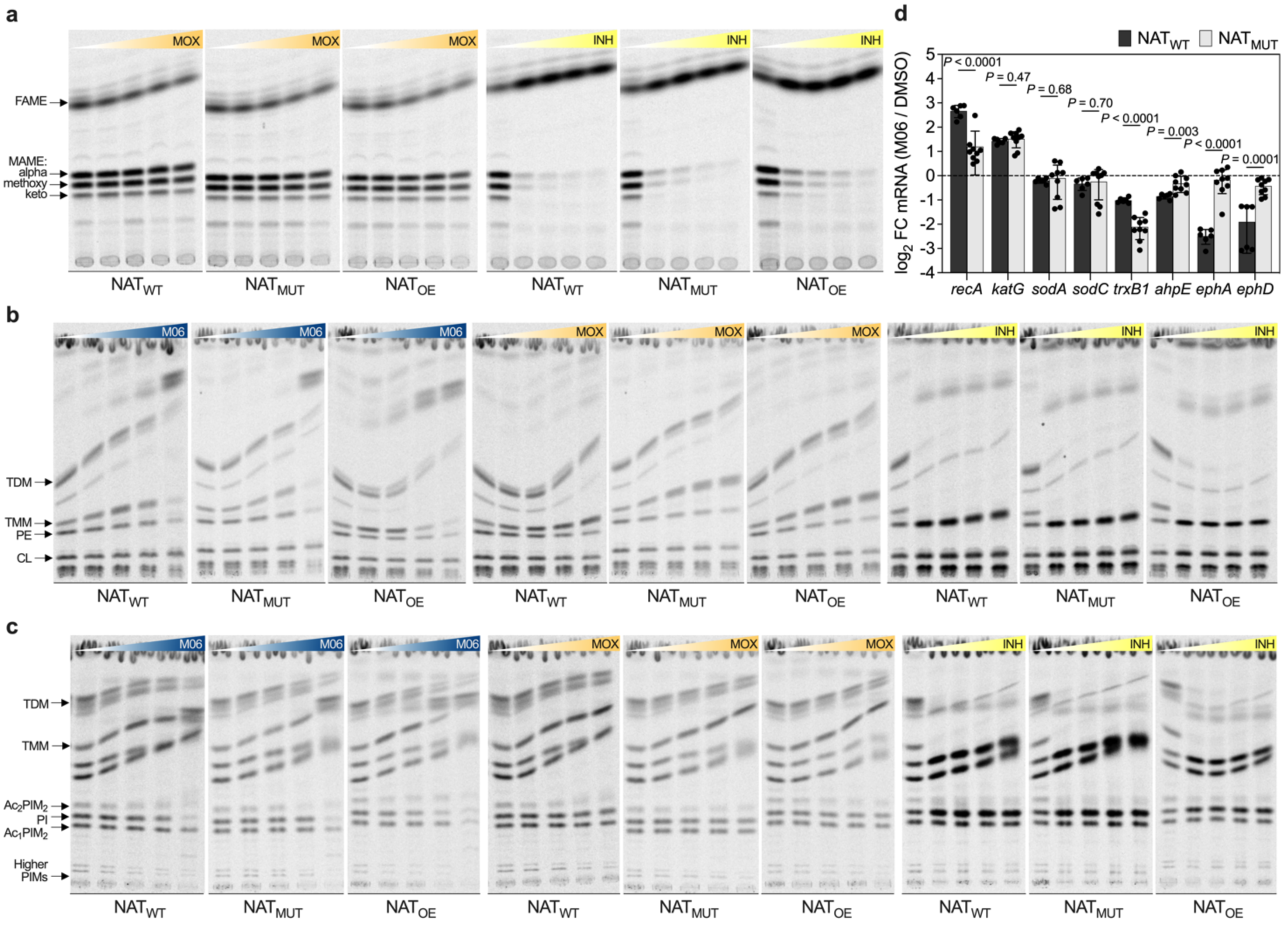

Supplementary Fig. 9. Lipid and transcriptional changes in drug-stressed *M. tuberculosis*. a−c Representative TLC of fatty/mycolic acids methyl esters (FAME/MAME) and lipids from exponentially growing *M. tuberculosis* WT (NAT_{WT}); S10 mutant (NAT_{MUT}); and NAT overexpressing strain (NAT_{OE}), developed in *n*-hexane:ethyl acetate (95:5, v/v, 3 runs). Experiments were repeated twice independently with similar results. Color gradients indicates from left to right untreated bacilli (white) or treatment with increasing concentrations of M06 (blue, MIC: 1.25 µg/mL), moxifloxacin (orange, MIC: 50 ng/mL) and isoniazid (yellow, MIC: 100 ng/mL) equal to 5X, 10X, 25X and 50X the MIC. FAME and different types of MAME (**a**). Trehalose dimycolate (TDM); trehalose monomycolate (TMM); phosphatidylethanolamine (PE); cardiolipin (CL), developed in chloroform:methanol:water (20:4:0.5) (**b**). TDM; TMM; phosphatidylinositol (PI); phosphatidylinositol mannosides (PIM); acylated forms of PIM ($Ac_{2/1}PIM_2$), developed in chloroform:methanol:NH_4OH:water (65:25:0.5:4) (**c**). **d** qRT-PCR analysis of *M. tuberculosis* WT (NAT_{WT}) and averaged S9, S10 and S11 NAT mutants (NAT_{MUT}). Transcripts are normalized to total RNA and *sigA* copies and are expressed as $\log_2$ FC between 24-hour treatment with M06 versus DMSO. Error bars represent mean ± SD, $n = 3$ independent experiments. Significance between NAT_{WT} and NAT_{MUT} strains is assessed by multiple unpaired Welch *t*-tests, corrected by Holm-Šidák method. Source data are provided as a Source Data file.

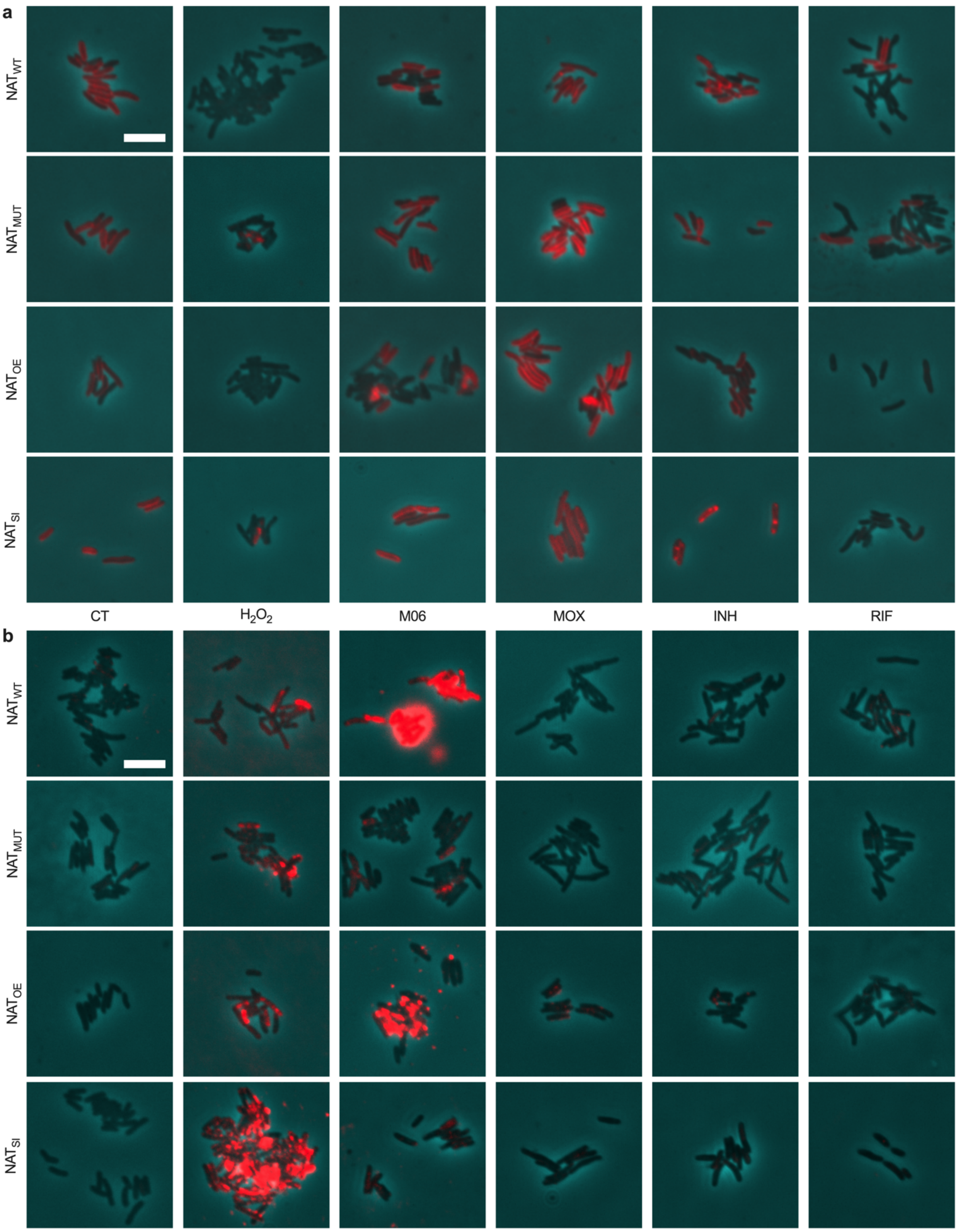

Supplementary Fig. 10. Single-cell imaging of cell envelope and oxidative stress. a,b Representative snapshot images of *M. tuberculosis* expressing different variants (NAT_{WT} and NAT_{MUT}) or different levels of *nat* (NAT_{OE} and NAT_{SI}) after 24-h exposure to DMSO (CT), H_2O_2 (30 mM) or to different drugs (10-fold MIC), *n* = 2 biologically independent experiments. Bacteria were stained with either FM464 for cell-envelope lipids (**a**) or with CellROX for oxidative stress (**b**) and imaged by phase contrast (cyan) and fluorescence (red). Fluorescence images are scaled to the brightest frame (**a**), or to a low-fluorescence image (**b**). Scale bars = 5 µm.

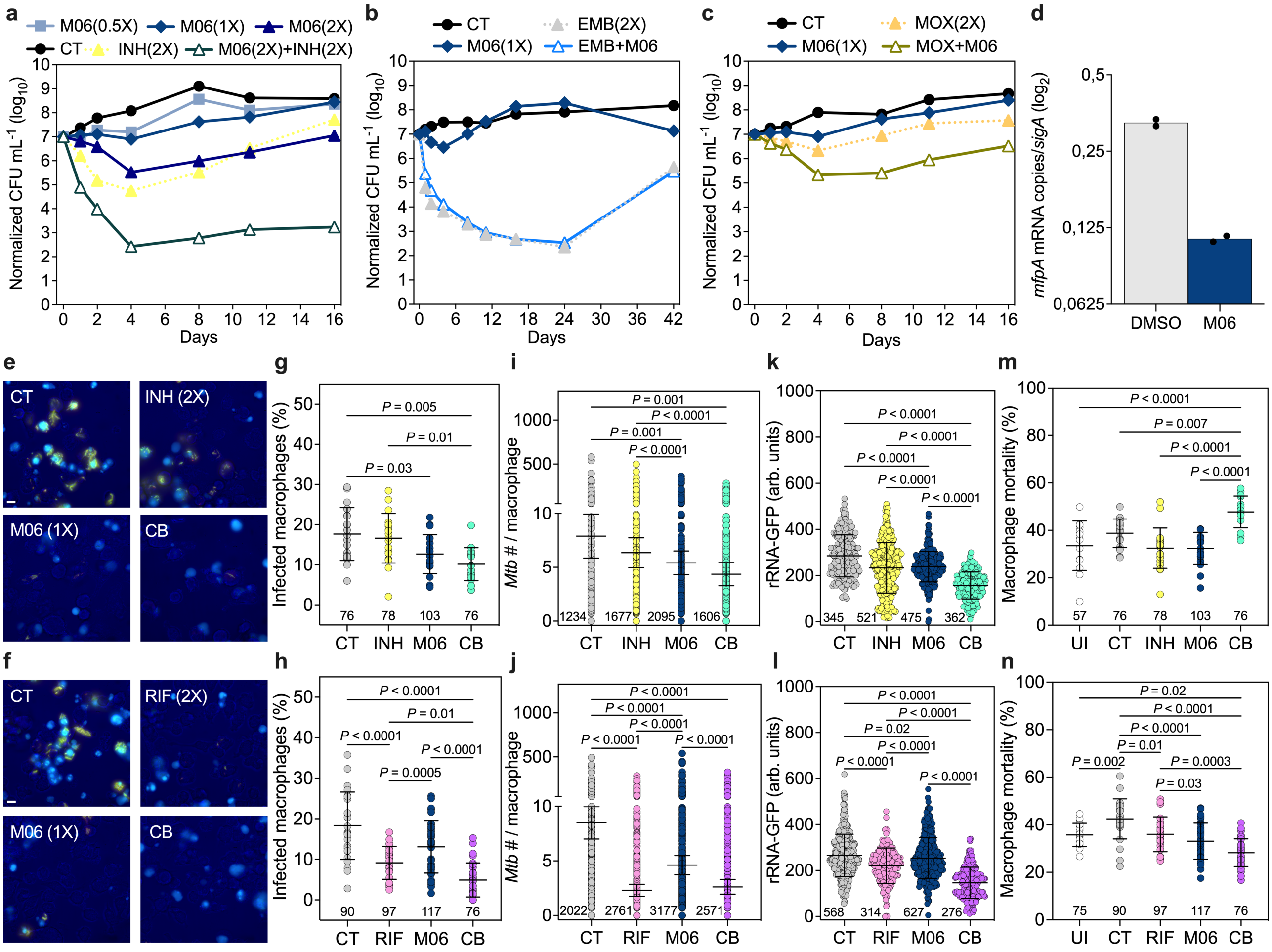

Supplementary Fig. 11. Analysis of M06 activity in combination. a−c *M. tuberculosis* growth (CT, circles), and efficacy of M06 alone: 0.5-fold MIC (squares), 1-fold MIC (diamonds), 2-fold MIC (triangles), and of INH (**a**), EMB (**b**), and MOX (**c**) alone at 2-fold MIC (triangles and dotted lines) or in combination with M06 (open triangles and solid lines). CFU are normalized to 10^7 at time zero. Symbols represent mean of at least n = 2 independent experiments. **d** qRT-PCR analysis of *M. tuberculosis rv3361c* (*mfpA*) after DMSO or M06 treatment (10-fold MIC) for 24 hours. Transcripts are normalized to total RNA and *sigA* copies and are expressed as $\log_2$ fold change before and after treatment. Bars represent mean, n = 2 independent experiments. **e**,**f** Representative snapshot images of RAW 264.7 macrophages at 6 days post infection with rRNA-GFP_DsRed2$_{cyt}$ reporter (n = 6). DMSO as a control (CT) and treatment with either INH (**e**) or RIF (**f**) alone or in combination (CB) with M06. Concentration relative to the MIC is indicated in brackets. Bright field (blue), rRNA-GFP (green), DsRed2 (red), and DRAQ7 (cyan) fluorescence are merged. Scale bars = 10 µm. **g**,**h**,**m**,**n** Fraction of infected (**g**,**h**) and dead macrophages (**m**,**n**) at 6 days post infection treated with single or combined drugs as in **e** and **f**. Mortality for uninfected (UI) cells is also reported. Total number of fields of view is shown at the bottom of the graphs from n = 6 biologically independent experiments. Error bars represent mean ± SD. Significance by two-way ANOVA followed by Tukey's multiple-comparison test, 95% confidence interval: $F(2.836,208.9)=6.304$, P = 0.0005 (**g**); $F(2.656,224.9)=27.55$, $P < 0.0001$ (**h**); $F(4,367)=11.44$, $P < 0.0001$ (**m**); $F(3.385,276.7)=15.83$, $P < 0.0001$ (**n**). **i**,**j** Number of intracellular bacilli at 6 days post infection in the conditions indicated in **e** and **f**. The number of bacilli was estimated from the area of the clump divided by the average size of a single bacillus. Total number of macrophages is shown at the bottom of the graphs from n = 6 biologically independent experiments. Error bars represent mean ± 95% confidence interval. Significance by Kruskal-Wallis' test followed by Dunn's multiple-comparison test. **k**,**l** rRNA-GFP fluorescence of intracellular bacilli at 6 days post infection in the conditions indicated in **e** and **f**. Total number of intracellular foci is shown at the bottom of the graphs from n = 6 biologically independent experiments. Error bars represent mean ± SD. Significance by two-way ANOVA followed by Tukey's multiple comparisons test, 95% confidence interval: $F(3,1332)=107.8$, $P < 0.0001$ (**k**); $F(3,1374)=102.8$, $P < 0.0001$ (**l**). Source data are provided as a Source Data file.

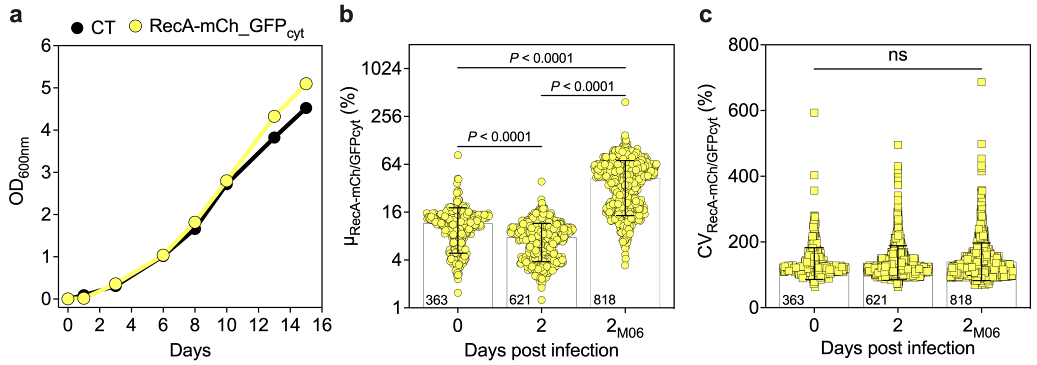

Supplementary Fig. 12. Characterization of RecA-mCherry_GFP$_{cyt}$ *M. tuberculosis* reporter. a Bulk growth kinetics of control (CT) *M. tuberculosis* and dual-fluorescent reporter strain (RecA-mCherry_GFP$_{cyt}$). Symbols represent mean, *n* = 2 biologically independent experiments. **b**,**c** RecA-mCherry fluorescence (**b**) and coefficient of variation of fluorescence (**c**) normalized to constitutive GFP$_{cyt}$ control of *M. tuberculosis* inside THP-1 macrophages in the absence or presence of M06 (1-fold MIC). Total number of intracellular foci is indicated in the bars, from *n* = 4 biologically independent experiments. Error bars represent mean ± SD. Significance by one-way ANOVA followed by Kruskal-Wallis' test, 0.05 alpha-threshold: ns = not significant. Source data are provided as a Source Data file.

Supplementary Table 1. Strains and plasmids used in this study.

REAGENT	SOURCE	IDENTIFIER
**Bacterial strains and Cell lines**		
Escherichia coli TOP10	ThermoFisher	#C404010
Mycobacterium smegmatis mc^2155	Lab collection	ATCC 700084
Mycobacterium tuberculosis Erdman	Lab collection	ATCC 35801
Mycobacterium tuberculosis H37Rv	Lab collection	ATCC 27294
ATCC 700084 transcriptional reporter of *recA* (*MSMEG_2723*), with destabilized GFP under the control of native *recA* promoter	Lab collection[1]	GMS2
GMS2 constitutively expressing wild type mCherry	This paper	GMS2_pGM218
ATCC 35801 transcriptional reporter of *rrs*, with destabilized GFP under the control of native rRNA promoter, also constitutively expressing DsRed2	Lab collection[8]	GMT17
ATCC 27294/pGM321 constitutively expressing *nat* (*rv3566c*)	This paper	GMT35
ATCC 27294/pGM315; *nat* (*rv3566c*) CRISPRi/dCas9	This paper	GMT36
ATCC 35801/pGM322 translational reporter of *recA* (RecA-mCherry) and constitutively expressing cytosolic *gfp* (GFP_{cyt})	This paper	GMT37
ATCC 27294 carrying gyrase point mutation (A90V)	CIP111339	PM_1
ATCC 27294 carrying gyrase point mutation (S91P)	CIP111343	PM_2
ATCC 27294 carrying gyrase point mutation (G88C)	CIP111344	PM_3
M. tuberculosis multi-drug resistant clinical isolate	CIP111418	MDR_4
M. tuberculosis multi-drug resistant clinical isolate	CIP111386	MDR_5
M. tuberculosis multi-drug resistant clinical isolate	CIP111390	MDR_6
M. tuberculosis multi-drug resistant clinical isolate	CIP111392	MDR_7
M. tuberculosis multi-drug resistant clinical isolate	CIP111393	MDR_8
RAW 264.7, mouse macrophages	ATCC	TIB-71
THP-1, human peripheral blood monocytes	ATCC	TIB-202
Vero, grivet kidney epithelial cells	ATCC	CCL-81
**Plasmids**		
pCR2.1-TOPO TA cloning plasmid, Amp^R, Km^R	Invitrogen	pCR2.1-TOPO
pMV361-based integrative vector, containing L5 phage attB-integration site expressing *mCherry* from UV15 strong promoter, Hyg^R	Lab collection	pGM218
pLJR965, tetracycline-inducible dCas9 *attB*-integrative vector for *M. tuberculosis* gene silencing, Km^R	Addgene[9]	#115163
pLJR965 modified with Hyg^R cassette	Lab collection	pGM309
pGM309 carrying sgRNA for *nat* (*rv3566c*) silencing, Hyg^R	This paper	pGM315
pMV361-based integrative vector, containing L5 phage attB-integration site, expressing *nat* (*rv3566c*) from UV15 strong promoter, Hyg^R	This paper	pGM321
pMV361-based integrative vector, containing L5 phage *attB*-integration site, expressing *recA* (*rv2737c*) fused to *mCherry* under the native *recA* dual promoter, and *gfp* from the UV15 strong promoter, Km^R	This paper	pGM322

Supplementary Table 2. Oligonucleotides. Purpose, features, and direction are indicated.

REAGENT	SOURCE	IDENTIFIER
siRNA targeting sequence, sgRNA_*rv3566c – for*<br>5'-GGGAGGACTGTGCACGGTCACCAGATCCT-3'	This paper, IDT	# 230084119
siRNA targeting sequence, sgRNA_*rv3566c – rev*<br>5'-AAACAGGATCTGGTGACCGTGCACAGTCC-3'	This paper, IDT	# 230084120
CT primer silencing, pCRISPRi_Seq – *rev*	Lab collection, Eurofins	# 26983185
CT primer silencing, pLJR_PCR – *for*	Lab collection Eurofins	# 27008276
Cloning, **restriction site**, <u>Shine Dalgarno</u>, *rv3566c*, NAT_oe – *for*<br>5'-AC**TTAATTAA**<u>GAAGGAGATATACAT</u><br>ATGGCACTGGATCTGACCGCG-3'	This paper, IDT	# 232873379
Cloning, **restriction site**, *rv3566c*, NAT_oe – *rev*<br>5'-AT**GTTAAC**TTACGGCGCATCGGCTCCTGG-3'	This paper, IDT	# 232873380
qPCR*, sigA (rv2703) – for*<br>5'-ACGACGAAGACCACGAAGAC-3'	This paper, Eurofins	# 27779941
qPCR*, sigA (rv2703) – rev*<br>5'-CTTCATCCCAGACGAAATCACC-3'	This paper, Eurofins	# 27779942
qPCR*, nat* (*rv3566c*) *– for*<br>5'-GGCTGATGGGTTATGTGCTG-3'	This paper, Eurofins	# 32757720
qPCR*, nat* (*rv3566c) – rev*<br>5'-ATCCGACGTCGACGAGATAG-3'	This paper, Eurofins	# 32757721
qPCR*, ephA* (*rv3617*) *– for*<br>5'-GGCTTTATCGATCGGCTTCC-3'	This paper, Eurofins	# 33124397
qPCR*, ephA* (*rv3617*) *– rev*<br>5'-GTGAACTCGCCGATGTAGTG-3'	This paper, Eurofins	# 33124398
qPCR*, ephD* (*rv2214c*) *– for*<br>5'-TACATGGCCTTGTTCTCGGT-3'	This paper, Eurofins	# 33124399
qPCR*, ephD* (*rv2214c*) *– rev*<br>5'-AGCGTCTCCGAGTGATGAAT-3'	This paper, Eurofins	# 33124401
qPCR*, sodA* (*rv3846*) *– for*<br>5'-CAGCGATCTTGCTGAACGAA-3'	This paper, Eurofins	# 33124401
qPCR*, sodA* (*rv3846*) *– rev*<br>5'-CACGGAACTTGTCGAACGAA-3'	This paper, Eurofins	# 33124402
qPCR*, sodC* (*rv0432*) *– for*<br>5'-CCTTCACCATGGACGACCT-3'	This paper, Eurofins	# 33124403
qPCR*, sodC* (*rv0432*) *– rev*<br>5'-GTCCCATTGACCTGGACGTA-3'	This paper, Eurofins	# 33124404
qPCR*, trxB1*(*rv1471*) *– for*<br>5'-GACTACCCGAGACCTCACTG-3'	This paper, Eurofins	# 33124405
qPCR*, trxB1* (*rv1471*) *– rev*<br>5'-ACCAGGAGGCCCAATAATCG-3'	This paper, Eurofins	# 33124406
qPCR*, ahpE* (*rv2238*) *– for*<br>5'-CGACCAGAATCAGCAGCTTG-3'	This paper, Eurofins	# 33124407
qPCR*, ahpE* (*rv2238*) *– rev*<br>5'-CAACAGCACGTTCTTTGCAC-3'	This paper, Eurofins	# 33124408
qPCR*, katG* (*rv1908c*) *– for*<br>5'-AAGGCCTGGTACAAGCTGAT-3'	This paper, Eurofins	# 33124409
qPCR*, katG* (*rv1908c*) *– rev*<br>5'-GGCTCTTAAGGCTGGCAATC-3'	This paper, Eurofins	# 33124410
qPCR*, recA (rv2737c) – for*<br>5'-CACGACGACCTCTGAACAAC-3'	This paper, Eurofins	# 33124413
qPCR*, recA (rv2737c) – rev*<br>5'-CGTAATCTCGAACGGTGCTC-3'	This paper, Eurofins	# 33124414
qPCR*, mfpA (rv3361c) – for*<br>5'-CGTTGGACGACGTGGATTTC-3'	This paper, Eurofins	# 33124415
qPCR*, mfpA (rv3361c) – rev*<br>5'-TCAAGTTGAGACCACGCAGA-3'	This paper, Eurofins	# 33124416

Supplementary Methods

Three phenanthroline derivatives (**M01 – MR34503; M05 – MR34504; M06 – MR34509**) were formerly published[10], whereas four of them were synthesized in this work. All chemical reagents and solvents were purchased from commercial sources and used without further purification. Melting points were determined on a Kofler melting point apparatus. ^{1}H and ^{13}C NMR spectra were recorded on a BRUKER AVANCE III 400 MHz with chemical shifts expressed in parts per million (in chloroform-*d*) downfield from TMS as an internal standard and coupling in Hertz. IR spectra were recorded on a PerkinElmer BX FT-IR apparatus using KBr pellets. High resolution mass spectra (HRMS) were obtained by electrospray on a BrukermaXis. The purities of all tested compounds were analyzed by LC–MS, with the purity all being higher than 95%. Analyses were performed with a Waters Alliance 2695 as separating module (column XBridge C18 2.5 mM/4.6 x 50 mM) using the following gradients: A (95%)/B (5%) to A (5%)/B (95%) in 4.00 min. This ratio was hold during 1.50 min before return to initial conditions in 0.50 min. Initial conditions were then maintained for 2.00 min (A ¼ H_2O, B ¼ CH_3CN; each containing HCOOH: 0.1%). MS were obtained on a SQ detector by positive ESI.

3-(dimethylamino)propyl 1-methyl-6-nitro-4-oxo-1,4-dihydro-1,10-phenanthroline-3-carboxylate (M02 – MR36009): From 1-methyl-6-nitro-4-oxo-1,10-phenanthroline-3-carboxylic acid(1 eq, 0.33 mmol, 100 mg), dry THF (4 mL), oxalyl chloride (2.5 eq, 0.82 mmol, 69 µL) and DMF (2 drops) according to the general procedure A. The mixture was stirred for 3h. After concentration under pressure, DCM (4 mL), triethylamine (1.3 eq, 0.43 mmol, 60 µL) and 3-(dimethylamino)propan-1-ol (1.3 eq, 0.43 mmol, 51 µL) were stirred for 30 min. The product was washed with sodium carbonate satured water. The organic layer was dried over $MgSO_4$. Removal of the solvant under reduced pressure afforded the crude product, which was purified by chromatography on silica gel column (DCM/MeOH/NH_3, gradient 100:0:0 to 80:20:0.2) to give the compound (24%).^{1}H NMR($CDCl_3$, 400 MHz) δ 9.26 (s, 1H), 9.06 (s, 1H), 9.04 (dd, *J* = 3 Hz, 1H), 8.54 (s, 1H), 7.82 – 7.73 (m, 1H), 4.66 (s, 3H), 4.40 (t, *J* = 6.7 Hz, 2H), 2.51 (t, *J* = 7.3 Hz, 2H), 2.29 (s, 6H), 1.99 (p, J= 6.8 Hz, 2H).13C NMR ($CDCl_3$, 100 MHz) δ 172.3, 164.7, 153.3, 148.5, 142.1, 141.7, 141.3, 132.5, 127.6, 124.7, 123.7, 123.4, 114.4, 63.8, 56.3, 50.6, 45.5, 26.9. MS m/z $[M+H]^+$ 384.87. IR (neat, cm-1) 3063, 2953, 2816, 2766, 2242, 1696, 1639, 1599, 1520, 1501, 1341, 1204, 1089. Mp156.8°C. HRMS m/z $[M+H]^+$ calc 385.1512 exp 385.1513. Mp 156.8°C

2-bromoethyl 1-methyl-6-nitro-4-oxo-1,4-dihydro-1,10-phenanthroline-3-carboxylate (M03 – MR36013): From 1-methyl-6-nitro-4-oxo-1,10-phenanthroline-3-carboxylic acid (1 eq, 0.23 mmol, 69 mg), dry THF (3 mL), oxalyl chloride (2 eq, 0.46 mmol, 39 µL) and DMF (2 drops) according to the general procedure A. After concentration under pressure, DCM (3 mL), triethylamine (1.3 eq, 0.30 mmol, 42 µL) and 2-bromoethanol (1.3 eq, 0.30 mmol, 21 µL) were stirred for 1 h. DCM was then added and the precipitate was filtered under vacuum. Removal the solvent of filtrate under reduced pressure afforded the product, which was purified by chromatography on silica gel column (DCM/AcOEt, gradient 100:0 to 50:50) to give a yellow bright solid (20%). ^{1}H NMR ($CDCl_3$, 400 MHz) δ 9.37 (s, 1H), 9.15 –9.06 (m, 2H), 8.60 (s, 1H), 7.80 (dd, *J* = 8.8, 4.2 Hz, 1H), 4.76 –4.64 (m, 5H), 3.69 (t, *J* = 6.3 Hz, 2H).^{13}C NMR ($CDCl_3$, 100 MHz) δ 172.4, 164.0, 153.5, 148.6, 142.4, 141.8, 141.4, 132.7, 127.9, 124.8, 123.9, 123.5, 113.8, 64.5, 50.8, 28.8. MS m/z $[M+H]^+$ 405.68 - 407.68. IR (neat, cm-1) 3067, 2931, 1734, 1690, 1640, 1622, 1518, 1478, 1370, 1319, 1255, 1198, 1118, 1085. Mp 234.1°C.

***N*, *N*, 1-trimethyl 6-nitro-4-oxo-1,4-dihydro-1,10-phenanthroline-3-carboxylamide (M04 – MR36018):** From 1-methyl-6-nitro-4-oxo-1,10-phenanthroline-3-carboxylic acid (1 eq, 0.23 mmol, 69 mg), dry THF (5 mL), oxalyl chloride (2 eq, 0.46 mmol, 39 µL) and DMF (2 drops) according to the general procedure A. After concentration under pressure, DCM (5 mL), triethylamine (1.3 eq, 0.3 mmol, 42 µL) and dimethylamine (1.3 eq, 0.3 mmol, 15 µL) were stirred for 1 h. DCM was then added and the organic phase was washed once with $NaHCO_3$ sat, dried on $MgSO_4$ and concentrated. The crude product was purified by chromatography on desactivated silica gel column (DCM/MeOH, gradient 100:0 to 80:20) to give a yellow solid (50%). ^{1}H NMR ($CDCl_3$, 400 MHz) δ 9.37 (s, 1H), 9.14 (dd, *J* = 8.8, 1.7 Hz, 1H), 9.07 (dd, *J* = 4.2, 1.7 Hz, 1H), 8.09 (s, 1H), 7.80 (dd, *J* = 8.8, 4.2 Hz, 1H), 4.65 (s, 3H), 3.16 (s, 3H), 3.07 (s, 3H).^{13}C NMR ($CDCl_3$,100 MHz) δ 171.8, 166.2, 149.6, 148.3,

141.9, 141.8, 141.5, 132.7, 125.9, 124.6, 123.8, 123.8, 123.0, 50.2, 38.7, 35.8. MS m/z $[M+H]^+$ 326.91. IR (neat, cm-1) 2963, 2922, 2852, 1719, 1636, 1501, 1384, 1261, 1078.

1-(dimethylamino)prop-2-yl 1-methyl-6-nitro-4-oxo-1,4-dihydro-1,10-phenanthroline-3-carboxylate (M07 – MR36017): From 1-methyl-6-nitro-4-oxo-1,10-phenanthroline-3-carboxylic acid (1 eq, 0.27 mmol, 80 mg), dry THF (5 mL), oxalyl chloride (2 eq, 0.54 mmol, 45 µL) and DMF (2 drops) according to the general procedure A. After concentration under pressure, DCM (5 mL), triethylamine (1.3 eq, 0.35 mmol, 49 µL) and 1-dimethylaminopropan-2-ol (1.3 eq, 0.36 mmol, 43 µL) were stirred for 1 h. DCM was then added and the organic phase was washed once with $NaHCO_3$ sat, dried on $MgSO_4$ and concentrated. The crude product was purified by chromatography on desactivated silica gel column (DCM/MeOH, gradient 100:0 to 80:20) to give a yellow solid (44%). ^1H NMR ($CDCl_3$, 400 MHz) δ 9.35 (s, 1H), 9.10 (dd, *J* = 8.8, 1.7 Hz, 1H), 9.06 (dd, *J* = 4.2, 1.7 Hz, 1H), 8.55 (s, 1H), 7.78 (dd, *J* = 8.8, 4.1 Hz, 1H), 5.41 – 5.28 (m,1H), 4.66 (s, 3H), 2.73 (dd, J = 12.9, 7.3 Hz, 2H), 2.42 (dd, J = 12.9, 5.1 Hz, 1H), 2.32 (s, 6H), 1.40 (d, J = 6.3 Hz, 3H).^{13}C NMR ($CDCl_3$,100 MHz) δ 172.5, 164.2, 153.2, 148.5, 142.1, 141.8, 141.3, 132.7, 127.8, 124.7, 123.8, 123.6, 114.9, 69.7, 64.4, 50.5, 46.3, 46.2, 18.8. MS m/z $[M+H]^+$ 384.94. IR (neat, cm-1) 1712, 1630, 1597, 1501, 1335, 1264, 1200, 1082. HRMS m/z $[M+H]^+$ calc 385.1512 exp 385.1515. Mp 119.7°C

6-Amino-1-ethyl-4-oxo-1,4-dihydro-1,10-phenanthroline-3-carboxylic acid ($M06-NH_2$ – MR38509): Chemical formula: $C_{15}H_{13}N_3O_3$. To a suspension of M01 – MR34503 (1 eq., 1 mmol) in Acetic Acid (6 mL/mmol) is added Fe (6 eq., 6 mmol) by portion. The mixture is heated at 80°C for 2 hrs. After cooling to rt, EtOH is added. The mixture is filtered on celite, washed with EtOH and the filtrate is concentrated in vacuo. Water is added in an ice bath. The precipitate is filtered under reduced pressure and dried to afford ethyl 6-amino-1-ethyl-4-oxo-1,4-dihydro-1,10-phenanthroline-3-carboxylate. The intermediate was suspended in HCl 1N and stirred at 80°C overnight. After completion, the mixture was filtered and rinsed with water to afford the product as a brown solid with 56 % yield. ^1H NMR (500 MHz, Methanol-d_4) δ 9.91 (d, *J* = 3.7 Hz, 1H), 9.65 (d, *J* = 11.7 Hz, 2H, 2 CH), 8.62 (dd, *J* = 8.8, 3.9 Hz, 1H), 8.40 (s, 1H), 8.34 – 8.09 (m, 1H, NH_2), 6.24 (q, *J* = 6.8 Hz, 2H, CH_2), 2.23 (t, *J* = 6.8 Hz, 3H, CH_3). ^{13}C NMR (126 MHz, Methanol-d_4) δ 185.32, 174.06, 158.18, 156.96, 153.27, 150.38, 141.46, 138.46, 137.83, 131.73, 131.60, 117.81, 109.40, 64.87, 26.06. MS m/z $[M+H]^+$ 284.20. IR (neat, cm-1) 3422, 2923, 2852, 1720, 1615, 1420. HRMS m/z $[M+H]^+$ calc 284.1035 exp 284.1031. Mp 224°C.

Molecular descriptors such as cLogP were either calculated using the software DataWarrior[11], or predicted using ADMETlab 2.0[12].

Supplementary References

1. Manina, G., Griego, A., Singh, L. K., McKinney, J. D. & Dhar, N. Preexisting variation in DNA damage response predicts the fate of single mycobacteria under stress. *The EMBO Journal* **38**, (2019).
2. Blower, T. R., Williamson, B. H., Kerns, R. J. & Berger, J. M. Crystal structure and stability of gyrase–fluoroquinolone cleaved complexes from *Mycobacterium tuberculosis*. *Proc. Natl. Acad. Sci. U.S.A.* **113**, 1706–1713 (2016).
3. The Gene Ontology Consortium *et al.* The Gene Ontology resource: enriching a GOld mine. *Nucleic Acids Research* **49**, D325–D334 (2021).
4. Mi, H., Muruganujan, A., Ebert, D., Huang, X. & Thomas, D. PANTHER version 14: more genomes, a new PANTHER GO-slim and improvements in enrichment analysis tools. *Nucleic Acids Research* **47**, D419–D426 (2019).
5. Szklarczyk, D. *et al*. The STRING database in 2023: protein-protein association networks and functional enrichment analyses for any sequenced genome of interest. *Nucleic Acids Res* **51**, D638–D646 (2023).
6. Kapopoulou, A., Lew, J.M. & Cole, S. T. The MycoBrowser portal: a comprehensive and manually annotated resource for mycobacterial genomes. *Tuberculosis (Edinb)* **91**, 8–13 (2011).
7. Abuhammad, A. *et al.* Structure of arylamine *N* -acetyltransferase from *Mycobacterium tuberculosis* determined by cross-seeding with the homologous protein from *M. marinum*: triumph over adversity. *Acta Crystallogr D Biol Crystallogr* **69**, 1433–1446 (2013).
8. Manina, G., Dhar, N. & McKinney, J. D. Stress and host immunity amplify *Mycobacterium tuberculosis* phenotypic heterogeneity and induce nongrowing metabolically active forms. *Cell Host & Microbe* **17**, 32–46 (2015).
9. Rock, J. M. *et al.* Programmable transcriptional repression in mycobacteria using an orthogonal CRISPR interference platform. *Nat Microbiol* **2**, 16274 (2017).
10. Coulibaly, S. *et al.* Phenanthrolinic analogs of quinolones show antibacterial activity against *M. tuberculosis*. *European Journal of Medicinal Chemistry* **207**, 112821 (2020).
11. Sander, T. *et al.* DataWarrior: An open-source program for chemistry aware data visualization and analysis. *J Chem Inf Model* **55**, 460–473 (2015).
12. Xiong, G. *et al.* ADMETlab 2.0: an integrated online platform for accurate and comprehensive predictions of ADMET properties. *Nucleic Acids Res* **49**, W5–W14 (2021).
