# Supplementary material for: Dynamic microfluidic single-cell screening identifies pheno-tuning compounds to potentiate tuberculosis therapy
Source: Nat Commun. 2024 May 16;15:4175. doi: 10.1038/s41467-024-48269-2 (PMC11099131; doi:10.1038/s41467-024-48269-2)
Supplement: Supplementary file 3 — Description of Additional Supplementary Files [file 41467_2024_48269_MOESM3_ESM.pdf]

Description of Additional Supplementary Files

Supplementary Data legends

Supplementary Data 1. Mask design of flow and control layers of the 32-condition device.

Supplementary Data 2. PTC candidates used for the screening. List of fragment compounds and phenanthroline derivatives used in this study (Fig. 1). The table reports: structures; simplified molecular-input line-entry systems; names; molecular weights; abbreviations used in the screening; MIC in mycobacteria; IC50 in Vero cells; either calculated (https://openmolecules.org/datawarrior/)[11] or predicted (https://admetmesh.scbdd.com)[12] LogP values; and other known properties. The logP value of a compound is the logarithm of its partition coefficient between n-octanol and water and provides a measure of its hydrophilicity.

Supplementary Data 3. Screening results; extracted parameters and microcolony indices. Output datasheets derived from the analysis of 15 µDeSCRiPTor experiments (Fig. 1). Each experiment is identified by an alphanumeric code and three files per experiment are present, reporting different parameters over time: microcolony area in pixels (area); log_{10} transformed RecA-GFP mean fluorescence (mean); and log_{10} transformed fluorescence variance (var) in arbitrary units. Column B of each datasheet reports time relative to the onset of drug injection, in hours. Column C indicates the absence or presence of treatment. The following columns report the parameter for different colonies, identified in the first row by condition and number. The following datasheet, named cent_results, reports an assembly of all screening results listed in previous datasheets as follows: experiment ID; condition; exposure stage; average of each index across the four last time points of the PRE stage and across the four last time points of the DUR stage for each microcolony. The last two datasheets, named effect_sizes_mean or var, contain the outcome of effect size estimations. *P* value was calculated based on a two-sided Wald test. *P* values in this table were not adjusted to account for multiple testing.

Supplementary Data 4. Differential analysis of *M. tuberculosis* transcriptome upon PTC exposure. Raw and normalized counts per gene in each condition tested and in the control condition DMSO ($n = 3$). Fold changes and relative statistics, Wald test *P* values and Benjamini-Hochberg corrected *P* values (padj) are also reported for each comparison. Two datasheets are provided for each PTC hit (Fig. 2). The first datasheet includes upregulated genes (UP) and the second one includes downregulated genes (DN).

Supplementary Data 5. Functional and GO enrichment analyses of DEGs upon PTC exposure. Two datasheets are included for each condition: functional analysis (FA) and gene ontology enrichment (GO) (Fig. 2 and Supplementary Fig. 6). Each FA datasheet reports merged differential analysis of upregulated (black font, UP) and downregulated (white font, DN) genes, sorted by functional category using Mycobrowser's color coding (https://mycobrowser.epfl.ch)[6]. Each GO datasheet reports GO biological processes identified by PANTHER overrepresentation test of significant DEGs against all genes in *M. tuberculosis* H37Rv database[3,4]. Significance was assessed by Fisher's exact test followed by Benjamini-Hochberg procedure, filtering valid results for FDR < 0.05. Results are sorted by fold enrichment of the most specific categories (highlighted), with their parent terms indented below. Datasheet names include the type of analysis, the condition, and the direction of DEGs.

Supplementary Data 6. List and features of *gyrA* point mutants and multi-drug resistant strains. The table shows the source, identifier, name, susceptibility profile and associated mutations of strains shown in Fig. 2e.

Supplementary Data 7. Single nucleotide polymorphisms (SNP) of PTC-resistant mutants. Results of variant calling analysis relative to four spontaneous mutants isolated on M02 or M06. Features and effects of each SNP are reported, compared to the parent strain. A summary of these results is shown in Fig. 3a.

Supplementary Data 8. LC-MS analysis. Representative UV chromatograms as a function of retention time of *M. tuberculosis* supernatant (Fig. 3f) and cell extract (Supplementary Fig. 7f), before and after treatment with M06 (15-fold MIC). The experiment was repeated twice with similar results.

Supplementary Movie legends

Supplementary Movie 1. Time-lapse microscopy of wild-type *M. smegmatis* growing in the 32-condition platform. Exponential-phase bacteria growing inside a random microchamber of the 32-condition platform and fed by constant flow of fresh 7H9 medium. Images were recorded at 20-minute intervals (10 fps). Time is shown in hours and minutes. Scale bar = 5 µm.

Supplementary Movie 2. **Combined time-lapse microscopy of *M. smegmatis* RecA-GFP_mCherry$_{cyt}$ reporter during the µDeSCRiPTor.** Exponentially growing bacteria seeded into the 32-condition platform during the screening. Bacteria were first grown in fresh 7H9 medium between 4 to 6 hours. Next, bacteria were treated for 6 hours with subinhibitory concentrations of control compounds (DMSO, MIT, MOX and INH); PTC hits (M01, M02, M04 and M06); or two PTC (B02 and F05) causing a significant but opposite effect to that of PTC hits. Finally, fresh 7H9 medium was perfused everywhere for 6 hours. Images were recorded every 30 minutes (10 fps). Phase contrast (red) and RecA-GFP (cyan) channels are merged. Time is shown in hours and minutes, and conditions are indicated. Scale bars = 5 µm.

Supplementary Movie 3. Time-lapse microscopy of RecA-mCherry_GFP$_{cyt}$ *M. tuberculosis* treated with INH. Exponential-phase bacteria growing inside the hexa-device[1] fed by 7H9 medium, then treated for 2 days with INH (0.2 µg/mL), and finally washed with fresh 7H9 medium. The microcolony was selected for the presence of bacilli that regrew during the washing stage. In the last time frame the microcolony is stained with DRAQ7 (3 µM) to point out permeabilized bacilli. Images were recorded at 3-hour intervals (5 fps). Time is shown in hours. Scale bar = 5 µm.

Supplementary Movie 4. Time-lapse microscopy of RecA-mCherry_GFP$_{cyt}$ *M. tuberculosis* treated with M06 and INH. Exponential-phase bacteria growing inside the hexa-device[1] fed by 7H9 medium, pre-treated for 2 days with M06 (1.25 µg/mL), then treated for 2 more days with M06 (1.25 µg/mL) and INH (0.2 µg/mL), and finally washed with fresh 7H9 medium. The microcolony was selected for the presence of a single bacillus that regrew during the washing stage. In the last time frame the microcolony is stained with DRAQ7 (3 µM) to point out permeabilized bacilli. Images were recorded at 3-hour intervals (5 fps). Time is shown in hours. Scale bar = 5 µm.
