# Supplementary figures and images for: Dynamic microfluidic single-cell screening identifies pheno-tuning compounds to potentiate tuberculosis therapy
Source: Nat Commun. 2024 May 16;15:4175. doi: 10.1038/s41467-024-48269-2 (PMC11099131; doi:10.1038/s41467-024-48269-2)

Flow layer

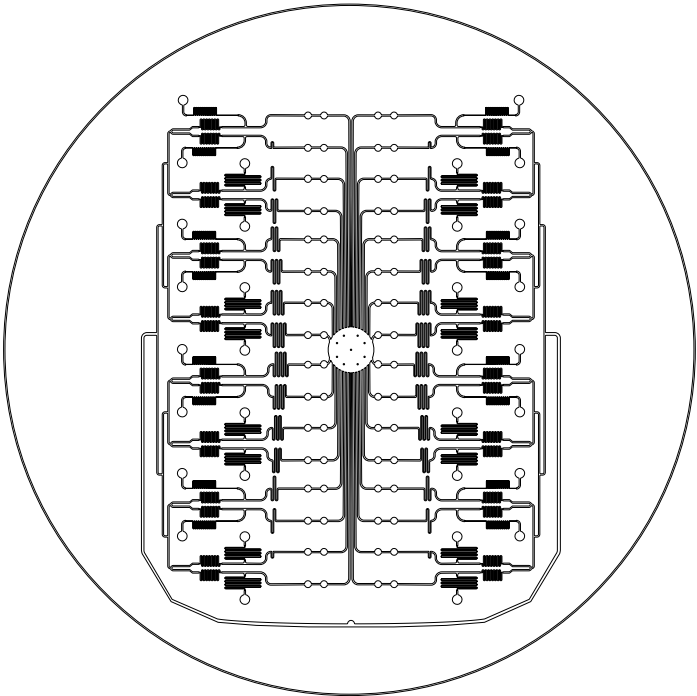

Control layer

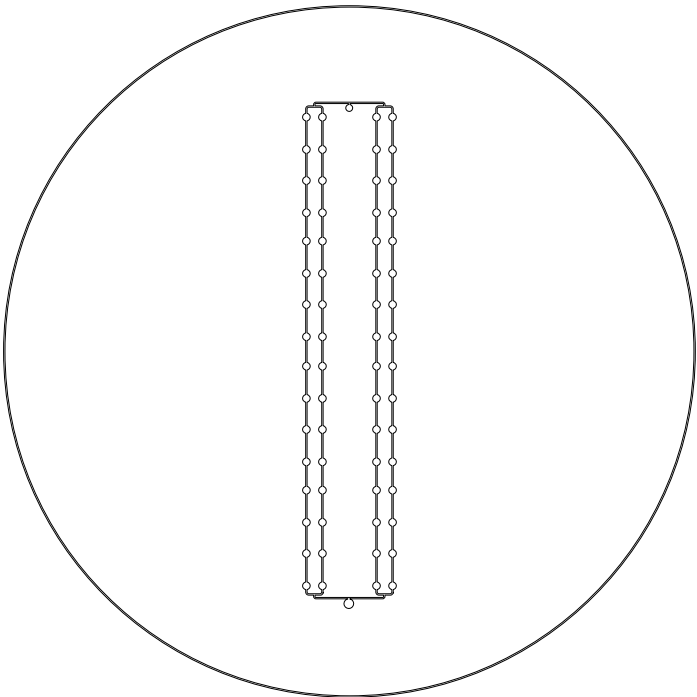

Supplement: Supplementary file 4 — Supplementary Data 1 [file 41467_2024_48269_MOESM4_ESM.pdf]
