# Supplementary material for: Dynamic microfluidic single-cell screening identifies pheno-tuning compounds to potentiate tuberculosis therapy
Source: Nat Commun. 2024 May 16;15:4175. doi: 10.1038/s41467-024-48269-2 (PMC11099131; doi:10.1038/s41467-024-48269-2)
Supplement: Supplementary file 16 — Reporting Summary [file 41467_2024_48269_MOESM16_ESM.pdf]

nature portfolio

Corresponding author(s): Giulia Manina

Last updated by author(s): Apr 9, 2024

Reporting Summary

Nature Portfolio wishes to improve the reproducibility of the work that we publish. This form provides structure for consistency and transparency in reporting. For further information on Nature Portfolio policies, see our Editorial Policies and the Editorial Policy Checklist.

Statistics

For all statistical analyses, confirm that the following items are present in the figure legend, table legend, main text, or Methods section.

n/a	Confirmed	
☐	☒	The exact sample size (*n*) for each experimental group/condition, given as a discrete number and unit of measurement
☐	☒	A statement on whether measurements were taken from distinct samples or whether the same sample was measured repeatedly
☐	☒	The statistical test(s) used AND whether they are one- or two-sided<br>*Only common tests should be described solely by name; describe more complex techniques in the Methods section.*
☐	☒	A description of all covariates tested
☐	☒	A description of any assumptions or corrections, such as tests of normality and adjustment for multiple comparisons
☐	☒	A full description of the statistical parameters including central tendency (e.g. means) or other basic estimates (e.g. regression coefficient) AND variation (e.g. standard deviation) or associated estimates of uncertainty (e.g. confidence intervals)
☐	☒	For null hypothesis testing, the test statistic (e.g. *F*, *t*, *r*) with confidence intervals, effect sizes, degrees of freedom and *P* value noted<br>*Give P values as exact values whenever suitable.*
☒	☐	For Bayesian analysis, information on the choice of priors and Markov chain Monte Carlo settings
☐	☒	For hierarchical and complex designs, identification of the appropriate level for tests and full reporting of outcomes
☐	☒	Estimates of effect sizes (e.g. Cohen's *d*, Pearson's *r*), indicating how they were calculated

Our web collection on statistics for biologists contains articles on many of the points above.

Software and code

Policy information about availability of computer code

Data collection	The following commercial or open source software were used to collect data:<br>softWoRx 7.0 (Cytiva); Amersham Typhoon control software (Cytiva); ChemiDoc XRS Quantity One Analysis Software (Bio-Rad); Biohit Microplate Reader integrated software; Roche LightCycler 480 integrated software; Chromeleon Chromatography Data System software (Thermo Scientific); GloMax Discover software (Promega).
Data analysis	The following commercial or open source software were used to analyze data: ImageJ (2.0.0-rc-59/1.51n); R software 4.0.2; Microsoft Excel; GraphPad Prism 9.5.0; Sequana 0.9.8; Snakemake 6.1.1; bwa 0.7.17; Sambamba 0.8.0; Freebayes 1.3.2; SNPeff 5.0; multiQC 1.10.1; Snakemake 5.8.1; Cutadapt 2.10; bowtie 2.2.2; featureCounts 2.0.0; MultiQC 1.8; DESeq2 library 1.24.0 with SARTools 1.7.0; GO enrichment analysis tool PANTHER [http://geneontology.org/]; SeamDock docking web server [https://seamless.rpbs.univ-paris-diderot.fr/cloudless/instance/6467781/ctx/index.html]. In addition, we developed functions and scripts for the analysis of the PTC screening [https://gitlab.pasteur.fr/svolant/ptc-screening/]; an Omnipose model and Python notebook for the analysis of single-cell snapshots [https://gitlab.pasteur.fr/iah-public/automated_segmentation_mycobacterium_tuberculosis_snapshots]; and an ImageJ macro (Macro_Analysis_THP1_MTUB.ijm), for the analysis of infected macrophages [https://doi.org/10.5061/dryad.r4xgxd2j8].

For manuscripts utilizing custom algorithms or software that are central to the research but not yet described in published literature, software must be made available to editors and reviewers. We strongly encourage code deposition in a community repository (e.g. GitHub). See the Nature Portfolio guidelines for submitting code & software for further information.

Data

Policy information about availability of data

All manuscripts must include a data availability statement. This statement should provide the following information, where applicable:

- Accession codes, unique identifiers, or web links for publicly available datasets
- A description of any restrictions on data availability
- For clinical datasets or third party data, please ensure that the statement adheres to our policy

We have no restriction on data availability. Source data are provided with this paper. Time-lapse image stacks and snapshot images of mycobacterial cells alone or during infection under drug treatment generated in this study have been deposited in the DRYAD database under the unique digital object identifier DOI: 10.5061/dryad.r4xgxd2j8 [https://doi.org/10.5061/dryad.r4xgxd2j8]. Raw data from whole transcriptome and whole genome sequencing have been deposited in EMBL's European Bioinformatics Institute, under the EBI accession codes E-MTAB-12306 (RNA-seq) and E-MTAB-12307 (WGS) [https://www.ebi.ac.uk/biostudies/studies/E-MTAB-12306; https://www.ebi.ac.uk/biostudies/studies/E-MTAB-12307]. The processed data derived from single cells, microcolonies, CFU, MIC, TLC, LC-MS, and RNA/DNA-Seq generated in this study are provided in the Supplementary Information/Source Data file. The M. tuberculosis PANTHER genome information used in this study is available in the GO Ontology database under the accession code MYCTU [https://pantherdb.org/genomes/genome.jsp?taxonId=83332]. The structure of the arylamine N-acetyltransferase used in this study is available at the RCSB Protein Data Bank (RCSB PDB) under the accession code 4BGF [https://doi.org/10.2210/pdb4BGF/pdb]. The structure of the DNA gyrase from M. tuberculosis used in this study is available at the RCSB PDB under the accession code 5BS8 [https://doi.org/10.2210/pdb5BS8/pdb]. The interaction network of M. tuberculosis NAT used in this study is available at the STRING Core Data Resource [https://string-db.org/cgi/network?taskId=bvahymk8E36h&sessionId=bAEKEPVHSkc2].

Research involving human participants, their data, or biological material

Policy information about studies with human participants or human data. See also policy information about sex, gender (identity/presentation), and sexual orientation and race, ethnicity and racism.

Reporting on sex and gender	N/A
Reporting on race, ethnicity, or other socially relevant groupings	N/A
Population characteristics	N/A
Recruitment	N/A
Ethics oversight	N/A

Note that full information on the approval of the study protocol must also be provided in the manuscript.

Field-specific reporting

Please select the one below that is the best fit for your research. If you are not sure, read the appropriate sections before making your selection.

☒ Life sciences ☐ Behavioural & social sciences ☐ Ecological, evolutionary & environmental sciences

For a reference copy of the document with all sections, see nature.com/documents/nr-reporting-summary-flat.pdf

Life sciences study design

All studies must disclose on these points even when the disclosure is negative.

Sample size	In Fig. 1, sample size refers to the number of time points measured for each molecule. For each phase (before, during, and after), at least 6 time points were measured for each microcolony. For other single-cell experiments, which are poorly automated, we used samples of a few hundred bacteria and a few thousand macrophages. As for bulk-cell experiments and omics, the sample size was typically 3. Sample size was primarily determined by considering both experimental and analytical capacity, ensuring that a minimum size was attained to evaluate statistical significance and enable proper interpretation of the results. In addition, the sample sizes used in this study are in line with experimental standards in the field.
Data exclusions	Images with poor resolution were excluded from the analysis.
Replication	In Fig. 1, each molecule was replicated up to 8 times for each experiment, and several experiments were performed. All other experiments were replicated from a minimum of 2 (if highly reproducible) up to a maximum of 6 times.
Randomization	Randomization does not apply to this study because of the relative homogeneity of the samples under controlled experimental conditions. The experimental design defines the groups of samples based on criteria such as the absence or presence of treatment, different fluorescence levels, or different experimental time points.

Blinding	Blinding does not apply to this study, which is based on predefined experimental set-ups and sample categories, and objective quantitative measurements.

Reporting for specific materials, systems and methods

We require information from authors about some types of materials, experimental systems and methods used in many studies. Here, indicate whether each material, system or method listed is relevant to your study. If you are not sure if a list item applies to your research, read the appropriate section before selecting a response.

Materials & experimental systems

n/a	Involved in the study	
☒	☐	Antibodies
☐	☒	Eukaryotic cell lines
☒	☐	Palaeontology and archaeology
☒	☐	Animals and other organisms
☒	☐	Clinical data
☐	☒	Dual use research of concern
☒	☐	Plants

Methods

n/a	Involved in the study	
☒	☐	ChIP-seq
☒	☐	Flow cytometry
☒	☐	MRI-based neuroimaging

Eukaryotic cell lines

Policy information about cell lines and Sex and Gender in Research

Cell line source(s)	RAW 264.7 macrophages (ATCC TIB-71), Vero cells (ATCC CCL-81), and THP-1 (TIB-202) were directly purchased from the American Type Culture Collection.
Authentication	Cell lines were visually inspected after purchase and culturing, and they behaved as expected.
Mycoplasma contamination	Cell lines tested negative for mycoplasma contamination.
Commonly misidentified lines (See ICLAC register)	The cell lines used in this work are not included in the ICLAC list.

Dual use research of concern

Policy information about dual use research of concern

Hazards

Could the accidental, deliberate or reckless misuse of agents or technologies generated in the work, or the application of information presented in the manuscript, pose a threat to:

No	Yes	
☐	☒	Public health
☒	☐	National security
☐	☒	Crops and/or livestock
☒	☐	Ecosystems
☒	☐	Any other significant area

Hazards	The pathogen Mycobacterium tuberculosis.

For examples of agents subject to oversight, see the United States Government Policy for Institutional Oversight of Life Sciences Dual Use Research of Concern.

Experiments of concern

Does the work involve any of these experiments of concern:

No	Yes	
☒	☐	Demonstrate how to render a vaccine ineffective
☐	☒	Confer resistance to therapeutically useful antibiotics or antiviral agents
☒	☐	Enhance the virulence of a pathogen or render a nonpathogen virulent
☒	☐	Increase transmissibility of a pathogen
☒	☐	Alter the host range of a pathogen
☒	☐	Enable evasion of diagnostic/detection modalities
☒	☐	Enable the weaponization of a biological agent or toxin
☒	☐	Any other potentially harmful combination of experiments and agents

Precautions and benefits

Biosecurity precautions	Manipulation of live Mycobacterium tuberculosis strains was carried out exclusively in Biosafety Level 3 laboratories, complying with the biosafety standards in place at the Institut Pasteur in Paris and at the Comenius University in Bratislava. Strict implementation of biosecurity procedures such as pathogen containment, disinfection, and waste decontamination significantly reduces the risk to public health or livestock.
Biosecurity oversight	Risk Prevention Officers are in charge of biosafety surveillance, biosafety training, and issuing access authorizations to the BSL3 environment.
Benefits	Further application or use of this work may lead to improved anti-tubercular therapy, with a positive impact on global health.
Communication benefits	Reassure the population that BSL3 research is carried out in safe conditions and under strict control to mitigate risks and to gain maximum benefit from the results obtained.

Plants

Seed stocks	N/A
Novel plant genotypes	N/A
Authentication	N/A
